# The global burden of cognitive impairment in people with HIV

**DOI:** 10.1097/QAD.0000000000003379

**Published:** 2022-09-15

**Authors:** Lea D. Keng, Alan Winston, Caroline A. Sabin

**Affiliations:** a Institute for Global Health, University College London; b Department of Infectious Disease, Imperial College London, London, UK.

**Keywords:** cognitive impairment, global burden, HIV, meta-analysis, systematic review

## Abstract

While life expectancies of people with HIV (PWH) have increased through the successes of antiretroviral treatment, cognitive impairment remains a pressing concern. Prevalence estimates vary worldwide as different definitions for cognitive impairment are used and resource availability differs across geographical settings. We aim to explore this heterogeneity and estimate the global cognitive impairment burden in PWH.

**Design:**

Systematic literature review and meta-analysis.

**Methods:**

We searched PubMed, Embase, SCOPUS, and Web of Science for studies reporting on cognitive impairment prevalence in PWH. Nine factors were investigated for their potential association with the prevalence using a univariate meta-analysis and a meta-regression: assessment method, geographical region, country income, exclusion criteria, study quality, age, sex, publication year, and sample size.

**Results:**

The literature search identified 8539 records, of which 225 were included. The adjusted prevalence was significantly lower in males than females. Across 44 countries, 12 assessment methods were used; the HIV-associated neurocognitive disorder/Frascati criteria, known for high false-positive rates, was employed in 44.4% of studies. The pooled cognitive impairment prevalence estimate in PWH, including asymptomatic cases, was 39.6% (95% confidence interval: 37.2–42.1%; range: 7–87%). The meta-regression explained 13.3% of between-study variation, with substantial residual heterogeneity (*I*^2^ = 97.7%).

**Conclusion:**

Lack of data from more than 70% of the world's countries, cohorts being unselected for symptoms in most research studies, and limitations of the HIV-associated neurocognitive disorder/Frascati criteria restrict the ability to accurately determine the global burden of cognitive impairment in PWH. More studies in low-resource settings and a standardized approach to assessing cognitive impairment, bridging research and clinical realms, are needed.

## Introduction

Since the advent of antiretroviral therapy (ART), the mortality rate in people with HIV (PWH) has significantly reduced. Despite the prevailing treatment gap between resource-limited settings and high-income countries, HIV is now considered a manageable chronic disease [1]. Attention is shifting towards ensuring that increased life expectancy in PWH constitutes healthy life-years. Comorbid conditions, especially non-communicable conditions associated with ageing, have become a focal point; this includes cognitive impairment.

While the prevalence of severe cognitive impairment in PWH has dropped in the past two decades, milder forms are increasingly being recognized [2]. As the life expectancy of PWH nears that of HIV-negative individuals, PWH become equally vulnerable to age-associated cognitive impairment [2,3]. Ageing and comorbidities increase the risk for cognitive dysfunction in older individuals, although milder cognitive impairment is prevalent in younger PWH, suggesting an additional age-independent component. A comparative cognitive impairment analysis in PWH in the pre and post-ART eras, found worse impairments in motor skills and processing speed pre-ART, while people post-ART had impaired memory and executive function [3]. Anthony and Bell [4] found that the neuroinflammation site in the pre-ART era was the basal ganglia, which gradually shifted to predominantly involve the hippocampus and temporal cortex post-ART. These findings suggest that there has been a change to the predominant form of cognitive impairment in the post-ART era.

The optimal approach to cognitive impairment diagnosis in PWH is debated, with a range of assessment methods used across the globe. The gold standard for diagnosis in research settings requires the completion of a neuropsychological test battery, covering multiple domains, and score-based classification criteria. These are hereafter classified as ‘diagnostic’ methods, while faster and less rigorous alternatives are referred to as ‘screening’ methods (Table 1, adapted from Winston and Spudich [5]). A frequently used criterion is the HIV-associated neurocognitive disorder (HAND) criteria, often known as the Frascati criteria [6]. Importantly, in the literature, the term HAND has also been misused as a term for cognitive impairment in PWH when not referring to the HAND criteria. Furthermore, such so-termed ‘diagnostic’ approaches are often used in research settings where no clinical indication exists for the test to be performed. Guidelines for clinical assessments also include magnetic resonance imaging, cerebrospinal fluid analyses, and assessment of patient history, alongside neuropsychological test batteries [7]. As highlighted by others [7,8], there is a lack of consensus on diagnostic methods between and within research and clinical settings.

**Table 1:** Neuropsychological assessment method and criteria.

TYPE and NAME	DESCRIPTION
DIAGNOSTIC i.e. using a neuropsychological test battery	
HAND/Frascati	Separated into three categories based on extent of impairment and impact on daily functioning
	1. Asymptomatic neurocognitive disorder (ANI):
	• 1 SD below normative mean in ≥ 2 cognitive domains
	• No interference with daily functioning
	2. Mild neurocognitive disorder (MND)
	• 1 SD below normative mean in ≥ 2 cognitive domains
	• Mild interference with daily functioning
	3. HIV-associated dementia (HAD)
	• 2 SD below normative mean in ≥ 2 cognitive domains
	• Considerable interference with daily functioning
Gisslen	As HAND/Frascati, but increasing SD cut-offs by 0.5 for ANI and MND
T-Score	Type of standard score, transformed from z-score
	• Impairment if score < 40
GDS (Global Deficit Score)	Scale of 0–5 relative to T-score for each domain
	• Impairment if average across all domains is ≥ 0.5
Clinical ratings	Scale of 1–9 relative to T-score for each domain
	• Impairment if ≥ 2 cognitive domains are ≥ 4
MNC (Multivariate Normative Comparison)	Requires a study-specific control group that acts as a reference
	Uses the multivariate Hotelling's T^2^ statistic and considers all cognitive domains
NMM (Novel Multivariate Method)	Uses the multivariate Mahalanobis distance statistic and considers all cognitive domains
SCREENING i.e. less rigorous, but faster methods	
IHDS / HDS (International HIV Dementia Scale)	Includes three domains tested, (1) motor speed, (2) psychomotor speed and (3) memory
	• Scale of 0–12, impairment if score ≤ 10 (some variability in cut-off across different settings)
	• Completion time: 5 minutes
MoCA Montreal Cognitive Assessment	30-point questionnaire, more sensitive in recognizing mild CI forms
	• Impairment if score < 26 (some variability in cut-off across different settings)
	• Completion time: 10 minutes
MMSE (Mini-Mental State Examination)	30-point questionnaire, more sensitive in recognizing severe CI cases
	• Impairment if score < 24 (some variability in cut-off across different settings)
	• Completion time: 10 minutes
BNCS (Brief Neurocognitive Screen)	Three cognitive tests focusing on processing speed, attention and working memory
	• Impairment if 1 SD below normative mean in≥ 2 cognitive tests or 2 SD below normative mean in one cognitive test
	• Completion time: 15 minutes
CSID (Community Screening Interview for Dementia)	Includes two parts, (1) cognitive test and (2) interview assessing daily functioning adaptable to the sociocultural setting
	• Completion time: 30 minutes

Various approaches are used to identify cognitively impaired individuals, classified here into diagnostic and screening techniques. Cut-offs are stricter in diagnostic approaches compared to screening methods. ANI, asymptomatic neurocognitive disorder; CI, confidence interval; HAD, HIV-associated dementia; HAND, HIV-associated neurocognitive disorder; MND, mild neurocognitive disorder.

Given the time taken to undertake full neuropsychological test batteries, screening methods have been developed which are less time-intensive and resource-intensive. In some resource-limited regions, these less rigorous methods are used for diagnostic purposes [9–11], increasing the risk of false positive diagnoses [12], and potentially increasing the strain on vulnerable health systems. Early and accurate detection is necessary, requiring sufficiently sensitive, validated, and specific approaches [13]. The various cut-offs and dichotomization of cognitive scores further complicate the comparability of cognitive impairment detection methods.

Two recently published articles have investigated cognitive impairment prevalence in subpopulations of PWH: one predominantly considered cases of HAND [14], and the other focussed on PWH aged at least 50 years [15]. To our knowledge, there has not been a systematic review of the evidence on the global burden of cognitive impairment, without restriction on age, geography, or detection method. It is this gap that we aim to fill.

## Methods

### Systematic literature review

The current study was carried out based on the Preferred Reporting Items for Systematic Reviews and Meta-Analyses (PRISMA) statement, which sets out reporting guidelines [16]. Searches were performed in June 2021, in four databases: PubMed, Embase, SCOPUS, and Web of Science. The following Boolean search query was used:

(neurocogniti∗ OR cogniti∗) AND (impairment OR function OR dysfunction OR disorder) AND (hiv OR aids) AND (prevalence OR frequency OR incidence OR rate OR percentage OR proportion OR cohort OR burden OR morbidity OR epidemi∗)

Screening was based on title and abstract in the first screening round, then the full article in the second round. This was done using the online tool Rayyan [17]. Records up to 27 June 2021, were considered for inclusion.

### Inclusion and exclusion criteria

Included studies were required to measure and report the prevalence of cognitive impairment in PWH, and provide details of the neuropsychological assessment method used. To be included, studies were also required to base their estimate on at least 50 participants, who were adults (≥18 years old). Where identical cohorts or recruitment sites were reported in multiple studies, one study was chosen based on three criteria, listed by decreasing importance: most complete report of participant demographics, largest sample size, and most recently published. Studies published in English, German or Chinese were considered.

Studies were excluded if they were a secondary analysis (e.g. a review) of published data or were published in the ‘grey’ literature (e.g. conference abstracts). Studies focusing on individual cognitive domains or self-reported cognitive functioning (e.g. quality of life assessments), or on specific subpopulations of PWH (e.g. people using drugs, those with co-infections, those with depression) were also excluded.

### Risk of bias assessment

The Newcastle-Ottawa scale (NOS), a critical appraisal tool, was used to assess the risk of bias by considering three criteria: cohort selection, participant comparability, and outcome data derivation [18]. Scores of at least 6 were considered good quality.

### Data extraction

Information collected from each study included author(s), publication year, country, number of participants (sample size), cognitive impairment prevalence, neurological assessment method, participants’ demographics (age, sex), and participants’ clinical characteristics (ART exposure). Age was collected as a mean (SD) or median (interquartile range), when available. Each study's exclusion criteria, place of recruitment or cohort name, and recruitment year were recorded (Supplementary Table 1). Data extraction and quality assessment were conducted by L.D.K.

### Statistical analysis

STATA software (Stata/MP-16.1; StataCorp LLC, College Station, Texas, USA) was used for statistical analysis. Cognitive impairment prevalence was calculated with 95% confidence intervals (CIs) and standard errors generated using the exact method (Clopper–Pearson). Due to high heterogeneity between studies, the random effects model and restricted maximum likelihood method were used; significant heterogeneity was defined based on a *Q*-test *P* value less than 0.10 or *I*^2^ value of more than 50% [19,20].

Studies were stratified by neurological assessment method, geographical region, country income level, exclusion criteria, and study quality, and were categorized by neuropsychological assessment criteria, and method (diagnostic/screening). Geographical regions and income levels were assigned based on categorizations of The World Bank. Exclusion criteria of the studies were categorized into: ‘yes’ – exclusion of confounding comorbidities (including neurological and psychiatric disorders, opportunistic central nervous system infections, brain injuries, learning disabilities, and active substance use), ‘partial’, ‘no’ – no exclusion criteria, and ‘unspecified’.

Following univariate analysis, a random-effects meta-regression was conducted. Covariates considered a priori were geographical region, assessment method, country income level, exclusion criteria, study quality, age, ART exposure, sex, publication year, and sample size. Both ART coverage and publication year indicate ART availability among study participants. To avoid collinearity and as 44 studies failed to report their participants’ ART coverage, publication year was used in the meta-regression.

Funnel plots, alongside Egger's and Begg's tests, were used to assess publication bias and detect small-study effects.

## Results

### Literature search

**Fig. 1:**
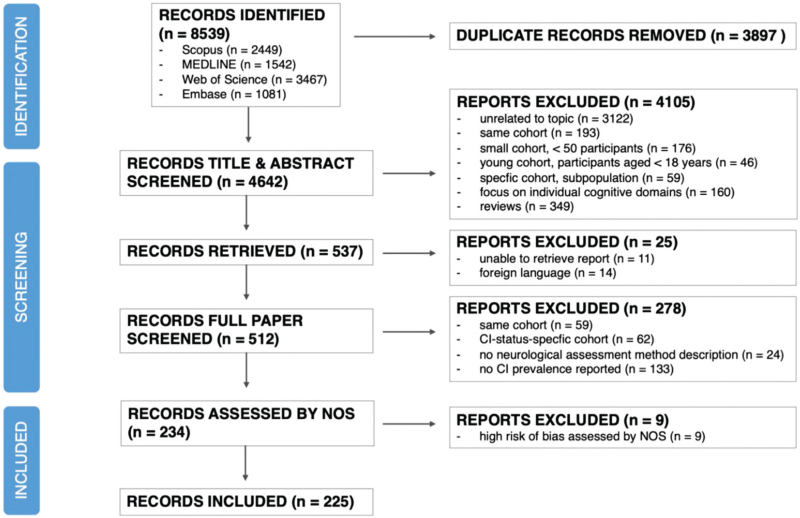
Preferred Reporting Items for Systematic Reviews and Meta-Analyses flowchart.

Searching the databases yielded 8539 records of which 225 were eligible for the meta-analysis (Fig. 1, Supplementary Table 2). Studies were published between 1989 and 2021. Most studies (70%) were from North America, Europe, and sub-Saharan Africa, with only one study from the Middle East and North Africa region. One-hundred and sixty studies used diagnostic methods and 59 studies reported screening methods; a further six studies used uncommon approaches. As expected, income levels did not vary significantly within each geographical region but did vary between regions. The distribution of the different study exclusion criteria was similar across all regions. Most studies (70%) reported some form of exclusion for confounding factors. The rest had no exclusion criteria (30 studies) or did not specify any form of criteria (38 studies). Study participants were generally male and on ART, although this did vary with only 37.4% of study participants from sub-Saharan Africa being male and the lowest ART coverage (59.4%) being reported from South Asia, where all studies were from India.

The flowchart visualizes the steps taken to narrow down from 8539 records, initially identified across four databases, to 225 eligible articles for meta-analysis. (See methods for details on inclusion and exclusion criteria.)

### Univariate-analysis

The pooled cognitive impairment prevalence (95% CI) from all studies was 39.6% (37.2–42.1%), including asymptomatic symptoms. High between-study heterogeneity was observed (*I*^2^ = 98.16%).

When sub-grouped by geographical region, there was significant between-subgroup heterogeneity (*P* = 0.01, Supplementary Figs. 1–8). Of note, many countries within each geographical region are not represented in Fig. 2 due to a lack of data. Moreover, in a separate analysis, studies were sub-grouped by individual criteria (i.e. cognitive testing tools), then by assessment method (diagnostic vs. screening). Although there was substantial heterogeneity within each subgroup, both for assessment type and individual criteria at 0.01 significance level, there was no significant difference between assessment types, *P* = 0.20 (Supplementary Figs. 9–12). Between-subgroup heterogeneity was also significant when studies were sub-grouped by country income level (*P* = 0.01) as well as by study exclusion criteria or by NOS score, both at significance level 0.10 (Supplementary Figs. 13–27).

**Fig. 2:**
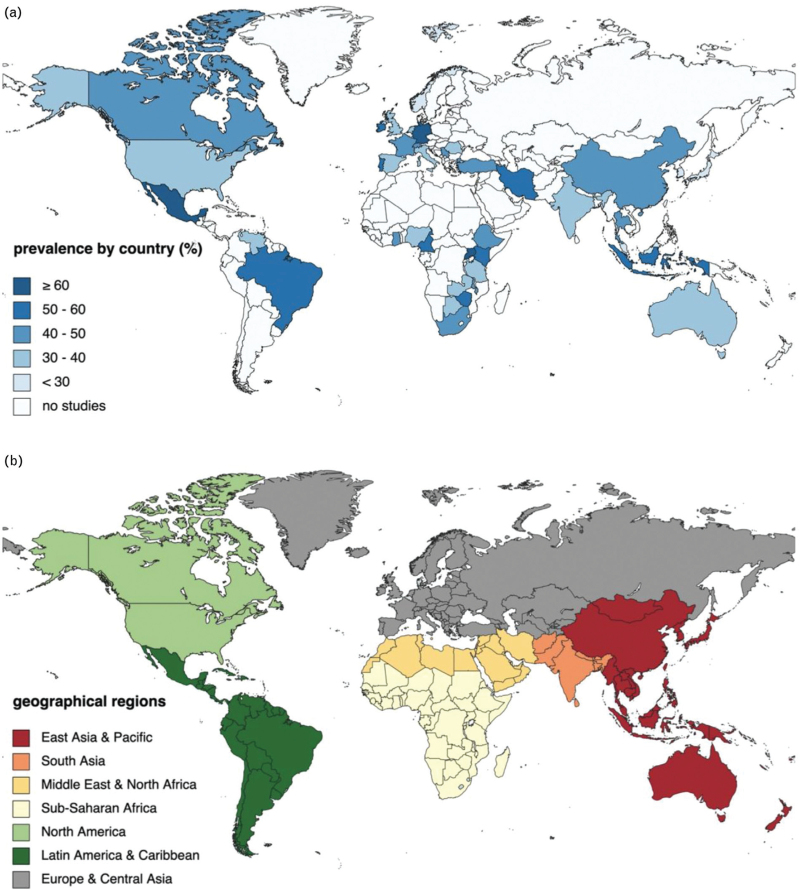
Global burden of cognitive impairment in people with HIV.

(a) Cognitive impairment prevalence by country. (b) The World Bank geographical regions. The 225 studies span 44 countries. When stratifying by geographical regions, some regions are represented by a limited number of countries. Figure made using mapchart.net.

A slight negative correlation was found between cognitive impairment and the proportion of men in the cohort (Supplementary Fig. 28); participant sex was reported in 221 studies and in these, the participants were predominantly male. No correlation was seen between cognitive impairment and the following variables: ART coverage, age, publishing year, or sample size (Supplementary Figs. 29–32).

### Multivariate meta-regression

The meta-regression model considered nine covariates, involving 22 parameters; of these, only sex was significantly associated with cognitive impairment in PWH (*P* = 0.003, Table 2). The model considered 204 studies; 21 studies lacked data for at least one of the covariates. There was a negative correlation between the percentage of men in the cohort and the proportion with cognitive impairment. The meta-regression model explained 13.3% of the variation in prevalence between studies, but there remained substantial heterogeneity (*R*^2^ = 13.3%, *I*^2^ = 97.7%).

**Table 2:** Results from meta-regression to identify factors associated with the reported prevalence of cognitive impairment.

Variable	Coefficient	95% CI	*P* value	
Geographical region (country)				
North America	Reference			
Latin America and the Caribbean	0.082	−0.081 to 0.244	0.325	0.177
Europe and Central Asia	−0.042	−0.117 to 0.033	0.275	
East Asia and Pacific	−0.051	−0.160 to 0.058	0.360	
South Asia	0.015	−0.205 to 0.235	0.895	
Sub-Saharan Africa	−0.117	−0.284 to 0.050	0.170	
Middle East and North Africa	−0.005	−0.408 to 0.397	0.979	
Multiple regions	−0.007	−0.276 to 0.262	0.960	
Assessment method				
Diagnostic	Reference			
Screening	0.004	−0.061 to 0.070	0.894	0.563
Other	−0.097	−0.278 to 0.084	0.292	
Income level (country)				
High income	Reference			
Upper middle income	0.051	−0.065 to 0.167	0.389	0.213
Lower middle income	0.039	−0.127 to 0.205	0.645	
Low income	0.170	−0.022 to 0.362	0.082	
Mixed or unspecified	−0.058	−0.265 to 0.150	0.584	
Exclusion criteria				
Yes	Reference			
Partial	0.027	−0.034 to 0.087	0.383	0.291
No	−0.046	−0.130 to 0.037	0.277	
Unspecified	0.034	−0.050 to 0.119	0.427	
NOS score (study quality)				
Score: 6	Reference			
Score: 7	−0.017	−0.093 to 0.059	0.659	0.427
Score: 8	0.024	−0.056 to 0.103	0.559	
Age (mean or median)	0.003	−0.002 to 0.007	0.225	
Sex (by % male)	−0.003	−0.004 to −0.001	0.003	
Publication year	−0.001	−0.006 to 0.004	0.747	
Sample size	−0.0001	−0.0001 to 0.00002	0.157	

Nine predefined covariates were included in the model. None were found to significantly predict cognitive impairment in PWH, except for the percentage of male study participants. NOS, Newcastle-Ottawa Scale.

### Publication bias

The funnel plot, as well as the Egger's and Begg's tests, indicate that there is publication bias if the pooled estimate truly reflects the prevalence of cognitive impairment in PWH (Supplementary Fig. 33).

## Discussion

### Meta-analysis and meta-regression conclusions

Findings from this meta-analysis of 225 studies suggested an overall prevalence of cognitive impairment in PWH of 39.6%; this figure includes asymptomatic cases, which are highly debated to be of any clinical significance. Of all factors considered, only sex was found to be a significant predictor of cognitive impairment in the meta-regression analysis.

Estimates of cognitive impairment prevalence did not differ significantly between studies that used diagnostic methods and those that used screening methods. This was an unexpected finding as screening methods are less rigorous and do not employ a neuropsychological test battery. Studies using the HAND criteria (the so-called Frascati criteria) seem to have skewed the pooled prevalence estimate of diagnostic methods upwards. This is likely due to the 20% false-positive rate that the method is said to have [21]. Despite this understanding, the HAND criteria are the most frequently used method in research studies and thus the overall pooled 39.6% is arguably higher than the true value. However, we retained this method as it remains the only method used in many countries. Our estimate of 39.6% is marginally lower than the 42.6% reported by Wang *et al.*[14], whose stated focus was on HAND cases. The term HAND is misused in the article as studies using other criteria, such as the global deficit score, the multivariate normative comparison (MNC), and the novel multivariate method (NMM), were also considered. Furthermore, overestimation of the pooled prevalence made our assessment of publication bias likely inaccurate, since establishing the bias requires knowledge of the true prevalence of cognitive impairment in PWH.

Participants in many research studies are unselected for symptoms prior to the study and are also not checked for symptoms (that interfere with daily functioning) during the study. This is a significant issue as it prevents an accurate estimation of the proportion of asymptomatic cases across all studies. The use of the HAND criteria also complicate matters as self-reported cognitive difficulties are sufficient to differentiate asymptomatic from mild neurocognitive disorder (in patients who do not suffer from major depression) [6]. Thus, subjective and objective measures feed into the neuropsychological assessment [22]. This may skew upwards the number of individuals classified with mild neurocognitive disorder rather than an asymptomatic neurocognitive disorder (ANI). The number of cases with ANI was reported in 59% of studies using Frascati/HAND; the average proportion of ANI was 27%, which accounts for approximately two-thirds of individuals determined as cognitively impaired. Whether clinically diagnosing asymptomatic cases does more harm than good is debated. Cognitive impairment diagnosis can impact the psyche and indirectly negatively affect the individuals’ finances due to less employability and healthcare expenses. However, an early diagnosis does offer the opportunity of mitigating worse outcomes, using appropriate interventions. Nightingale *et al.*[8] recently published a new framework for cognitive impairment diagnosis in PWH, recommending that instead of labelling individuals with ‘asymptomatic neurocognitive impairment’, a preferable approach would be to describe these individuals as having ‘low performance on cognitive tests’ (p.4). An official cognitive impairment diagnosis necessitates consideration of an individual's clinical history, rather than just scoring below a cut-off in a neuropsychological test battery. This new framework offers a standardized approach that can be adopted in both research and clinical settings. However, the feasibility in low-resource settings must still be explored.

In this systematic review, studies undertaken only using screening methods reported a prevalence that was approximately 5% higher than those which used diagnostic methods. This relatively small difference could falsely suggest that screening methods do not lack in precision. As previously mentioned, diagnostic criteria, that have been labelled as such in this study, are predominantly used in research settings. Ferretti *et al.*[23] report a 7.5% cognitive impairment prevalence in a clinic in the United Kingdom, where ‘final diagnosis […] was based on clinician assessment’ after analysis of neuropsychological test results and consideration of potential confounders by ‘a multidisciplinary care-providing team’ (pp.e10–e12). This discrepancy between research and clinical settings arises because, in clinics, individuals who are asymptomatic are not examined for cognitive impairment, evaluation of confounding factors is more thorough, and medical history is considered.

To summarize, two issues remain: first, research criteria do not align with clinical cognitive impairment definitions, resulting in a high difference in prevalence estimates, and suggesting that methodologies currently used in research studies should be reconsidered (e.g. cohorts unselected for symptoms, relying only on self-reported symptoms, etc.); and second, a better understanding of the clinical cognitive impairment burden in resource-limited settings is needed. If screening methods are in use, one must reconsider the potential of overestimation the resulting added strain on PWH and already vulnerable health systems.

Significant subgroup differences were found for NOS score, exclusion criteria, country income level, and geographical region. As expected, high-income countries had the lowest prevalence rates in the univariate meta-analyses. The same is true for the geographical regions of North America and Europe and Central Asia. This was hypothesized as resource-rich countries have more comprehensive health services and capacities for long-term care of PWH [24]. However, a trend or pattern that encompasses all categories of country income level (ordinal variable) or geographical region (nominal variable), respectively, could not be found. Both these variables were categorized by the World Bank classification. Like geographical regions defined by the WHO or the United Nations, groupings largely depend on geographic proximity and reflect the organizations’ regional offices. An important difference lies in whether the Americas is considered a single region. Since the healthcare landscape and HIV prevalence rates differ between Northern and Southern America, the separation of the Americas into different regions seemed more appropriate in this study's context [1]. Saloner and Cysique [25] made a similar finding of similar prevalence rates among Western and non-Western cohorts. Apart from that, a high number of studies in meta-analyses may lead to the detection of clinically insignificant amounts of heterogeneity, in which case *Q*-test results must be considered with caution [20]. The NOS score (ordinal variable) shows no clear trend. Concerning study exclusion criteria (nominal variable), the subgroup without such criteria had a lower prevalence rate than studies with exclusion criteria. This is unexpected as it would be hypothesized that studies without exclusion of confounding comorbidities would have higher prevalence rates. To explore this, however, requires the proportion of individuals with comorbidities in these studies without exclusion criteria, which is rarely reported [26].

Sex was the only covariate found to be a significant predictor at the 0.05 significance level in the meta-regression model with men being less vulnerable to cognitive impairment than women. Sundermann *et al.*'s study [27] also concluded that cognitive impairment was more prevalent in women because of syndemics, including biological and psychosocial factors. Rubin *et al.*[28] found that men and women had varying cognitive profiles. Women experienced weaknesses in motor function, learning and memory; men showed ‘relative strengths in attention and processing speed’ (p.12). However, the skewed sex prevalence in their cohort limits the statistical power and representativeness for the female cohort sample. In other words, mixed cohorts may mask sex-specific cognitive impairment outcomes. Another aspect to consider is the lack of female control participants in studies, especially when using MNC and NMM.

When considering the other covariates, it was surprising that age, ART coverage and publication year were not significantly correlated with cognitive impairment prevalence, either by univariate analyses or after adjustment under the meta-regression model. Findings from studies by Bhaskaran *et al.*[29], Hardy and Vance [30] and Valcour *et al.*[31] suggest that age is a risk factor for cognitive impairment in PWH. A reason why no correlation was identified is that only summary measures of cohort age (e.g. mean and median), which mask the actual age ranges of participants, were considered in the meta-regression. Despite ART being a main protective factor for HIV-related cognitive impairment, there is a lack of correlation of cognitive impairment with ART coverage or publication year. Surprisingly, this is consistent with results from other studies [32,33]. Multiple factors could explain this: ART toxicity, for example, with efavirenz, can mitigate the cognitive benefits of ART [5,34]. Furthermore, whether an individual is reportedly on ART does not give a suggestion of the duration of nor adherence to the treatment, with the latter rarely reported in studies. It also does not indicate the resulting viral loads and CD4^+^ cell counts. In addition, even though severe forms of cognitive impairment have decreased since the pre-ART era, mild impairments are diagnosed earlier and more frequently [3,6]. All these points could falsely suggest that there were no changes in cognitive impairment burden pre and post-ART.

### Study strengths and limitations

The comprehensiveness of the systematic literature search is an aspect lacking in previous studies. We took a broad approach to the search terms to capture all relevant studies and searched four databases. By following PRISMA guidelines and reporting the steps taken during the literature search, the study is reproducible. Furthermore, our conclusions are evidence-based, with regard to 225 studies. This sizeable set of studies was used to explore nine covariates in a meta-analysis and meta-regression.

**Fig. 3:**
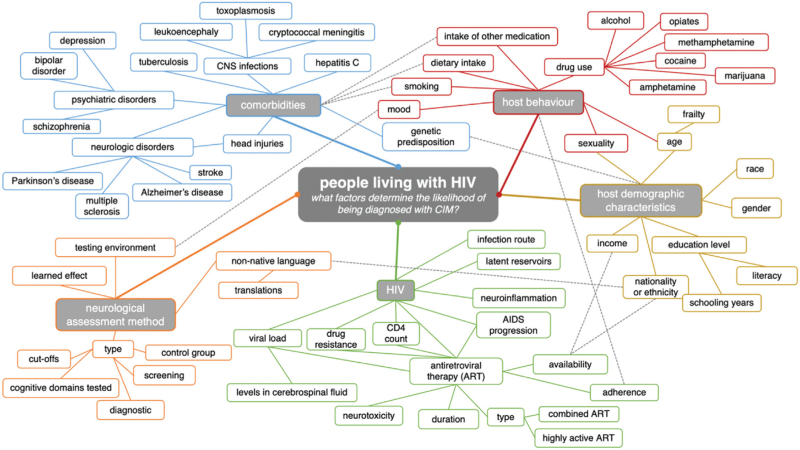
Web of factors determining cognitive impairment in an HIV-positive individual.

Limitations relate to our eligibility criteria, restricting the applicability of the results from this meta-analysis. Moreover, although there are subpopulations within the overall population of PWH with higher cognitive impairment risk, we did not include studies in the meta-analysis that focussed solely on these subpopulations, although they may have been included in the sample. Our results are also limited by a lack of studies from some geographical regions, notably the Middle East, North Africa, and South Asia, which may limit our findings to these regions. An anticipated limitation was the inclusion of the HAND criteria, as well as screening methods, which have skewed the pooled prevalence upwards; this was not avoided, since otherwise, a lack of studies would have prevented prevalence estimates on a global scale. Two variables chosen for data extraction, age and ART coverage, are reported in varying formats and with different definitions between studies, complicating the assessment of these factors. In addition, we were unable to assess the impact of either the (present or nadir) CD4^+^ cell count or duration on ART due to lack of availability in many studies as well as differences in the format for presentation when available. Years of education and viral loads were rarely reported in the published articles. Finally, many factors may confound the assessment of cognitive impairment risk (see Fig. 3, which builds on the simpler web diagram of Tedaldi *et al.*[35]). In a single study, authors generally do not provide data on all factors in the network diagram. Therefore, adjusting for all confounders is not feasible.

Various determinants influence the risk for cognitive impairment in people with HIV

### Future implications and considerations

Our findings may provide the foundation for further analyses of cognitive impairment burden in the population of PWH. However, some areas of research were beyond the scope of this review, such as cognitive impairment prevalence in specific vulnerable subpopulations. Further analysis can use patient registry data as a separate source, bringing in more data from clinical settings. In addition, the heterogeneity among studies calls for further research into the specificity and sensitivity of neuropsychological assessment methods. Methods must be validated in different cultural and geographical settings, especially for resource-limited settings, where screening approaches are used for clinical diagnosis. Furthermore, the considerable between-study heterogeneity is also in part due to the wide range of diagnostic methods and criteria available. A more standardized approach is necessary. Due to the over diagnosis when using the HAND criteria, the general advice would be to use alternative approaches that are more sensitive in identifying clinically true cases of cognitive impairment. Newly described methods, like MNC and NMM, incorporate well-matched controls into the diagnostic procedure. A further option is to follow the suggested framework by Nightingale *et al.*[8], which addresses the contemporary challenges in cognitive impairment diagnosis among PWH.

### Conclusion

The prolonged lifespan in the post-ART era exposes PWH to cognitive ageing, besides the added risk for neurocognitive disorders from direct associations of HIV pathogenesis with cognitive impairment progression. Our meta-analysis establishes a pooled cognitive impairment prevalence of 39.6%. This is predominantly based on studies using research definitions. Future studies must close the research gap of studies with stronger clinical perspectives. Only 13.3% of between-study variation was explained through the meta-regression model, with substantial heterogeneity remaining. Of the covariates explored, only sex was a significant predictor. More than 70% of countries globally had no studies reporting on cognitive impairment in PWH. Findings from the systematic review and meta-analysis highlight the need for more explorative studies from low-resource settings and a standardized approach to assessing cognitive impairment across research and clinical settings.

## Acknowledgements

Authors’ contributions: C.A.S. and A.W. conceived the study idea and, with L.D.K., designed the study and identified search criteria. L.D.K. conducted the review with support from C.A.S., and generated the initial manuscript draft. C.A.S. and A.W. provided critical revisions of the article. All authors have approved the final version for publication and have agreed to be accountable for the work.

### Conflicts of interest

C.A.S. reports receipt of consultancy fees and honorarium for participation in advisory boards and for preparation of educational materials. A.W. reports receipt of honoraria for participation in advisory boards and educational talks.

## Supplementary Material

Supplemental Digital Content
