# Supplementary material for: The global burden of cognitive impairment in people with HIV
Source: AIDS. 2022 Sep 15;37(1):61–70. doi: 10.1097/QAD.0000000000003379 (PMC9794154; doi:10.1097/QAD.0000000000003379)

Supplementary Table 1: Raw data sheet.

Author	Year	Country	Criteria (Cut-Off)	Sample Size	Cognitively Impaired (ANI, MND, HAD in %)	Mean Age (SD) or Median (IQR), Range	ART Coverage (%)	Male (%)	Exclusion Criteria for Study Participants	Recruitment Year(s) / Time of Testing	Cohort / Place of Recruitment	NOS Score
**Arentoft et al.**	**2015**	USA	Frascati	134	68 (56, 5, 7)	47.6 (8.1)	90.6	70	yes	.	community outreach in NYC (East Harlem), Icahn School of Medicine at Mount Sinai	7
**Agarwal et al.**	**2020**	India	MoCA (26)	160	52.5	44 (10)	50	75.6	yes	.	Chandra Laxmi Hospital, Vaishali, Ghaziabad	7
**Akena et al.**	**2010**	Uganda	MMSE	64	70.3	32 (6.5)	.	21.9	no, only depression included	2007	mental health units of Mulago general hospital and Butabika psychiatric hospital	6
**Akolo et al.**	**2014**	Nigeria	GDS	133	30.8	33.6 (7.3)	0	42.1	yes	.	National Hospital (NH) & University of Abuja Teaching Hospital (UATH)	8
**Alford et al.**	**2019**	UK	Frascati (uncategorised)	52	31	55'median', 36-84	98	79	no, BUT score is adjusted	2016-2018	The Orange Clinic, Brighton (memory clinic)	6
**Animut et al.**	**2019**	Ethiopia	IHDS (9.5)	684	67.1	38.8 (8.8)	100	44	only neurological disorders & psychiatric disorders excluded	2017	Gamo Gofa zone	8
**Araya et al.**	**2020**	Ethiopia	IHDS (9.5)	581	35.6	38 (9.8)	100	38.7	no	2018	four federal hospitals in Addis Ababa	7
**Arenas-Pinto et al.**	**2014**	UK	Frascati (uncategorised)	548	52	44 (9)	100	76	unspecified	2008-2010	Protease Inhibitor monotherapy Versus On-going Triple-therapy (PIVOT) trial	8
**Asiedu, Kretchy & Asampong**	**2020**	Ghana	IHDS	104	48	37.2 (10.1)	100	21.2	only psychiatric disorders & head injuries excluded	2017	Ridge Hospital in Accra	7
**Atashili et al.**	**2013**	Cameroon	IHDS (10)	400	85	41 (34-47)	100	26	unspecified	2010	Bamenda Regional Hospital AIDS Treatment Centre	7
**Atkins et al.**	**2010**	Canada	clinical NP ratings	357	48.5	41.5, 19-64	70	100	unspecified	.	St. Michael's Hospital in Toronto	8
**Aung et al.**	**2021**	Australia	GDS	254	42	49.4 (9.5)	91.7	.	only alcohol/substance intoxication at time of assessment excluded	2011-2012	primary care clinic in Sydney	8
**Awori et al.**	**2018**	Kenya	MoCA (26)	215	69	44.5, 18-65	100	47.2	only CNS abnormalities excluded	2015-2016	Aga Khan University Hospital	8
**Bai et al.**	**2017**	Italy	Frascati	155	32.3 (25.8, 6.5, 0)	39 (31-46)	0	92	yes	.	.	8
**Banerjee, McIntosh & Ironson**	**2019**	USA	HDS (10)	209	14.8	37.7 (8.7)	49.3	71.8	yes	1997-2000	physician's offices, specialty clinics, service organizations, and hospitals in Miami, Florida	8
**Barber et al.**	**2017**	UK	IHDS (10)	144	21.5	41 (37-45)	.	100	yes	.	MSM Neurocog Study	6

**Becker et al.**	**2004**	USA	clinical NP ratings	290	20.2	39.3 (8.2)	17	83.9	no	.	Allegheny County Neuropsychiatric Survey (ACNS)	8
**Belete, Medfu & Yemiyamrew**	**2017**	Ethiopia	IHDS (9.5)	234	33.3	38.3 (9.9)	88	35	yes	2016	Ayder Comprehensive Specialized Hospital	8
**Bharti et al.**	**2021**	Nigeria	GDS	174	27.6	34.3 (7.6)	0	39.7	unspecified, 36% ASYMPTOMATIC MALARIA	2011-2012	neuroAIDS study in Abuja, Nigeria	8
**Bloch et al.**	**2016**	Australia	Frascati	254	30.7 (15, 12.6, 3.1)	48.5 (15.1)	91.7	99.6	no	2011-2012	Holdsworth House Medical Practice, Sydney	8
**Boccellari et al.**	**1993**	USA	Gisslen (uncategorised)	55	22	39.6 (5.5)	12.7	100	yes	1989	San Francisco General Hospital	7
**Bornstein et al.**	**1993**	USA	(1.5 SD in at least 6 out of 15 measures)	233	12.8	34.4 (7.1)	.	100	only neurological, head injuries & history of drug use excluded	.	AIDS Clinical Trials Unit and local HIV-related community-based support groups	7
**Bourgeois et al.**	**2020**	USA	MoCA (26)	359	33.7	57.4 (5.9)	98	85	unspecified	2012-2014	two San Francisco HIV clinics	8
**Braganca & Palha**	**2011**	Portugal	GDS	130	51	39.3 (6.1)	100	63.1	yes	2008	Infectious Diseases Service (IDS) of Hospital of S. João	7
**Brito-Marques et al.**	**2020**	Brazil	MoCA (23)	133	83.5	44.7 (8.8)	.	57.9	yes	2016-2017	Hospital Universitário Oswaldo Cruz (HUOC), Universidade de Pernambuco (UPE), in Recife	7
**Brouillette et al.**	**2015**	USA	Frascati (HAD)	200	33	43 (11)	100	72	yes	.	outpatient Infectious Disease Clinic at Washington University in St. Louis (WUSTL)	8
**Brouillette et al.**	**2021**	Canada	GDS	263	52.5	54.4 (8.0)	.	84	yes	2014-2017	Positive Brain Health Now Canadian cohort	8
**Bryant et al.**	**2015**	USA	Frascati (uncategorised)	120	58.1	45.2 (9.5)	82.4	63.6	no	.	The Miriam Hospital Immunology Center	6
**Bunupuradah et al.**	**2012**	Thailand	Frascati (uncategorised)	93	31.2	36.9 (32.8-40.5)	100	60.2	only CNS infections excluded	2008-2009	HIV STAR study	7
**Carvalhal et al.**	**2016**	Canada	GDS	417	60	46.8 (9.7)	100	81	no	.	Ontario HIV Treatment Cohort Study (OCS)	8
**Casado et al.**	**2014**	Spain	Frascati (uncategorised)	229	13	44.6 (38-51)	100	77	only psychiatric disorders & CNS infections excluded	2011-2012	.	7
**Chalermchai et al.**	**2013**	Thailand	clinical NP ratings	75	51	34 (7)	0	44	yes	2008-2012	SEARCH 007 & 011, Bangkok	8
**Chan et al.**	**2012**	Singapore	Frascati	132	22.7 (15.9, 5.3, 1.5)	45.9 (10.1)	.	86.4	only neurological & psychiatric disorders excluded	2010	Communicable Disease Centre of Singapore	7
**Chan et al.**	**2021**	Thailand	Frascati (uncategorised)	67	30	28'median'	0	96	unspecified	.	RV254 Thai AHI cohort	6
**Chan et al.**	**2019**	China	IHDS (10)	98	39	31 (26-43)	0	94	only neurological & psychiatric disorders excluded	2013-2015	AIDS Clinical Service, Queen Elizabeth Hospital, Hong Kong	7
**Chang et al.**	**2011**	USA	Frascati	69	42 (18.8, 21.7, 1.4)	47.4 (1.2)	80	91	yes	.	.	6
**Ciccarelli et al.**	**2011**	Italy	Frascati	146	47.2 (35.6, 11.6, 0)	46.6 (20.4-54.8)	88.4	58	yes	2008-2010	.	7
**Ciccarelli et al.**	**2013**	Italy	Frascati	101	49.5 (49.5, 0, 0)	47 (42-52)	100	66	yes	2008-2010	.	7

**Ciccarelli et al.**	**2019**	Italy	Gisslen (uncategorised)	386	12	46 (40-52)	96.1	79.3	yes, 19.4% HCV-COINFECTION	.	Agostino Gemelli University Hospital, Rome and S. Caterina Novella Hospital, Galatina	8
**Cook et al.**	**2014**	India	Frascati (uncategorised)	80	57	38.2 (8.4)	100	70	only psychiatric disorders & substance use excluded	2009-2010	PGIMER Immunodeficiency Clinic in Chandigarh, India	7
**Cook et al.**	**2016**	India	IHDS (10)	75	36	29 (6)	0	62.7	only CNS infections, head injuries & substance use excluded	.	Postgraduate Institute of Medical Education & Research, Chandigarh	7
**Cross et al.**	**2013**	USA	IHDS (10)	507	41	42 (33-49)	75	65.1	no	2008	.	6
**Crum-Cianflone et al.**	**2013**	USA	GDS	200	19	36.4 (28.1-43.6)	70.5	95.5	only acute medical events excluded	.	.	6
**Cruz & Ramos**	**2015**	Brazil	MMSE (23)	142	28.1	60-81	82.3	56.3	unspecified	2008-2012	Reference and Treatment Center for STD/AIDS in the city of São Paulo	6
**Cysique et al.**	**2010**	China	GDS	192	42.7	40.2 (6.3)	60.9	61	unspecified	.	Anhui province, local hospital in Fuyang City	8
**Dampier et al.**	**2017**	USA	GDS	112	59.3	51 (46-56)	100	64.3	only alcohol/substance intoxication at time of assessment excluded	.	CARES cohort	8
**Dang et al.**	**2015**	China	IHDS	230	37.5	18-65	.	.	yes	2011-2012	Guangxi Zhuang autonomous region of Southern China	6
**Darling et al.**	**2021**	Switzerland	Frascati	981	39.8 (25.4, 0.8, 0.6)	54.5 (7.5)	97.8	79.7	no	2013-2016	NAMACO study	8
**Davies et al.**	**2019**	UK	MNC	78	28	46.9 (12.4)	100	100	yes	.	.	6
**Day et al.**	**2016**	China	GDS	308	26.6	36 (32-40)	37	65	unspecified, 91% HCV COINFECTION	.	Anhui and Yunnan, China	8
**de Almeida et al.**	**2017**	Brazil	Frascati	60	60 (48.3, 3.3, 8.3)	42.5 (9.1)	78	50	yes, BUT 20% with HCV COINFECTION	.	Hospital de Clinicas UFPR (HC-UFPR), Curitiba	8
**de Ronchi, Faranca & Berardi**	**2002**	Italy	Frascati (HAD)	182	20.3	.	98.9	79.1	only neurological, psychiatric disorders & CNS infections excluded	1994-1997	University of Bologna	7
**Deiss et al.**	**2019**	USA	GDS	189	18.5	36 (28-44)	65.6	100	no	2009-2011	.	7
**Derry et al.**	**2020**	USA	MoCA (23)	162	35	61.2 (5.8)	.	67	unspecified	.	Weill Cornell Clinical and Translational Science Center	6
**Ding et al.**	**2017**	China	IHDS (10)	345	46.7	52.7 (9.5)	87	78	unspecified	2014-2015	Taizhou Prefecture of Zhejiang Province	7
**Donne et al.**	**2020**	Italy	Frascati	85	7 (7, 0, 0)	54 (48-60)	.	78	yes	2018-2019	Infectious Diseases Institute of Policlinico Gemelli Foundation of Rome	7
**Duarte et al.**	**2020**	Brazil	IHDS (10)	148	69.6	43'median'	.	38.5	unspecified	2019-2020	Hospital Nereu Ramos (HNR)	6
**Dufouil et al.**	**2015**	France	Frascati	400	58.5 (20.8, 31, 6.8)	47.3 (10.2)	95	79.2	unspecified	2007-2009	(ANRS) CO3 Aquitaine cohort	8
**Dufour et al.**	**2013**	USA	GDS	335	22.1	47.7 (10.5)	82.2	74	yes	2007-2011	HIV Neurobehavioral Research Center (HNRC)	8
**Dwyer et al.**	**2014**	China	Frascati (uncategorised)	50	69	35 (7.3)	70	84	unspecified	.	Ditan Hospital	6

**Elham et al.**	**2020**	Iran	Frascati	93	50.6 (23.7, 18.3, 8.6)	36.6 (9)	92.5	60	only psychiatric disorders & CNS infections excluded	2016-2017	VCT center, Tehran	8
**Ene et al.**	**2016**	Romania	GDS	194	36.5	24 (1.5)	91.7	48.4	yes	2014	.	8
**Erlandson et al.**	**2019**	USA	Frascati (uncategorised)	987	17	51'median'	.	81	unspecified	2013-2014	HAILO study	6
**Estiasari et al.**	**2015**	Indonesia	Frascati (uncategorised)	82	51	31 (19-48)	0	68	only neurological, psychiatric disorders, head injuries & CNS infections excluded	2013-2014	Cipto Mangunkusumo Hospital, Jakarta	7
**Fabbiani et al.**	**2018**	Italy	Frascati	266	16.2 (16.2, 0, 0)	44 (36-50)	100	79.7	only psychiatric disorders & substance use excluded	.	ATLAS-M trial	7
**Fabbiani et al.**	**2017**	Italy	Frascati (uncategorised)	54	13	50'median', 27-60	100	85.2	only CNS infections & substance use excluded	.	.	6
**Fabbiani et al.**	**2019**	Italy	T-Score (40)	146	24	50 (46-54)	92.5	76	yes, ALL COINFECTED WITH HCV	2009-2018	Rome, Monza and Siena	7
**Failde-Garrido et al.**	**2008**	Spain	Frascati (HAD)	88	52.3	33.8 (5.2)	83	64.8	yes	.	hospitals in Ourense	7
**Fazeli, Woods & Vance**	**2019**	USA	clinical NP ratings	174	53	51.3 (7)	91	62	only neurological & psychiatric disorders excluded	.	University HIV/AIDS Clinic	8
**Ferrando et al.**	**2003**	USA	Frascati (uncategorised)	141	62	40 (8)	.	100	only history & current substance use excluded	1995-1997	.	6
**Fialho et al.**	**2013**	Portugal	HDS (10)	103	57.3	43.9 (12.4)	100	0	only neurological, psychiatric disorders & current substance use excluded	2010	Infectious Diseases Service (IDS) of the Portuguese National Health System Hospital (Lisbon)	7
**Filho & de Melo**	**2012**	Brazil	MMSE (24)	52	36.5	57.6 (6.2)	94.2	55.8	only neurological & psychiatric disorders excluded	2008	Correa Picanço Hospital and the University Hospital of the Universidade Federal de Pernambuco (UFPE)	7
**Fitri, Rambe & Fitri**	**2018**	Indonesia	MoCA (26)	85	75.3	38.5 (9.8)	.	61.2	only neurological, psychiatric disorders & CNS infections excluded	2017	Adam Malik General Hospital Medan North Sumatera Indonesia	6
**Flatt et al.**	**2021**	Tanzania	Frascati	253	47.1 (25.3, 18.2, 3.6)	57'median'	95.5	27.7	no	2016	Mawenzi Regional Referral Hospital (MRRH) HIV Care and Treatment Centre (CTC)	8
**Foca et al.**	**2016**	Italy	Frascati	206	47.1 (30.6, 15, 1.5)	40.2 (10.4)	.	85	only neurological, psychiatric disorders & CNS infections excluded	2009-2013	University Department of Infectious and Tropical Diseases of University of Brescia and Spedali Civili General Hospital (Brescia, Italy)	8
**Foley et al.**	**2013**	USA	GDS	79	21.5	21-79	.	78.5	only CNS infections & brain injuries excluded	.	local hospitals and community agencies in the Los Angeles area	7

**Ganasen et al.**	**2008**	South Africa	HDS (10)	474	17.1	34.3 (7.9)	.	26	unspecified	.	primary healthcare HIV clinics, in the Western Cape of South Africa	6
**Gandhi et al.**	**2011**	USA	Frascati	114	86 (32.5, 19.3, 34.2)	46.8 (6.4)	.	66.7	only neurological, psychiatric disorders & CNS infections excluded	2007-2010	General Clinical Research Clinic at Johns Hopkins Hospital in Baltimore	7
**Garvey, Surendrakumar & Winston**	**2011**	UK	Frascati (uncategorised)	101	19	53 (43-62)	100	88	only neurological & substance use history excluded	.	.	7
**Gascon et al.**	**2018**	Brazil	Frascati	412	73.6 (50.9, 16.2, 6.3)	45.3 (10.7)	100	68.3	yes	2013-2015	Institute of Infectious Diseases Emilio Ribas (IIER), São Paulo	7
**Gibbie et al.**	**2006**	Australia	HDS (10)	129	7	44.7	93	95	unspecified	.	Melbourne	6
**Gomez et al.**	**2019**	Canada	Frascati	381	21.2 (8.1, 11, 2.1)	47.3 (11.1)	94	88	only neurological, psychiatric disorders, CNS infections & head injuries excluded	.	Southern Alberta HIV Clinic (SAC) in Calgary, Alberta	8
**Goodkin et al.**	**2014**	South Africa	Frascati (uncategorised)	70	43	31.5 (8)	0	18.6	unspecified	.	.	6
**Gott et al.**	**2017**	Australia	GDS	96	55.2	56.1 (7.9)	100	97.9	only neurological, psychiatric disorders & substance use excluded	2009-2011	St Vincent's Hospital	8
**Grauer et al.**	**2015**	Germany	Frascati	86	87.2 (29.1, 43, 15.1)	49'median', 19-72	89	82.6	unspecified	2012-2014	University Hospital Muenster	7
**Greene et al.**	**2015**	USA	MoCA (26)	155	46.5	57 (54-62)	100	93.6	no	.	University of California San Francisco SCOPE cohort	7
**Grima et al.**	**2012**	Italy	Frascati	116	46.6 (46.6, 0, 0)	44 (37-49)	97.4	78.5	yes	2010-2011	.	7
**Groff et al.**	**2020**	USA	Frascati	77	37.7 (26, 9.1, 2.6)	47.5	100	59.7	yes	.	.	6
**Grund et al.**	**2013**	Australia, USA, Brazil, Thailand	Frascati (HAD)	258	13.6	40 (35-45)	96.1	57.8	unspecified	2005-2006	SMART study	7
**Gupta & Venugopal**	**2020**	India	Frascati (uncategorised)	384	48	38.3 (9.2)	100	62.5	yes	.	.	7
**Gupta et al.**	**2007**	India	(below 15th percentile in at least 2 domains)	119	60.5	29.9 (5.6)	0	43.7	only neurological & CNS infections history excluded	2003-2004	National Institute of Mental Health and Neuro Sciences (NIMHANS), Bangalore	6
**Haddow et al.**	**2018**	UK, Denmark, Belarus, Italy	Frascati	448	25.8 (20, 4.9, 0.9)	45.8 (9.6)	89.1	84.4	no	2011-2013	MULTICENTER study: CIPHER study	8
**Halman et al.**	**2014**	Canada	Frascati (uncategorised)	83	48.2	49.2 (10.5)	74.7	80.7	no	2008	Casey House	7
**Hanna et al.**	**2020**	USA	Frascati	108	37 (21.3, 9.2, 6.5)	26-72	.	58	yes	.	.	6
**Heaton et al.**	**2008**	China	GDS	201	35.5	40.9 (6.4)	64	60.7	yes	2005	Fuyang, Anhui	8

**Hestad et al.**	**2019**	Zambia	GDS	275	36.7	41.1 (8.8)	100	38.6	only neurological, psychiatric disorders & substance use excluded	.	Lusaka	8
**Hiransuthikul et al.**	**2019**	Thailand	MoCA (25)	340	59.4	55 (52-59)	.	61.5	unspecified	2015-2017	HIV-NAT 006 long-term cohort	6
**Holguin et al.**	**2011**	Zambia	IHDS (10)	54	22	34 (0.8)	0	35	only brain injuries excluded	2008	Lusaka	7
**Imai et al.**	**2020**	Japan	Frascati	444	26.1 (16, 9.7, 0.4)	45 (40-53)	.	95	yes, BUT 13% with hemophilia	2016-2018	AIDS Clinical Center in National Center for Global Health and Medicine (ACC)	8
**Imam**	**2007**	Nigeria	MMSE (25)	202	12.3	35.7 (12-60)	59.9	47.6	no	2003	State House Clinic in Abuja	7
**Invernizzi et al.**	**2018**	Italy	MoCA (26)	69	49.2	53 (7.3)	100	69.6	no	2015-2016	Luigi Sacco Hospital	6
**Janssen et al.**	**2015**	Netherlands	Frascati	95	41.1 (35.8, 5.3, 0)	48.2 (10.1)	100	87.4	yes	2012-2014	Art-NeCo study	7
**Janssen et al.**	**1989**	USA	Frascati (HAD)	100	20	35.1 (4)	.	100	yes	1978-1980	San Francisco City Clinic	6
**Jevtovic et al.**	**2009**	Serbia	HDS	96	41.7	44 (12.1)	100	80.2	unspecified	.	Institute of Infectious and Tropical Diseases in Belgrade	6
**Joska et al.**	**2016**	South Africa, USA	Frascati	156	74 (26.2, 29.4, 12.2)	40'median'	100	37.2	unspecified	.	Cape Town and Baltimore	8
**Joska et al.**	**2011**	South Africa	Frascati	170	76.5 (8.8, 42.4, 25.3)	29.5 (3.6)	0	26	only mental health problems, substance use & head injuries excluded	2008-2009	three primary health care centers in Cape Town	8
**Joska et al.**	**2019**	South Africa	CSID	55	18.18	.	86.1	29.1	unspecified	.	Eastern Cape in South Africa	6
**Jumare et al.**	**2020**	Nigeria	Frascati	190	24.2 (16.8, 7.4, 0)	33 (29-40)	0	34.7	yes	2011-2014	Abuja, Nigeria	7
**Jumare et al.**	**2019**	China, India, Nigeria	GDS	761	27.7	35 (9)	.	58.2	unspecified	.	.	6
**Kabuba et al.**	**2017**	Zambia	GDS	266	34.6	40.7 (8.7)	100	40	only neurological disorders & substance use excluded		Chilenje, Chipata, Kabwata, Kalingalinga, Matero Main, and Matero Referal clinics	8
**Kalayjian et al.**	**2019**	USA	GDS	225	22.7	.	100	90.9	only neurological disorders excluded	.	AIDS Clinical Trials Group Study A5303	7
**Kalayjian et al.**	**2014**	USA	BNCS	1972	24	40 (33-47)	89	82	unspecified	.	ALLRT study	7
**Kallianpur et al.**	**2016**	USA	GDS	1261	36.2	43 (8.6)	70.3	77	yes	2003-2007	CHARTER study	8
**Kalungwana et al.**	**2014**	Zambia	MMSE (22)	58	50	36.8 (28-47)	36	53	unspecified	2011-2013	University Teaching Hospital in Lusaka	6
**Kamal et al.**	**2017**	Switzerland	Frascati	59	27.1 (15.2, 6.8, 5.1)	53 (47-58)	100	66	no, BUT subsequently excluded	.	Swiss HIV Cohort Study (SHCS)	7
**Kamat et al.**	**2017**	India	GDS	69	33	37.4 (8.1)	61	66.7	unspecified	2010-2013	YRG CARE in Chennai	7
**Kamminga et al.**	**2017**	Australia	GDS	55	47.2	49.3 (8.9)	87	100	only neurological, psychiatric disorders & current substance use excluded	2011-2012	Sydney	8
**Karlsen, Froland & Reinvang**	**1994**	Norway	T-Score (40)	52	26.9	36 (8.4)	.	88.5	only drug use excluded	1986-1990	National Hospital (Rikshospitalet)	6
**Kelly et al.**	**2014**	Malawi	Frascati	106	70 (55, 12, 3)	39'median', 18-71	100	27	only neurological disorders excluded	2011-2012	Queen Elizabeth Central Hospital (QECH), Blantyre	8
**Kemp et al.**	**2021**	UK	NMM	140	40.7	52.2 (9.7)	96	83	only neurological disorders excluded	.	HIV-POGO study	8

**Kim et al.**	**2016**	South Korea	Frascati	194	26.3 (13.9, 12.4, 0)	45.1 (11)	86.6	93.8	yes	.	Seoul	7
**Kinai et al.**	**2017**	Japan	Frascati	728	25.3 (13.5, 10.6, 1.2)	45.6 (10.6)	97	95	yes	2014-2016	.	6
**Klusman et al.**	**1991**	USA	Frascati (HAD)	103	37.9	28.7 (7.1)	.	100	only neurological, psychiatric disorders & current substance use excluded	1986-1989	.	6
**Korten et al.**	**2021**	Turkey	Frascati	162	45.7 (37.7, 7.4, 0.6)	43.5'median'	100	94	only neurological, psychiatric disorders & current substance use excluded	2018	Istanbul	8
**Ku et al.**	**2014**	South Korea	Frascati	194	26.3 (13.9, 12.4, 0)	45.1 (21-72)	89.7	93.8	yes	2012	Korean NeuroAIDS Project, Seoul	8
**Kumar et al.**	**2019**	India	MMSE	200	21	.	.	65	unspecified	2015-2016	KGMU, Lucknow	6
**Kupprat et al.**	**2017**	USA	MMSE	169	23.7	55.8 (4.6)	.	100	only brain injuries excluded	2010-2011	Project Gold	7
**Lawler et al.**	**2011**	Botswana	Frascati (uncategorised)	60	36.7	37.5 (6.2)	100	48.3	yes	2009	Infectious Disease Care Clinic (IDCC) at PMH in Gaborone	7
**Lawler et al.**	**2010**	Botswana	IHDS (9.5)	120	38	37.5 (6.5)	97.5	50	yes	2008	Infectious Disease Care Clinic at Princess Marina Hospital	7
**Libertone et al.**	**2014**	Italy	Frascati	556	31.7 (16.4, 13.1, 2.2)	.	100	78	unspecified	.	.	6
**Lu et al.**	**2014**	Australia	Frascati	55	49.1 (29.1, 12.7, 7.3)	57.7 (8.3)	.	96.4	yes	2011-2012	HIV and Ageing Observational Cohort Study and the Neuro-HAART HIV Trials	7
**Madan, Singh & Golechha**	**1997**	India	MMSE (20)	172	10.5	20-50	.	.	unspecified	1994-1996	.	6
**Maj et al.**	**1994**	Germany, Brazil, Zaire, Kenya, Thailand	(2 SD in at least 3 out of 10 measures)	602	13	.	.	.	unspecified	.	WHO Neuropsychiatrie AIDS Study	6
**Makinson et al.**	**2020**	France	Frascati	200	35.5 (24.5, 10.5, 0.5)	62.7 (55.1-71)	.	85	only psychiatric disorders & CNS infections excluded	.	The ANRS EP58 HAND 55–70 Study	8
**Marin-Webb et al.**	**2016**	Germany	Frascati	480	43 (20, 17, 6)	43 (35-51)	89	98.3	yes	2012-2014	.	7
**Matchanova et al.**	**2020**	USA	GDS	142	59.6	57.1 (6.2)	88.7	85.2	yes	.	greater San Diego county	8
**Mayeux et al.**	**1993**	USA	Frascati (uncategorised)	111	44.1	41.2 (7.9)	.	100	unspecified	.	.	6
**McCutchan et al.**	**2007**	USA	Frascati (uncategorised)	286	27	40'median'	93	90	no	1999	AIDS Clinical Trials Group (ACTG) 362, not ALLRT according to Smurzynski et al. (2008)	7
**McNamara et al.**	**2017**	Ireland	BNCS	604	51.5	40.9 (10.2)	84.3	78.8	yes	2010-2013	St. James's Hospital, Dublin (SJH)	8
**Metral et al.**	**2020**	Switzerland	Frascati	981	26.8 (25.4, 0.8, 0.6)	54.5 (7.5)	.	79.7	no, BUT score is adjusted	2013-2016	NAMACO study	7
**Milanini et al.**	**2017**	USA	Frascati	74	52.7 (21.6, 31.1, 0)	67 (61-83)	92	95	yes	.	UCSF HIV Over 60 Cohort Study	8
**Milanini et al.**	**2020**	Kenya, Tanzania, Uganda, Nigeria	Frascati (uncategorised)	2472	38	39.7 (10.7)	68	41	unspecified	.	African Cohort Study (AFRICOS)	8

**Mogambery et al.**	**2017**	South Africa	IHDS (10)	146	53	35 (18-58)	0	45.9	only psychiatric disorders & CNS infections excluded	2014-2015	peri-urban HIV clinic in KwaZulu-Natal	7
**Mohamed, Oduor & Kinyanjui**	**2020**	Kenya	MoCA	360	81.1	40.2 (11.5)	100	35	yes	.	Moi Teaching and Referral Hospital (MTRH)	8
**Molinaro et al.**	**2020**	Uganda	IHDS	399	59	35 (8)	0	53	only neurological disorders excluded	2013-2015	Rakai Community Cohort Study	8
**Moore et al.**	**2012**	USA	GDS	200	19	36.4 (28.1-43.6)	64	95.5	unspecified	.	.	7
**Mugendi et al.**	**2019**	Kenya	IHDS (10)	345	12.5	42 (9.5)	100	58.6	yes	2015	HIV clinic of the Kenyatta National Hospital in Nairobi	8
**Mukherjee et al.**	**2018**	Malaysia	MoCA (26)	342	22.8	44.7 (10.2)	100	82.2	no	2014-2016	University of Malaya Medical Center (UMMC) in Kuala Lumpur	7
**Munoz-Moreno et al.**	**2010**	Spain	Frascati	83	42.2 (19.3, 16.9, 6)	44 (37-51)	.	73.5	yes	2006-2008	HIV Unit of the Germans Trias i Pujol University Hospital	7
**Munoz-Moreno et al.**	**2013**	Spain	Frascati	106	48 (33, 15, 0)	44 (39-48)	86	87	no	2008-2011	7 hospitals in Barcelona	7
**Munoz-Moreno et al.**	**2008**	Spain	Frascati (uncategorised)	64	60.9	.	73.4	70.3	only psychiatric disorders & subtance use excluded	.	HIV outpatient clinic of the hospital, which is located on the outskirts of Barcelona	6
**Nakku, Kinyada & Hoskins**	**2013**	Uganda	IHDS (10)	618	64.4	35	64.6	27.3	no	.	semi-urban district of Entebbe	7
**Namagga et al.**	**2019**	Uganda	IHDS (10)	393	58.2	37.9 (8.6)	100	26.7	yes	2017	Mbarara and Rukungiri districts in rural Southwestern Uganda	8
**Naveed et al.**	**2021**	USA	T-Score (40)	877	39.1	47.1 (10.7)	81.1	80.5	no	2000-2017	National NeuroAIDS Tissue Consortium (NNTC)	7
**Nichols et al.**	**2013**	USA	GDS	215	64.7	20.9 (1.8)	0	80.4	yes	.	Adolescent Medicine Trials Network for HIV/AIDS Interventions & International Maternal Pediatric Adolescent AIDS Clinical Trials sites	8
**Njamnshi et al.**	**2008**	Cameroon	IHDS (10)	204	21.1	37.2 (8.8)	.	31.4	yes	2006	The Day Care Hospital	7
**Nyamayaro et al.**	**2020**	Zimbabwe	GDS	155	49.7	37.8 (11.2)	100	30	only neurological, psychiatric disorders & alcohol use excluded	2018	.	8
**Odiase, Ogunrin & Ogunniyi**	**2006**	Nigeria	CSID	192	65.6	32.5 (7)	0	50	yes	2004	UniversityTeachingHospital,BeninCity	8
**Oshinaike et al.**	**2012**	Nigeria	MMSE (26)	208	2.9	36.8 (8.3)	100	34.1	yes	2007-2008	Lagos University Teaching Hospital (LUTH)	8
**Overton et al.**	**2013**	USA	MoCA	200	51	43.3 (10.7)	100	72	yes	.	Washington University in St. Loui	8
**Patel et al.**	**2010**	Malawi	IHDS (10)	179	14	18-65	74.9	35.2	only CNS infections & alcohol use excluded	2007	ART clinic of Queen Elizabeth Central Hospital in Blantyre	8
**Pereda et al.**	**2000**	Spain	Frascati (HAD)	100	27	33.6 (6.2)	51	71	only substance use excluded	1996-1997	HIV clinic at Marques de Valdecilla University Hospital in Santander	7
**Perez-Valero et al.**	**2013**	Spain	Frascati	191	27.2 (19.9, 7.3, 0)	45 (41-48)	100	73.3	yes	2011-2012	HIV Units of La Paz and the Doce de Octubre Hospitals in Madrid	8
**Pinheiro et al.**	**2016**	Brazil	IHDS (10)	392	54.1	42.8 (11.6)	89.3	44.7	yes	2015	Serviço de Assistência Especializada (SAE), Pelotas	8

**Portilla et al.**	**2019**	Spain	Frascati	84	29.8 (19, 8.3, 2.4)	46 (7.5)	100	77.4	yes	2014-2015	Infectious Diseases Unit of the General University Hospital of Alicante	8
**Pumpradit et al.**	**2010**	Thailand	Frascati	64	37.5 (21.9, 14.1, 1.6)	41 (36-46)	100	59.4	only CNS infections & head injuries excluded	.	.	6
**Qiao et al.**	**2019**	China	MMSE	669	12.4	47.7 (11.2)	92.8	66.7	no	2017	Comparative HIV and Aging Research in Taizhou (CHART)	7
**Robbins et al.**	**2011**	South Africa	IHDS (10)	65	80	38.5 (9.3)	100	35.4	only psychiatric disorders excluded	.	general medical clinic in the Western Cape Region of South Africa	6
**Robertson et al.**	**2019**	Brazil, India, Malawi, Peru, South Africa, Thailand, Zimbabwe	Frascati (uncategorised)	860	45	34'median'	0	47.7	only psychiatric disorders excluded	.	AIDS Clinical Trials Group (ACTG) 5175, not ALLRT according to Smurzynski et al. (2008)	8
**Robertson et al.**	**2014**	Canada	BNCS	2863	41.5	43 (19-83)	68.8	61.7	only opportunistic infections & substance use excluded	2010-2011	MULTICENTER study: CRANIUM study	8
**Rodrigues et al.**	**2013**	Brazil	Frascati (uncategorised)	187	52.4	44'median'	61.5	53.5	yes	.	.	7
**Ruhanya et al.**	**2020**	South Africa	GDS	147	36.7	31.5 (5.2)	17.8	16.6	unspecified	.	primary care HIV-1 clinics in Cape Town	7
**Sacktor et al.**	**2016**	USA	Frascati	364	33 (14, 14, 5)	47.4 (8.9)	74.7	100	yes	2007	Multicenter AIDS Cohort Study	8
**Sacktor et al.**	**2014**	Uganda	Frascati	117	92 (19, 32, 41)	36.7 (5.3)	0	33.3	yes	2009-2010	Infectious Disease Clinic in Kampala	8
**Saini & Barar**	**2016**	India	IHDS	80	32.5	21-50	100	56.3	only psychiatric disorders & substance use excluded	2011-2012	ART Centre of PBM and AG Hospital, Bikaner, Rajasthan	7
**Salahuddin et al.**	**2020**	Ethiopia	IHDS (10)	244	39.3	.	.	36	only neuropsychiatric medications excluded	2018	Mizan-Tepi University Teaching Hospital (MTUTH), Aman	7
**Salawu et al.**	**2008**	Nigeria	Frascati (uncategorised)	60	56.7	32 (7.6)	0	40	only psychiatric disorders, head injuries & history of substance use excluded	.	.	6
**Sanmarti et al.**	**2020**	Tanzania	Frascati (uncategorised)	243	19.3	44.3 (36-52)	100	29	yes	.	Chronic Diseases Clinic of Ifakara (CDCI)	8
**Sereia et al.**	**2012**	Brazil	MMSE	100	27	20-64	100	62	no	.	Parana	7
**Sevigny et al.**	**2007**	USA	Frascati (uncategorised)	329	68.5	41.9 (7.2)	76.8	68.6	only neurologic disorders, psychiatric disorders & CNS infections excluded	1998-2002	Northeast AIDS Dementia Study cohort	8
**Sheppard et al.**	**2015**	USA	Frascati (uncategorised)	75	16	56.4 (5.9)	100	84	yes	.	greater San Diego community and local, urban HIV clinics	7
**Simioni et al.**	**2010**	Switzerland	Frascati	200	84 (24, 52, 8)	46 (30-69.6)	100	72	only CNS infections & substance use excluded	.	Lausanne and Geneva University hospitals	7
**Spector et al.**	**2010**	China	GDS	201	36.8	40.2 (6.4)	56.7	60.7	yes, BUT 93% HCV COINFECTION	.	Anhui	8

**Starace et al.**	**2002**	Italy	Gisslen (uncategorised)	395	17.9	35.2 (8.1)	100	67.8	only psychiatric disorders excluded	1999-2000	NeuroICONA study	8
**Stern et al.**	**1991**	USA	Frascati (uncategorised)	124	58.1	38.4 (8.2)	.	100	unspecified	.	.	6
**Su et al.**	**2016**	Netherlands	MNC	103	17	54 (48-61)	100	100	yes	2011-2013	AGEhIV Cohort Study	7
**Suarez et al.**	**2001**	France	MMSE	91	58.2	38 (25-54)	87.9	85.7	yes	1995-1999	Paris University Hospital	7
**Sundermann et al.**	**2018**	USA	GDS	1361	42.7	42.6 (9.9)	67.5	85	yes	.	UCSD HRNP (HIV Neurobehavioral Research Program)	8
**Tamargo et al.**	**2021**	USA	MMSE (24)	394	14.7	53.9 (7.9)	.	53.6	no	2016-2020	MASH cohort	6
**Temereanca et al.**	**2020**	Romania	GDS	214	35	24, 19-29	100	48	unspecified	2012-2014	.	7
**Thiyagarajan et al.**	**2010**	UK	Frascati (uncategorised)	72	18	47 (10)	97	83.3	only neurological & substance use excluded, 37.5% HCV COINFECTION	.	St Mary's Hospital, London	7
**Tilghman et al.**	**2014**	India	GDS	155	36.1	34.5 (7.2)	0	57.1	yes	.	National AIDS Research Institute (NARI) in Pune	8
**Tomita et al.**	**2019**	South Africa	IHDS (10)	151	43.5	21-59	100	15.2	only substance use excluded, 100% MDR-TB	2015-2016	TB-specialist hospital in KwaZulu-Natal (KZN) Province	8
**Tozzi et al.**	**2005**	Italy	Frascati (uncategorised)	412	54.4	.	59.7	71.1	yes	1996-2004	Infectious Diseases Lazzaro Spallanzani, Rome	8
**Tremont-Lukats, Teixeira & Hernandez**	**1999**	Venezuela	MMSE (24)	75	36	34, 18-57	0	92	only neurological & substance use history excluded	.	Hospital Vargas de Caracas, Venezuela	7
**Troncoso & de Oliveira Contero**	**2015**	Brazil	IHDS (10)	111	52.2	46.7 (11.6)	87.7	56.8	only opportunistic infections excluded	2013	Infectious Diseases of the Faculdade de Medicina de Marília (FAMEMA), Marília City, State of São Paulo	7
**Trunfio et al.**	**2018**	Italy	Frascati	650	21.6 (15.7, 4.9, 1)	50 (42-58)	89.1	76.3	yes	2010-2017	Amedeo di Savoia Hospital, Turin	8
**Tsegaw et al.**	**2017**	Ethiopia	IHDS (9.5)	593	36.4	38.6 (10.6)	100	47.9	only psychiatric disorders excluded	.	Dessie Referral Hos- pital (DRH) and Kombolcha Health Center (KHC) HIV care clinic	8
**Underwood et al.**	**2019**	UK	NMM	639	21.4	57 (53-62)	98.8	88.6	no	.	POPPY study (COBRA and CHARTER excluded since separate larger cohort analysis available)	8
**Valcour et al.**	**2013**	Thailand	Frascati	61	45.9 (22.9, 13.1, 9.9)	34.7 (6.9)	0	43	yes	2009-2011	.	7
**Valcour et al.**	**2004**	USA	adpated MSK staging	202	19.7	45.6 (11.3)	72.7	82.8	only neurological & psychiatric disorders excluded	2001	Hawaii Aging with HIV Cohort Study	8
**van den Dries et al.**	**2017**	Netherlands	GDS	69	40.6	53 (11)	.	82.6	only neurological disorders excluded	2012-2013	The Dutch TREVI Cohort Study	7
**van Gorp et al.**	**1999**	USA	Frascati (HAD)	130	16.2	41 (8)	.	100	unspecified	1995	.	6
**Vassallo et al.**	**2015**	France	Frascati	204	29.9 (20.1, 7.8, 2)	51.9 (10.1)	.	77.5	only neurological disorders & opportunistic infections excluded	2007-2013	Neuradapt & Eldadapt studies	8
**Vergori et al.**	**2019**	Italy	Frascati	542	22.7 (16.2, 5.7, 0.7)	49 (42-56)	96.7	81	yes	2011-2016	.	8

**Villa et al.**	**1996**	Italy	(2 SD in at least 5 out of 24 measures)	78	28.2	32.3 (6.9)	.	64.1	yes	.	.		6
**Vitiello et al.**	**2007**	USA	GDS	179	19	39.6 (7.5)	67	96.1	only psychiatric disorders & subtance use excluded	1991-1994	.		7
**Wang et al.**	**2019**	USA	MNC	1531	7.45	38.6 (8.4)	.	100	no	.	Multicenter AIDS Cohort Study		7
**Wang et al.**	**2013**	China	MoCA	309	48.2	34 (28-43.5)	76.4	88	only neurological disorders & substance use excluded	2012-2013	Shanghai Public Health Clinical Center		8
**Widyadharma et al.**	**2017**	Indonesia	MMSE (25)	96	33.3	15-49	77.1	68.8	yes	2008-2009	Edelweiss Clinic Dr. Sardjito Hospital		7
**Wilkins et al.**	**1991**	USA	(below defined cut-offs in at least 2 out of 10 measures)	77	36.4	33.5 (8.3)	.	92.2	yes	.	.		6
**Winston et al.**	**2013**	UK	Frascati (uncategorised)	557	51.2	44 (9)	100	77	no	.	PIVOT study		8
**Wojna et al.**	**2007**	Puerto Rico	Frascati	60	68.4 (30, 11.7, 26.7)	36.4 (7)	80	0	only neurological disorders excluded	.	NeuroAIDS Specialized Neuroscience Research Program (SNRP)		8
**Wright et al.**	**2015**	Australia, Thailand, Brazil, Argentina, Chile, USA, UK, Belgium, Italy, Switzerland & Germany	Frascati (uncategorised)	608	19.9	34'median'	0	89	no	.	MULTICENTER START study		7
**Wright et al.**	**2008**	Thailand, China, Indonesia, Malaysia, Cambodia, Papua New Guinea, Fiji	Frascati (HAD)	647	11.7	35.9 (9.5)	65	59.3	no	2005-2006	MULTISITE Asia Pacific NeuroAIDS Consortium (APNAC)		8
**Wubetu, Asefa & Gebregiorgis**	**2021**	Ethiopia	MMSE (25)	422	41	20-64	100	39.8	no	2019-2020	public hospitals of North Shoa Zone		8
**Xiao et al.**	**2020**	China	MoCA (26)	250	87.2	65.7 (5.73)	100	70.8	only psychiatric disorders, sensory impairments & head injuries exluded	2017	Hunan province's main HIV clinics		8
**Yakasai et al.**	**2015**	Nigeria	Frascati	80	76.3 (41.3, 23.8, 11.3)	36.8 (8.9)	50	55	yes	.	Aminu Kano Teaching Hospital (AKTH) in Northwestern Nigeria		8

**Yechoor et al.**	**2016**	Uganda	GDS	181	38	36 (27-41)	80.1	42	only neurological & psychiatric disorders excluded	2011	Mulago Hospital of Makerere University in Kampala	8
**Yideg et al.**	**2019**	Ethiopia	IHDS (10)	328	35.7	38.2 (10.5)	100	41.8	yes	2018	Jimma University Medical Center (JUMC), Jimma	8
**Yusuf et al.**	**2017**	Nigeria	Frascati	418	21.5 (9.6, 9.1, 2.9)	37.2 (9.3)	100	22.3	no	.	Ahmadu Bello University Teaching Hospital (ABUTH), Zaria	8
**Zaegel-Faucher et al.**	**2020**	France	Frascati	121	57 (28.9, 24.8, 3.3)	53.1'median'	.	68	yes	2011-2018	.	6
**Zamudio-Rodiguez et al.**	**2018**	Mexico	Frascati	206	66 (60.2, 5.8, 0)	60.5 (6.3)	100	84.9	only conditions that may be mistaken as physical frailty excluded	2014-2016	ambulatory care at the HIV clinic of a university-affiliated tertiary care center in Mexico City	8
**Zhang et al.**	**2012**	China	Frascati	134	37.3 (22.4, 10.4, 4.5)	38.6 (9.5)	73.1	56.7	yes	.	Infectious Diseases Hospital of Henan Province, Infectious Diseases Hospital of Yunnan Province & Beijing You An Hospital	8
**Zhao et al.**	**2015**	China	Frascati	230	37.4 (18.3, 10.9, 8.3)	49.2 (10.2)	97.8	66.1	yes	.	Fourth People’s Hospital of Nanning and Guangxi Longtan Hospital	8

Supplementary Table 2: Raw data summary. The table summarizes the characteristics of the 225 eligible studies for meta-analysis. Table made using PowerPoint.

	North America	Latin America & Carribean	Europe & Central Asia	East Asia & Pacific	South Asia	Sub-Saharan Africa	Middle East & North Africa
**no. of studies**	54	13	58	35	10	48	1
**publishing years (range)**	1989 – 2021	1999 – 2020	1994 – 2021	2006 – 2021	1997– 2020	2006 – 2021	2020
**countries (no. of studies)**	USA (48), Canada (6)	Puerto Rico (1), Venezuela (1), Mexico (1), Brazil (10)	Switzerland (4), Netherlands (3), France (5), Italy (19), Germany (2), UK (10), Spain (8), Belarus (1), Denmark (1), Portugal (2), Romania (2), Ireland (1), Norway (1), Serbia (1), Turkey (1)	Indonesia (4), South Korea (2), Singapore (1), Japan (2), Malaysia (2), Thailand (7), Australia (6), South Korea (2), China (14)	India (10)	Tanzania (3), South Africa (8), Malawi (2), Kenya (4), Nigeria (10), Botswana (2), Uganda (7), Cameroon (2), Ethiopia (7), Ghana (1), Zimbabwe (1), Zambia (4)	Iran (1)
**net sample size**	20,074	2,078	13,894	7,704	1,494	13,662	93
**assessment type**<br>■ diagnostic<br>■ screening<br>■ other	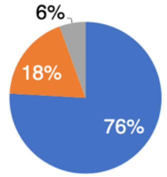	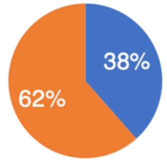	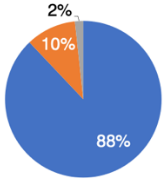	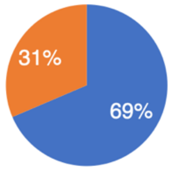	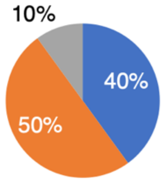	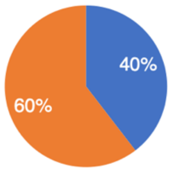	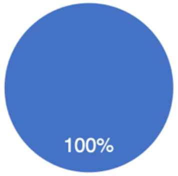
**exclusion criteria**<br>■ yes<br>■ partial<br>■ no<br>■ unspecified	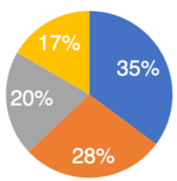	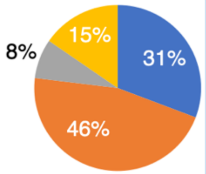	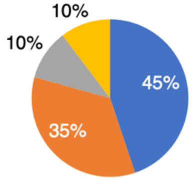	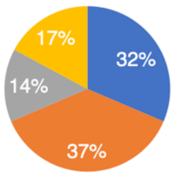	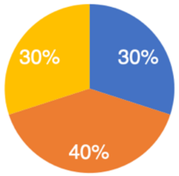	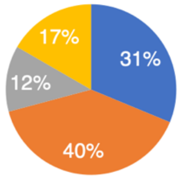	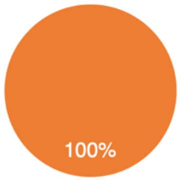
**income level**<br>■ high income<br>■ upper middle income<br>■ lower middle income<br>■ low income<br>■ mixed / unclassified	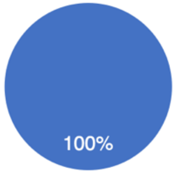	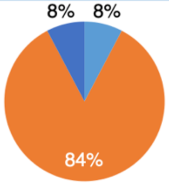	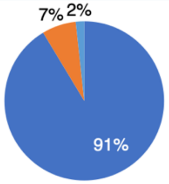	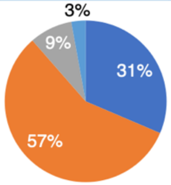	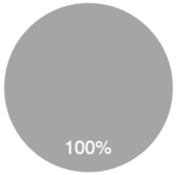	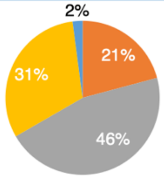	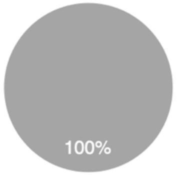
**age range**	16 – 83<br>*not reported in 2 studies	18 – 81	18 – 84<br>*not reported in 4 studies	18 – 83	18 – 74<br>*not reported in 1 study	18 – 75<br>*not reported in 2 studies	18 – 64
**ART coverage**	76.2%<br>*not reported in 17 studies	85.8%<br>*not reported in 2 studies	92.8%<br>*not reported in 11 studies	78.1%<br>*not reported in 6 studies	59.4%<br>*not reported in 2 studies	75.3%<br>*not reported in 4 studies	92.5%
**male gender**	80.7%	57.7%	78.2%	73.9%<br>*not reported in 2 studies	62.4%<br>*not reported in 1 study	37.4%	60.2%

Supplementary Figure 1: Forest plot of study subgroups by geographical region (country). The subgroup "multiple regions" refers to multi-site studies. Figure made using STATA.

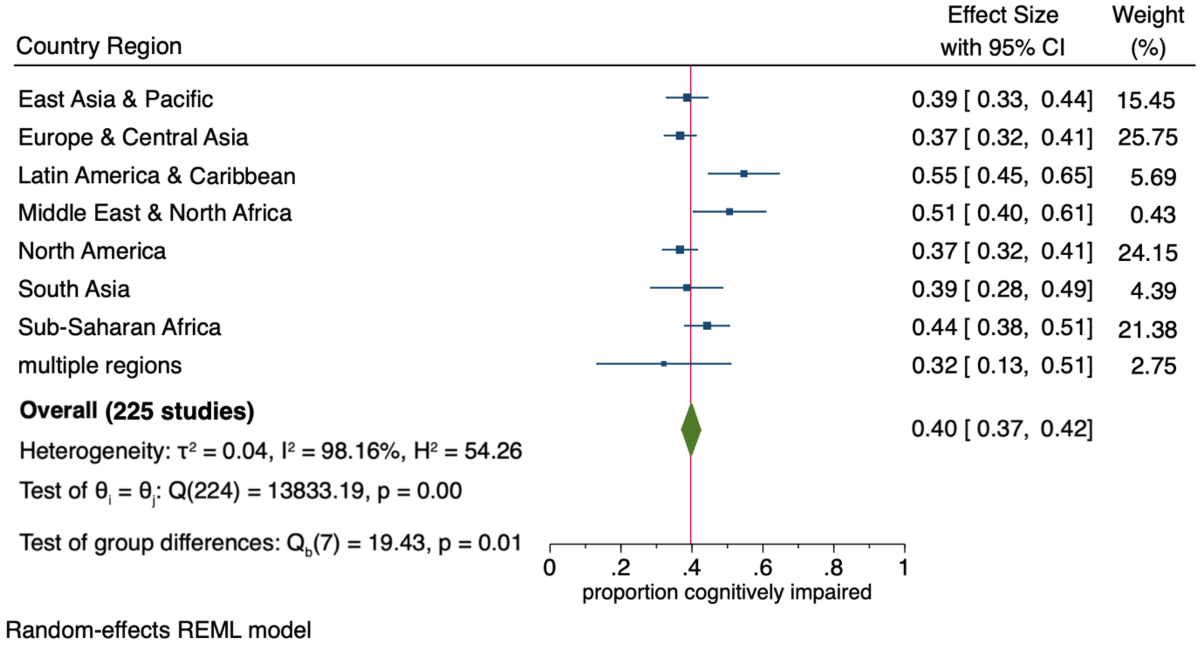

Supplementary Figure 2: Forest plot including studies from East Asia & Pacific.

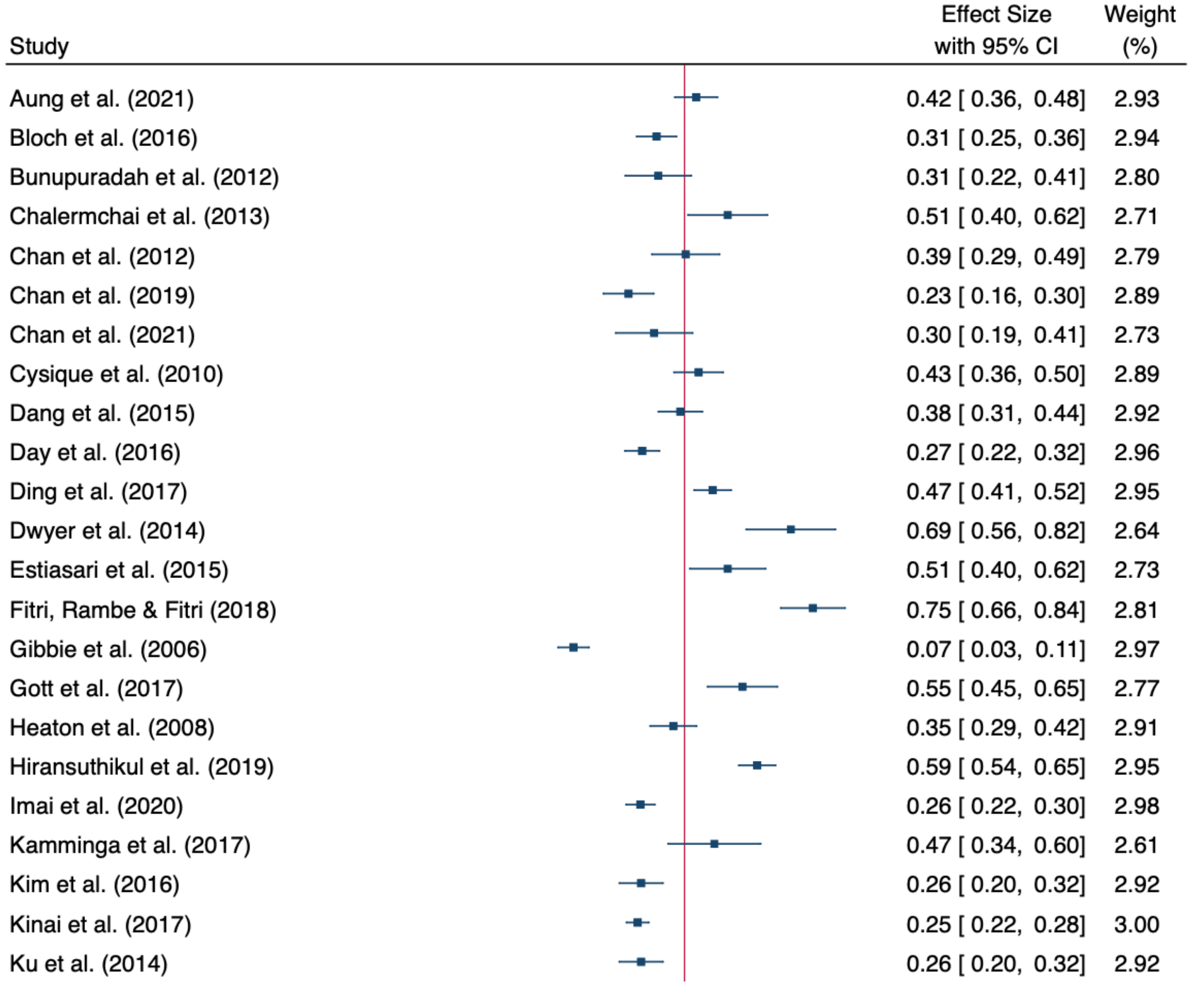

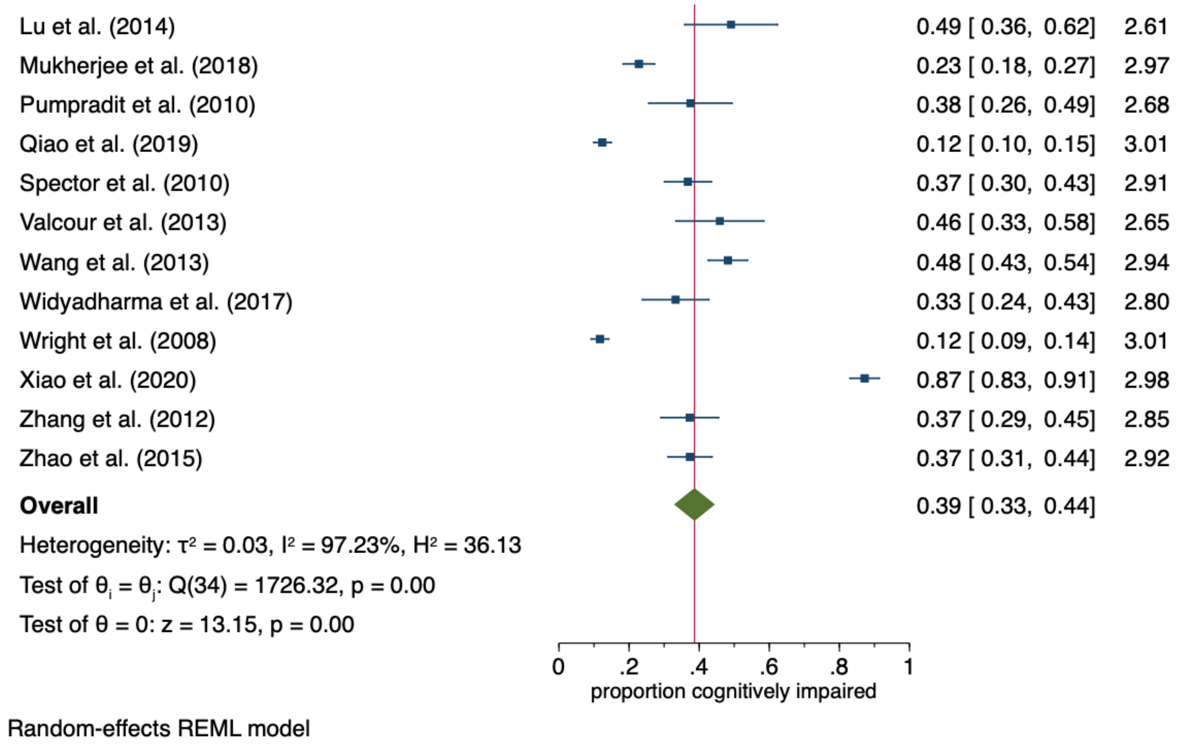

Supplementary Figure 3: Forest plot including studies from Europe & Central Asia.

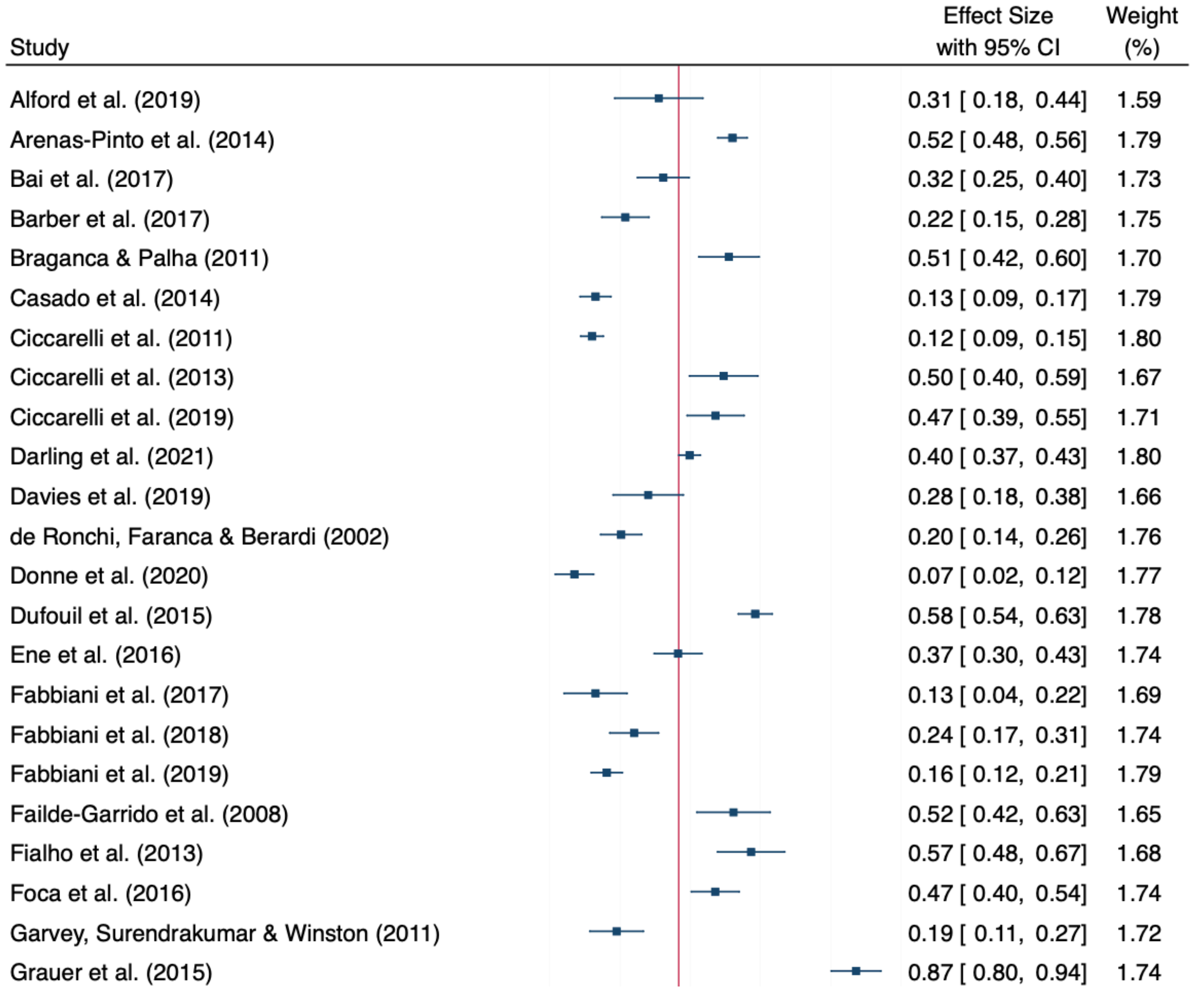

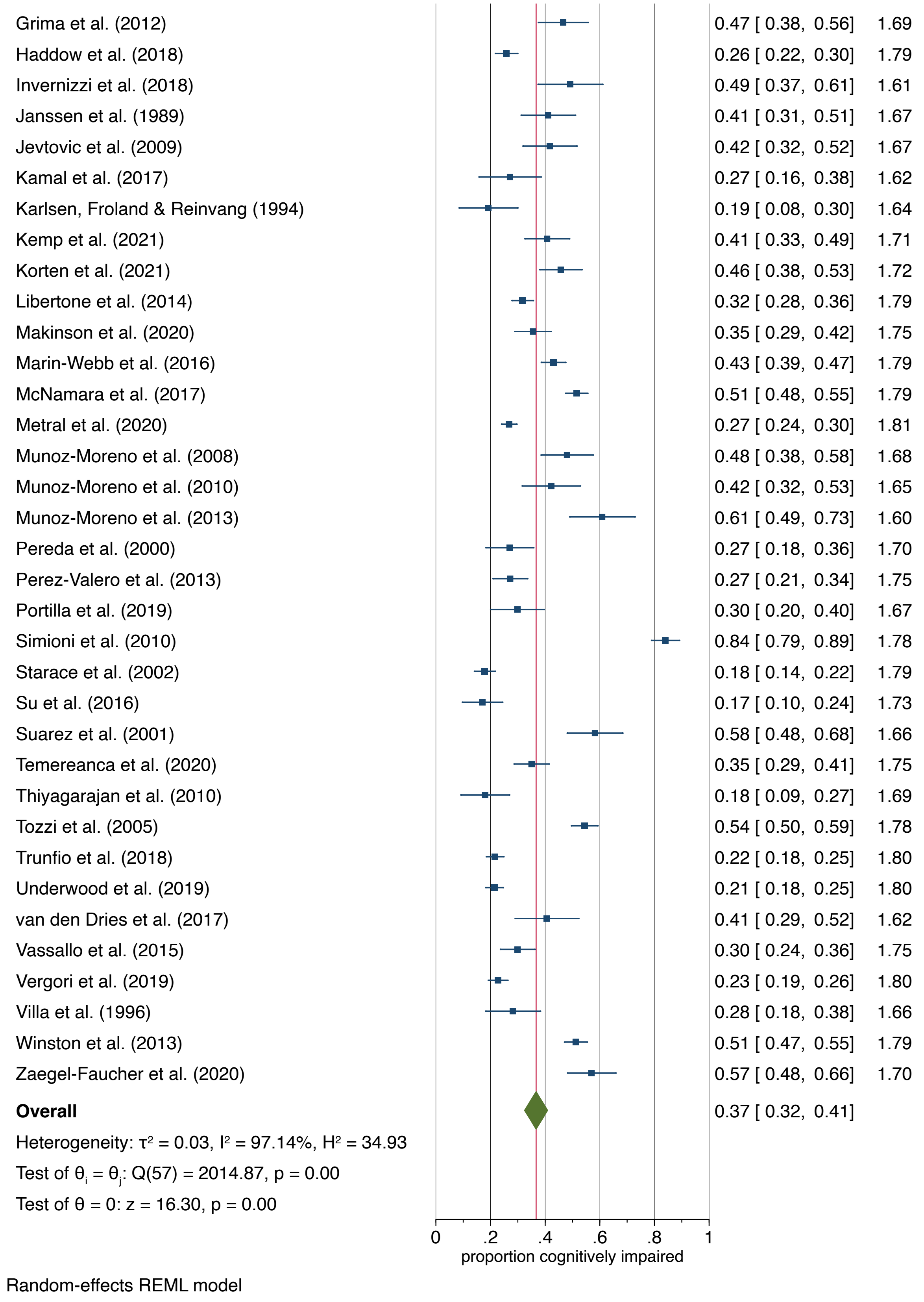
Grima et al. (2012) 0.47 [0.38, 0.56] 1.69
Haddow et al. (2018) 0.26 [0.22, 0.30] 1.79
Invernizzi et al. (2018) 0.49 [0.37, 0.61] 1.61
Janssen et al. (1989) 0.41 [0.31, 0.51] 1.67
Jevtovic et al. (2009) 0.42 [0.32, 0.52] 1.67
Kamal et al. (2017) 0.27 [0.16, 0.38] 1.62
Karlsen, Froland & Reinvang (1994) 0.19 [0.08, 0.30] 1.64
Kemp et al. (2021) 0.41 [0.33, 0.49] 1.71
Korten et al. (2021) 0.46 [0.38, 0.53] 1.72
Libertone et al. (2014) 0.32 [0.28, 0.36] 1.79
Makinson et al. (2020) 0.35 [0.29, 0.42] 1.75
Marin-Webb et al. (2016) 0.43 [0.39, 0.47] 1.79
McNamara et al. (2017) 0.51 [0.48, 0.55] 1.79
Metral et al. (2020) 0.27 [0.24, 0.30] 1.81
Munoz-Moreno et al. (2008) 0.48 [0.38, 0.58] 1.68
Munoz-Moreno et al. (2010) 0.42 [0.32, 0.53] 1.65
Munoz-Moreno et al. (2013) 0.61 [0.49, 0.73] 1.60
Pereda et al. (2000) 0.27 [0.18, 0.36] 1.70
Perez-Valero et al. (2013) 0.27 [0.21, 0.34] 1.75
Portilla et al. (2019) 0.30 [0.20, 0.40] 1.67
Simioni et al. (2010) 0.84 [0.79, 0.89] 1.78
Starace et al. (2002) 0.18 [0.14, 0.22] 1.79
Su et al. (2016) 0.17 [0.10, 0.24] 1.73
Suarez et al. (2001) 0.58 [0.48, 0.68] 1.66
Temereanca et al. (2020) 0.35 [0.29, 0.41] 1.75
Thiyagarajan et al. (2010) 0.18 [0.09, 0.27] 1.69
Tozzi et al. (2005) 0.54 [0.50, 0.59] 1.78
Trunfio et al. (2018) 0.22 [0.18, 0.25] 1.80
Underwood et al. (2019) 0.21 [0.18, 0.25] 1.80
van den Dries et al. (2017) 0.41 [0.29, 0.52] 1.62
Vassallo et al. (2015) 0.30 [0.24, 0.36] 1.75
Vergori et al. (2019) 0.23 [0.19, 0.26] 1.80
Villa et al. (1996) 0.28 [0.18, 0.38] 1.66
Winston et al. (2013) 0.51 [0.47, 0.55] 1.79
Zaegel-Faucher et al. (2020) 0.57 [0.48, 0.66] 1.70
Overall 0.37 [0.32, 0.41]
Heterogeneity: τ^2 = 0.03, I^2 = 97.14%, H^2 = 34.93
Test of $\theta_i = \theta_j$: Q(57) = 2014.87, p = 0.00
Test of θ = 0: z = 16.30, p = 0.00
0 .2 .4 .6 .8 1
proportion cognitively impaired
Random-effects REML model

Supplementary Figure 4: Forest plot including studies from Latin America & Caribbean.

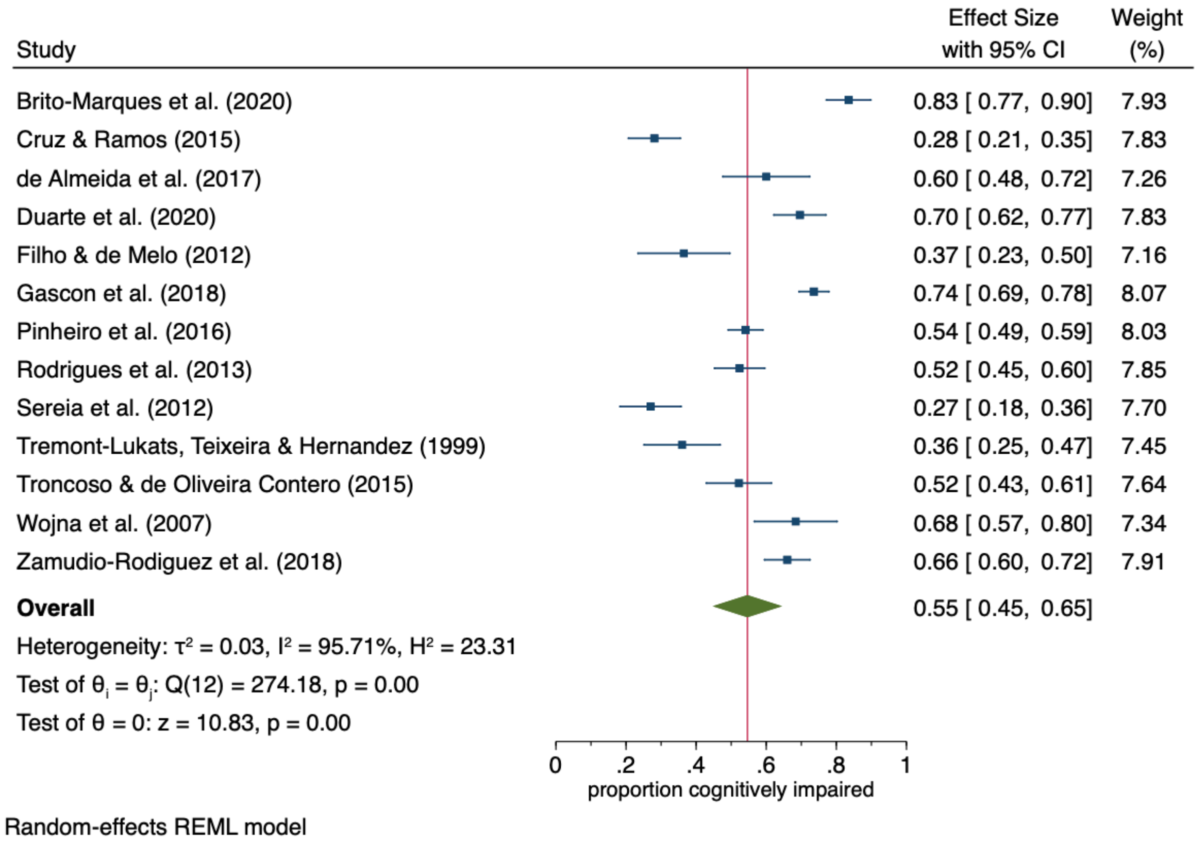

Supplementary Figure 5: Forest plot including studies from Middle East & North Africa.

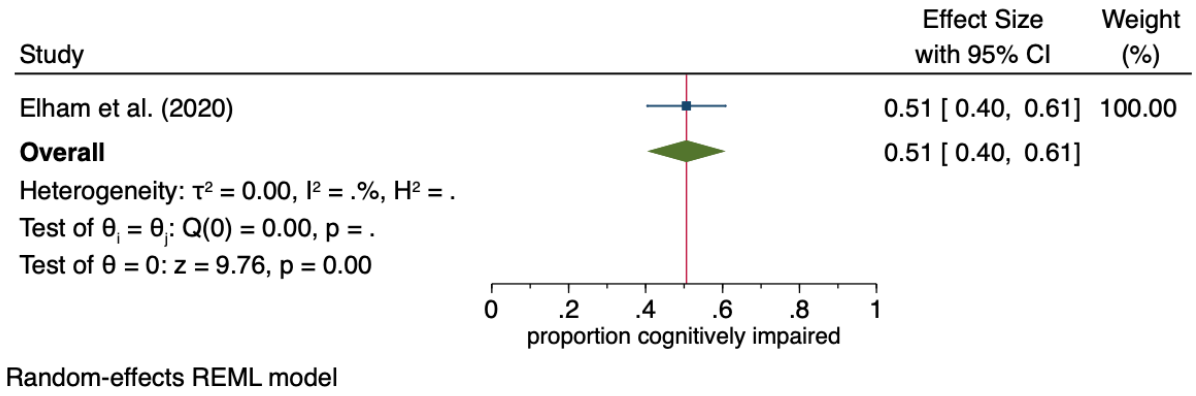

Supplementary Figure 6: Forest plot including studies from North America.

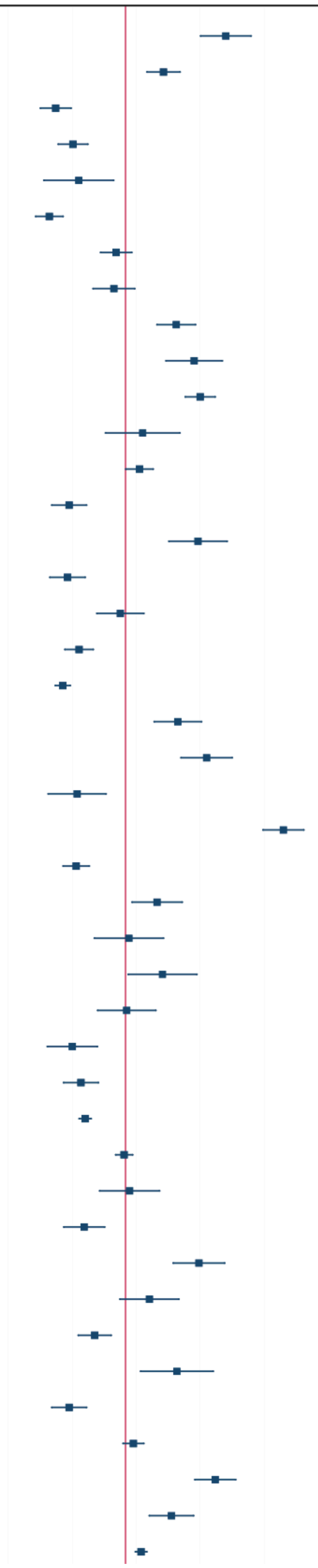

Study	Effect Size with 95% CI	Weight (%)
Arentoft et al. (2015)	0.68 [0.60, 0.76]	1.83
Atkins et al. (2010)	0.49 [0.43, 0.54]	1.88
Banerjee, McIntosh & Ironson (2019)	0.15 [0.10, 0.20]	1.89
Becker et al. (2004)	0.20 [0.16, 0.25]	1.89
Boccellari et al. (1993)	0.22 [0.11, 0.33]	1.75
Bornstein et al. (1993)	0.13 [0.08, 0.17]	1.89
Bourgeois et al. (2020)	0.34 [0.29, 0.39]	1.89
Brouillete et al. (2015)	0.33 [0.26, 0.40]	1.86
Brouillette et al. (2021)	0.52 [0.46, 0.59]	1.87
Bryant et al. (2015)	0.58 [0.49, 0.67]	1.81
Carvalhal et al. (2016)	0.60 [0.55, 0.65]	1.89
Chang et al. (2011)	0.42 [0.30, 0.54]	1.73
Cross et al. (2013)	0.41 [0.37, 0.45]	1.90
Crum-Cianflone et al. (2013)	0.19 [0.14, 0.24]	1.88
Dampier et al. (2017)	0.59 [0.50, 0.68]	1.80
Deiss et al. (2019)	0.19 [0.13, 0.24]	1.88
Derry et al. (2020)	0.35 [0.28, 0.42]	1.84
Dufour et al. (2013)	0.22 [0.18, 0.27]	1.89
Erlandson et al. (2019)	0.17 [0.15, 0.19]	1.92
Fazeli, Woods & Vance (2019)	0.53 [0.46, 0.60]	1.84
Ferrando et al. (2003)	0.62 [0.54, 0.70]	1.83
Foley et al. (2013)	0.22 [0.12, 0.31]	1.80
Gandhi et al. (2011)	0.86 [0.80, 0.92]	1.86
Gomez et al. (2019)	0.21 [0.17, 0.25]	1.90
Greene et al. (2015)	0.47 [0.39, 0.54]	1.83
Groff et al. (2020)	0.38 [0.27, 0.49]	1.75
Halman et al. (2014)	0.48 [0.37, 0.59]	1.76
Hanna et al. (2020)	0.37 [0.28, 0.46]	1.80
Janssen et al. (2015)	0.20 [0.12, 0.28]	1.83
Kalayjian et al. (2014)	0.23 [0.17, 0.28]	1.88
Kalayjian et al. (2019)	0.24 [0.22, 0.26]	1.92
Kallianpur et al. (2016)	0.36 [0.34, 0.39]	1.91
Klusman et al. (1991)	0.38 [0.29, 0.47]	1.79
Kupprat et al. (2017)	0.24 [0.17, 0.30]	1.86
Matchanova et al. (2020)	0.60 [0.52, 0.68]	1.83
Mayeux et al. (1993)	0.44 [0.35, 0.53]	1.80
McCutchan et al. (2007)	0.27 [0.22, 0.32]	1.88
Milanini et al. (2020)	0.53 [0.41, 0.64]	1.74
Moore et al. (2012)	0.19 [0.14, 0.24]	1.88
Naveed et al. (2021)	0.39 [0.36, 0.42]	1.91
Nichols et al. (2013)	0.65 [0.58, 0.71]	1.86
Overton et al. (2013)	0.51 [0.44, 0.58]	1.85
Robertson et al. (2019)	0.41 [0.40, 0.43]	1.92

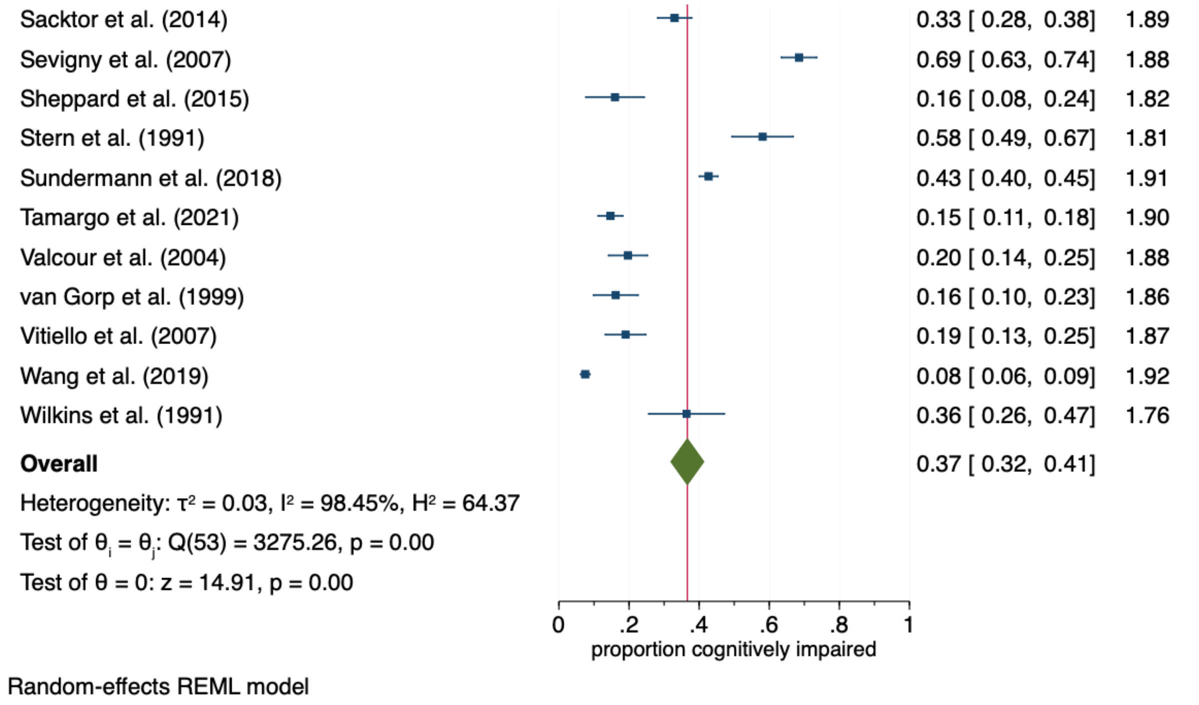

Supplementary Figure 7: Forest plot including studies from South Asia.

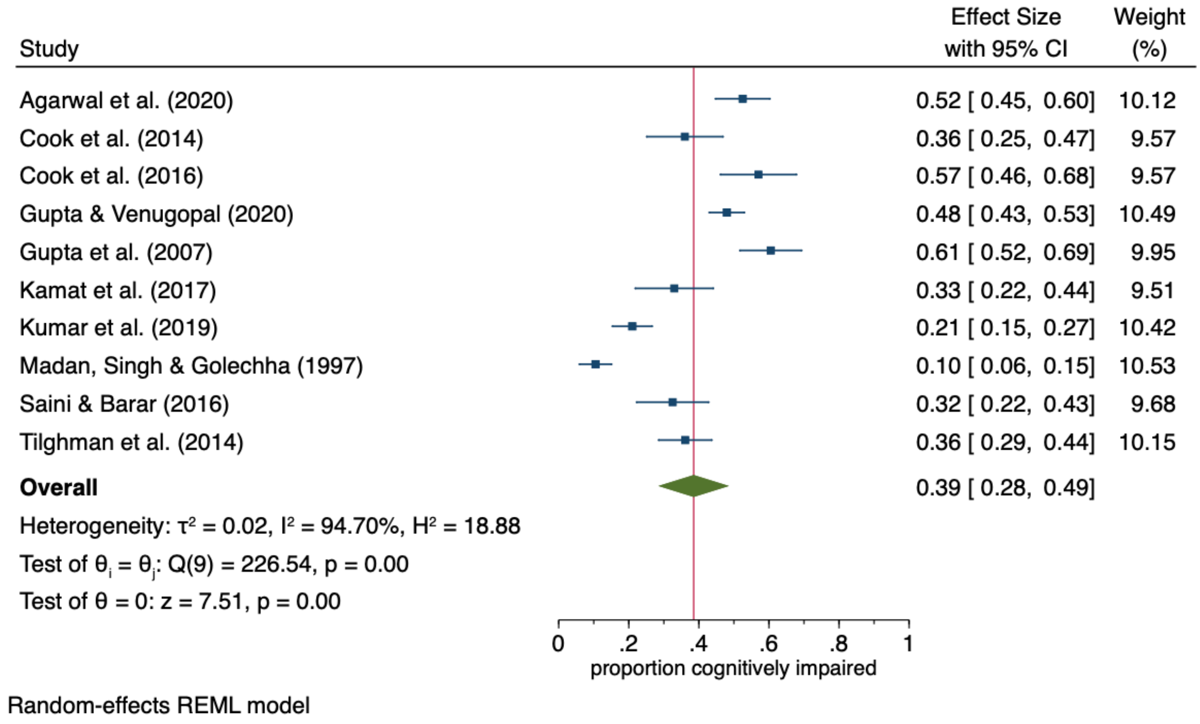

Supplementary Figure 8: Forest plot including studies from Sub-Saharan Africa.

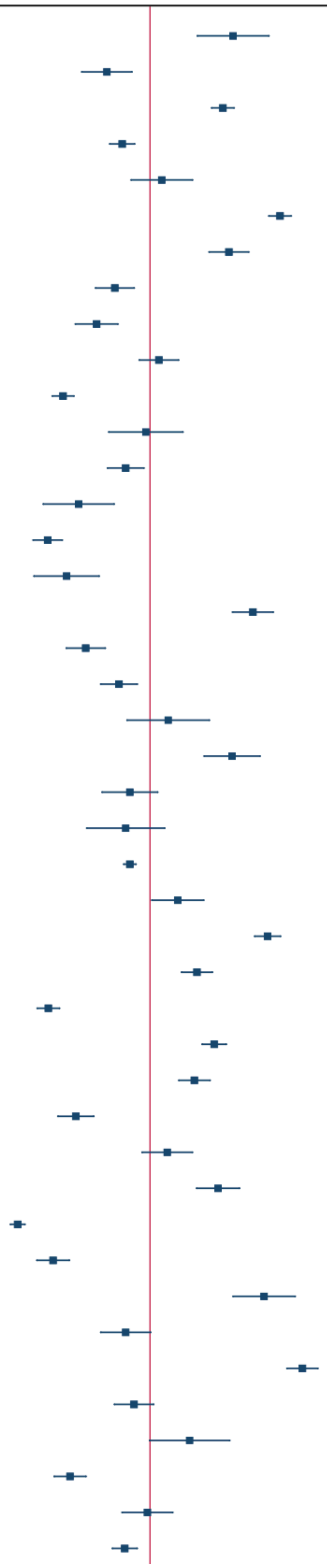

Study	Effect Size with 95% CI	Weight (%)
Akena et al. (2010)	0.70 [0.59, 0.81]	2.00
Akolo et al. (2014)	0.31 [0.23, 0.39]	2.07
Animut et al. (2019)	0.67 [0.64, 0.71]	2.13
Araya et al. (2020)	0.36 [0.32, 0.39]	2.12
Asiedu, Kretchy & Asampong (2020)	0.48 [0.38, 0.58]	2.04
Atashili et al. (2013)	0.85 [0.82, 0.88]	2.13
Awori et al. (2018)	0.69 [0.63, 0.75]	2.10
Belete, Medfu & Yemiyamrew (2017)	0.33 [0.27, 0.39]	2.10
Bharti et al. (2021)	0.28 [0.21, 0.34]	2.09
Flatt et al. (2021)	0.47 [0.41, 0.53]	2.10
Ganasen et al. (2008)	0.17 [0.14, 0.20]	2.13
Goodkin et al. (2014)	0.43 [0.31, 0.55]	1.99
Hestad et al. (2019)	0.37 [0.31, 0.42]	2.10
Holguin et al. (2011)	0.22 [0.11, 0.33]	2.01
Imam (2007)	0.12 [0.08, 0.17]	2.12
Joska et al. (2016)	0.18 [0.08, 0.28]	2.03
Joska et al. (2019)	0.76 [0.70, 0.83]	2.09
Jumare et al. (2019)	0.24 [0.18, 0.30]	2.10
Kabuba et al. (2017)	0.35 [0.29, 0.40]	2.10
Kalungwana et al. (2014)	0.50 [0.37, 0.63]	1.96
Kelly et al. (2014)	0.70 [0.61, 0.79]	2.06
Lawler et al. (2010)	0.38 [0.29, 0.47]	2.06
Lawler et al. (2011)	0.37 [0.25, 0.49]	1.98
Milanini et al. (2017)	0.38 [0.36, 0.40]	2.14
Mogambery et al. (2017)	0.53 [0.45, 0.61]	2.07
Mohamed, Oduor & Kinyanjui (2020)	0.81 [0.77, 0.85]	2.12
Molinaro et al. (2020)	0.59 [0.54, 0.64]	2.11
Mugendi et al. (2019)	0.12 [0.09, 0.16]	2.13
Nakku, Kinyada & Hoskins (2013)	0.64 [0.61, 0.68]	2.12
Namagga et al. (2019)	0.58 [0.53, 0.63]	2.11
Njamnshi et al. (2008)	0.21 [0.16, 0.27]	2.11
Nyamayaro et al. (2020)	0.50 [0.42, 0.58]	2.07
Odiase, Ogunrin & Ogunniyi (2006)	0.66 [0.59, 0.72]	2.09
Oshinaike et al. (2012)	0.03 [0.01, 0.05]	2.14
Patel et al. (2010)	0.14 [0.09, 0.19]	2.11
Robbins et al. (2011)	0.80 [0.70, 0.90]	2.04
Ruhanya et al. (2020)	0.37 [0.29, 0.44]	2.07
Sacktor et al. (2016)	0.92 [0.87, 0.97]	2.11
Salahuddin et al. (2020)	0.39 [0.33, 0.45]	2.10
Salawu et al. (2008)	0.57 [0.44, 0.69]	1.97
Sanmarti et al. (2020)	0.19 [0.14, 0.24]	2.11
Tomita et al. (2019)	0.44 [0.36, 0.51]	2.07
Tsegaw et al. (2017)	0.36 [0.33, 0.40]	2.12

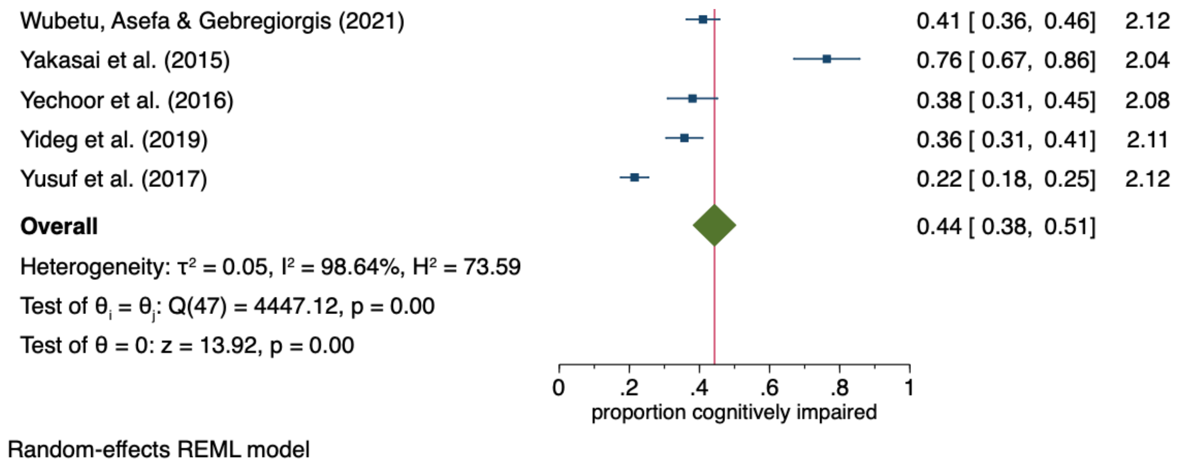
Wubetu, Asefa & Gebregiorgis (2021)
0.41 [0.36, 0.46] 2.12
Yakasai et al. (2015)
0.76 [0.67, 0.86] 2.04
Yechoor et al. (2016)
0.38 [0.31, 0.45] 2.08
Yideg et al. (2019)
0.36 [0.31, 0.41] 2.11
Yusuf et al. (2017)
0.22 [0.18, 0.25] 2.12
Overall
0.44 [0.38, 0.51]
Heterogeneity: τ^2 = 0.05, I^2 = 98.64%, H^2 = 73.59
Test of $\theta_i = \theta_j$: Q(47) = 4447.12, p = 0.00
Test of θ = 0: z = 13.92, p = 0.00
0
.2
.4
.6
.8
1
proportion cognitively impaired
Random-effects REML model

Supplementary Figure 9: Forest plot of study subgroups by neurological assessment type. The subgroup "other criteria" refers to six studies using unvalidated approaches. Figure made using STATA.

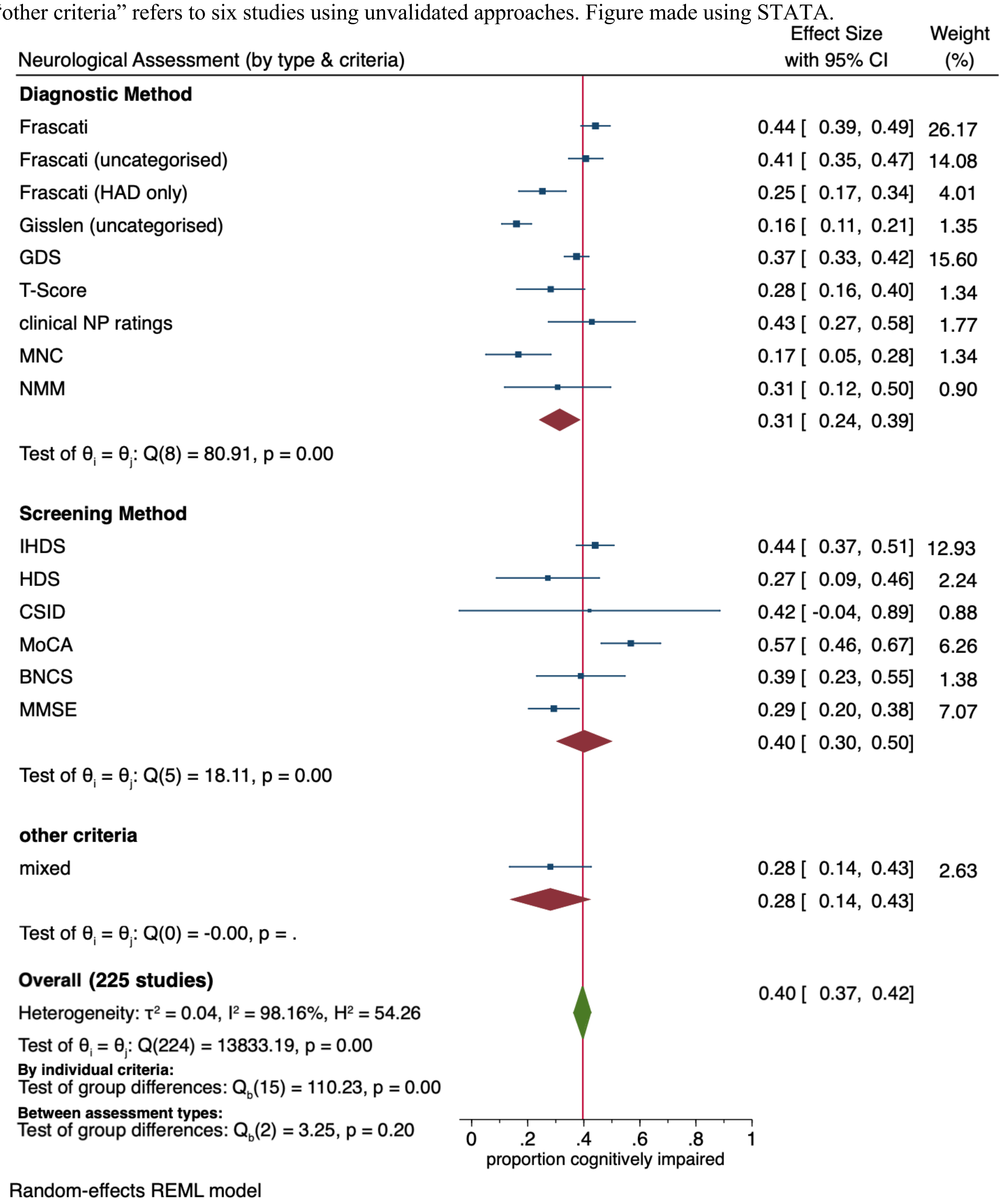

Supplementary Figure 10: Forest plot including studies categorised as a diagnostic method.

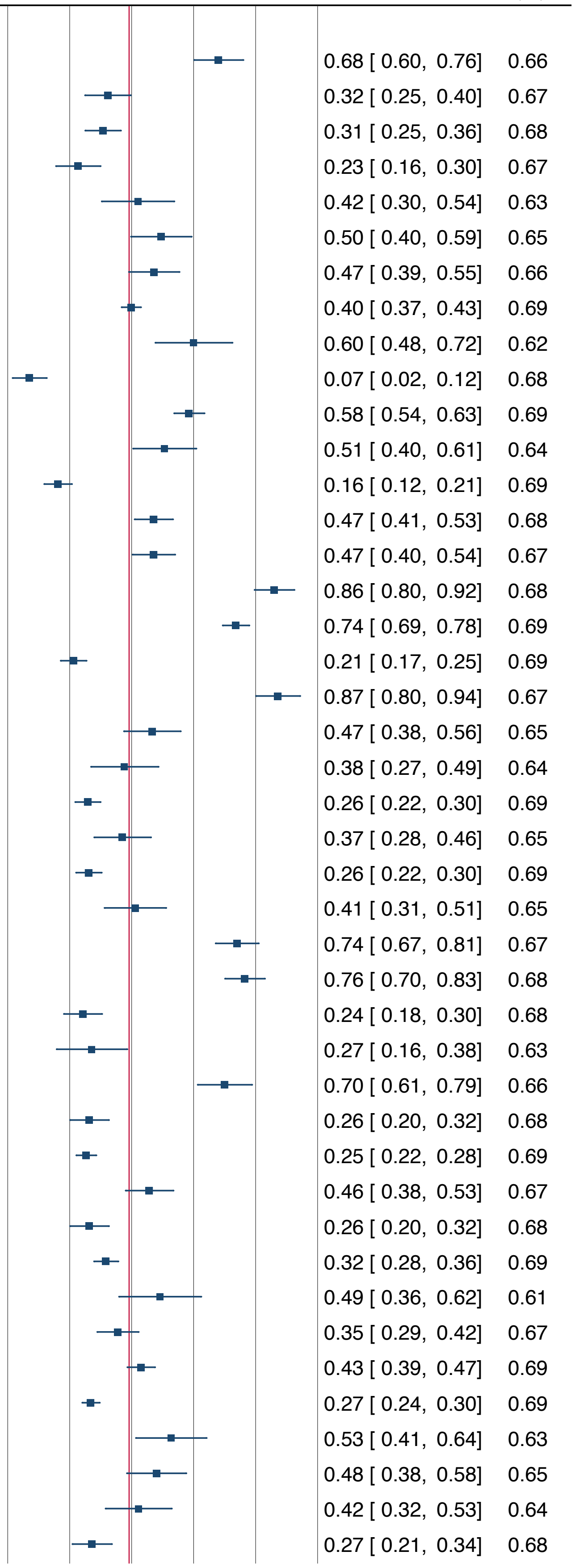

Study	Effect Size with 95% CI	Weight (%)
**Frascati**		
Arentoft et al. (2015)	0.68 [0.60, 0.76]	0.66
Bai et al. (2017)	0.32 [0.25, 0.40]	0.67
Bloch et al. (2016)	0.31 [0.25, 0.36]	0.68
Chan et al. (2019)	0.23 [0.16, 0.30]	0.67
Chang et al. (2011)	0.42 [0.30, 0.54]	0.63
Ciccarelli et al. (2013)	0.50 [0.40, 0.59]	0.65
Ciccarelli et al. (2019)	0.47 [0.39, 0.55]	0.66
Darling et al. (2021)	0.40 [0.37, 0.43]	0.69
de Almeida et al. (2017)	0.60 [0.48, 0.72]	0.62
Donne et al. (2020)	0.07 [0.02, 0.12]	0.68
Dufouil et al. (2015)	0.58 [0.54, 0.63]	0.69
Elham et al. (2020)	0.51 [0.40, 0.61]	0.64
Fabbiani et al. (2019)	0.16 [0.12, 0.21]	0.69
Flatt et al. (2021)	0.47 [0.41, 0.53]	0.68
Foca et al. (2016)	0.47 [0.40, 0.54]	0.67
Gandhi et al. (2011)	0.86 [0.80, 0.92]	0.68
Gascon et al. (2018)	0.74 [0.69, 0.78]	0.69
Gomez et al. (2019)	0.21 [0.17, 0.25]	0.69
Grauer et al. (2015)	0.87 [0.80, 0.94]	0.67
Grima et al. (2012)	0.47 [0.38, 0.56]	0.65
Groff et al. (2020)	0.38 [0.27, 0.49]	0.64
Haddow et al. (2018)	0.26 [0.22, 0.30]	0.69
Hanna et al. (2020)	0.37 [0.28, 0.46]	0.65
Imai et al. (2020)	0.26 [0.22, 0.30]	0.69
Janssen et al. (1989)	0.41 [0.31, 0.51]	0.65
Joska et al. (2011)	0.74 [0.67, 0.81]	0.67
Joska et al. (2019)	0.76 [0.70, 0.83]	0.68
Jumare et al. (2019)	0.24 [0.18, 0.30]	0.68
Kamal et al. (2017)	0.27 [0.16, 0.38]	0.63
Kelly et al. (2014)	0.70 [0.61, 0.79]	0.66
Kim et al. (2016)	0.26 [0.20, 0.32]	0.68
Kinai et al. (2017)	0.25 [0.22, 0.28]	0.69
Korten et al. (2021)	0.46 [0.38, 0.53]	0.67
Ku et al. (2014)	0.26 [0.20, 0.32]	0.68
Libertone et al. (2014)	0.32 [0.28, 0.36]	0.69
Lu et al. (2014)	0.49 [0.36, 0.62]	0.61
Makinson et al. (2020)	0.35 [0.29, 0.42]	0.67
Marin-Webb et al. (2016)	0.43 [0.39, 0.47]	0.69
Metral et al. (2020)	0.27 [0.24, 0.30]	0.69
Milanini et al. (2020)	0.53 [0.41, 0.64]	0.63
Munoz-Moreno et al. (2008)	0.48 [0.38, 0.58]	0.65
Munoz-Moreno et al. (2010)	0.42 [0.32, 0.53]	0.64
Perez-Valero et al. (2013)	0.27 [0.21, 0.34]	0.68

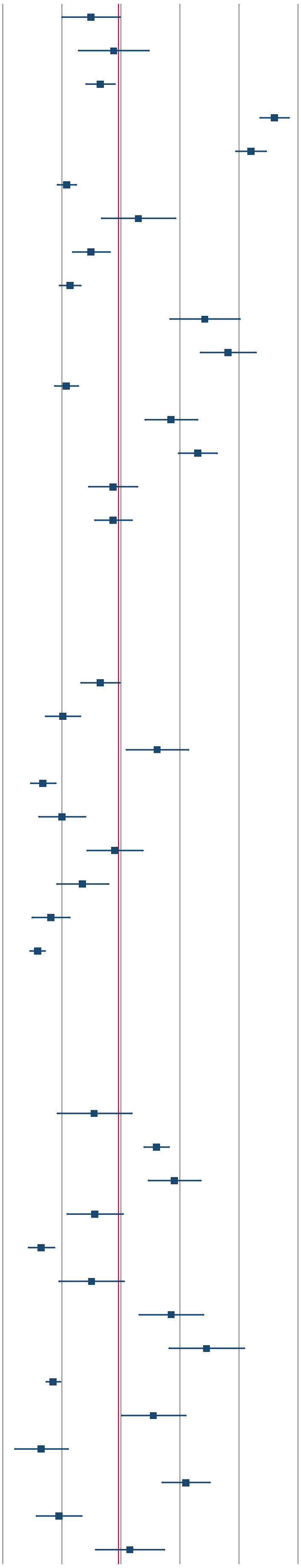

Portilla et al. (2019)	0.30 [0.20, 0.40]	0.65
Pumpradit et al. (2010)	0.38 [0.26, 0.49]	0.63
Sacktor et al. (2014)	0.33 [0.28, 0.38]	0.69
Sacktor et al. (2016)	0.92 [0.87, 0.97]	0.69
Simioni et al. (2010)	0.84 [0.79, 0.89]	0.68
Trunfio et al. (2018)	0.22 [0.18, 0.25]	0.69
Valcour et al. (2013)	0.46 [0.33, 0.58]	0.62
Vassallo et al. (2015)	0.30 [0.24, 0.36]	0.68
Vergori et al. (2019)	0.23 [0.19, 0.26]	0.69
Wojna et al. (2007)	0.68 [0.57, 0.80]	0.63
Yakasai et al. (2015)	0.76 [0.67, 0.86]	0.65
Yusuf et al. (2017)	0.22 [0.18, 0.25]	0.69
Zaegel-Faucher et al. (2020)	0.57 [0.48, 0.66]	0.66
Zamudio-Rodiguez et al. (2018)	0.66 [0.60, 0.72]	0.68
Zhang et al. (2012)	0.37 [0.29, 0.45]	0.66
Zhao et al. (2015)	0.37 [0.31, 0.44]	0.68
Heterogeneity: $\tau^2 = 0.04$, $I^2 = 97.92\%$, $H^2 = 48.02$	0.44 [0.39, 0.49]	
Test of $\theta_i = \theta_j$: $Q(58) = 2969.35$, $p = 0.00$		
**Frascati (HAD)**		
Brouillete et al. (2015)	0.33 [0.26, 0.40]	0.68
de Ronchi, Faranca & Berardi (2002)	0.20 [0.14, 0.26]	0.68
Failde-Garrido et al. (2008)	0.52 [0.42, 0.63]	0.64
Grund et al. (2013)	0.14 [0.09, 0.18]	0.69
Janssen et al. (2015)	0.20 [0.12, 0.28]	0.66
Klusman et al. (1991)	0.38 [0.29, 0.47]	0.65
Pereda et al. (2000)	0.27 [0.18, 0.36]	0.66
van Gorp et al. (1999)	0.16 [0.10, 0.23]	0.68
Wright et al. (2008)	0.12 [0.09, 0.14]	0.70
Heterogeneity: $\tau^2 = 0.02$, $I^2 = 94.90\%$, $H^2 = 19.60$	0.25 [0.17, 0.34]	
Test of $\theta_i = \theta_j$: $Q(8) = 113.01$, $p = 0.00$		
**Frascati (uncategorised)**		
Alford et al. (2019)	0.31 [0.18, 0.44]	0.62
Arenas-Pinto et al. (2014)	0.52 [0.48, 0.56]	0.69
Bryant et al. (2015)	0.58 [0.49, 0.67]	0.66
Bunupuradah et al. (2012)	0.31 [0.22, 0.41]	0.65
Casado et al. (2014)	0.13 [0.09, 0.17]	0.69
Chan et al. (2021)	0.30 [0.19, 0.41]	0.64
Cook et al. (2016)	0.57 [0.46, 0.68]	0.64
Dwyer et al. (2014)	0.69 [0.56, 0.82]	0.62
Erlandson et al. (2019)	0.17 [0.15, 0.19]	0.70
Estiasari et al. (2015)	0.51 [0.40, 0.62]	0.64
Fabbiani et al. (2017)	0.13 [0.04, 0.22]	0.66
Ferrando et al. (2003)	0.62 [0.54, 0.70]	0.66
Garvey, Surendrakumar & Winston (2011)	0.19 [0.11, 0.27]	0.67
Goodkin et al. (2014)	0.43 [0.31, 0.55]	0.63

Gupta & Venugopal (2020)	0.48 [0.43, 0.53]	0.68
Halman et al. (2014)	0.48 [0.37, 0.59]	0.64
Lawler et al. (2011)	0.37 [0.25, 0.49]	0.62
Mayeux et al. (1993)	0.44 [0.35, 0.53]	0.65
McCutchan et al. (2007)	0.27 [0.22, 0.32]	0.68
Milanini et al. (2017)	0.38 [0.36, 0.40]	0.70
Munoz-Moreno et al. (2013)	0.61 [0.49, 0.73]	0.62
Robertson et al. (2014)	0.45 [0.42, 0.48]	0.69
Rodrigues et al. (2013)	0.52 [0.45, 0.60]	0.67
Salawu et al. (2008)	0.57 [0.44, 0.69]	0.62
Sanmarti et al. (2020)	0.19 [0.14, 0.24]	0.68
Sevigny et al. (2007)	0.69 [0.63, 0.74]	0.68
Sheppard et al. (2015)	0.16 [0.08, 0.24]	0.66
Stern et al. (1991)	0.58 [0.49, 0.67]	0.66
Thiyagarajan et al. (2010)	0.18 [0.09, 0.27]	0.66
Tozzi et al. (2005)	0.54 [0.50, 0.59]	0.69
Winston et al. (2013)	0.51 [0.47, 0.55]	0.69
Wright et al. (2015)	0.20 [0.17, 0.23]	0.69
Heterogeneity: $\tau^2 = 0.03$, $I^2 = 97.55\%$, $H^2 = 40.90$	0.41 [0.35, 0.47]	
Test of $\theta_i = \theta_j$: $Q(31) = 1170.36$, $p = 0.00$		
**GDS**		
Akolo et al. (2014)	0.31 [0.23, 0.39]	0.66
Aung et al. (2021)	0.42 [0.36, 0.48]	0.68
Bharti et al. (2021)	0.28 [0.21, 0.34]	0.67
Braganca & Palha (2011)	0.51 [0.42, 0.60]	0.66
Brouillette et al. (2021)	0.52 [0.46, 0.59]	0.68
Carvalhal et al. (2016)	0.60 [0.55, 0.65]	0.69
Crum-Cianflone et al. (2013)	0.19 [0.14, 0.24]	0.68
Cysique et al. (2010)	0.43 [0.36, 0.50]	0.67
Dampier et al. (2017)	0.59 [0.50, 0.68]	0.65
Day et al. (2016)	0.27 [0.22, 0.32]	0.69
Deiss et al. (2019)	0.19 [0.13, 0.24]	0.68
Dufour et al. (2013)	0.22 [0.18, 0.27]	0.69
Ene et al. (2016)	0.37 [0.30, 0.43]	0.67
Foley et al. (2013)	0.22 [0.12, 0.31]	0.65
Gott et al. (2017)	0.55 [0.45, 0.65]	0.65
Heaton et al. (2008)	0.35 [0.29, 0.42]	0.67
Hestad et al. (2019)	0.37 [0.31, 0.42]	0.68
Jumare et al. (2020)	0.28 [0.25, 0.31]	0.69
Kabuba et al. (2017)	0.35 [0.29, 0.40]	0.68
Kalayjian et al. (2014)	0.23 [0.17, 0.28]	0.68
Kallianpur et al. (2016)	0.36 [0.34, 0.39]	0.69
Kamat et al. (2017)	0.33 [0.22, 0.44]	0.63
Kamminga et al. (2017)	0.47 [0.34, 0.60]	0.61
Matchanova et al. (2020)	0.60 [0.52, 0.68]	0.66
Moore et al. (2012)	0.19 [0.14, 0.24]	0.68

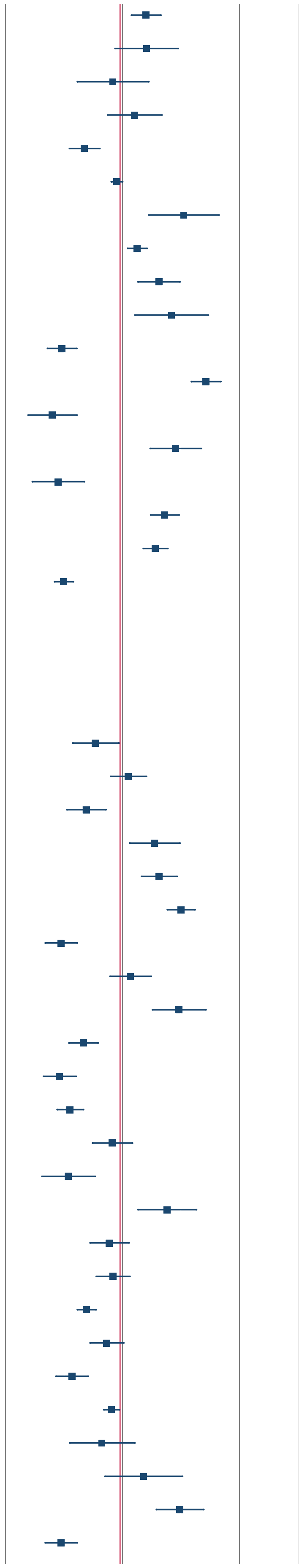

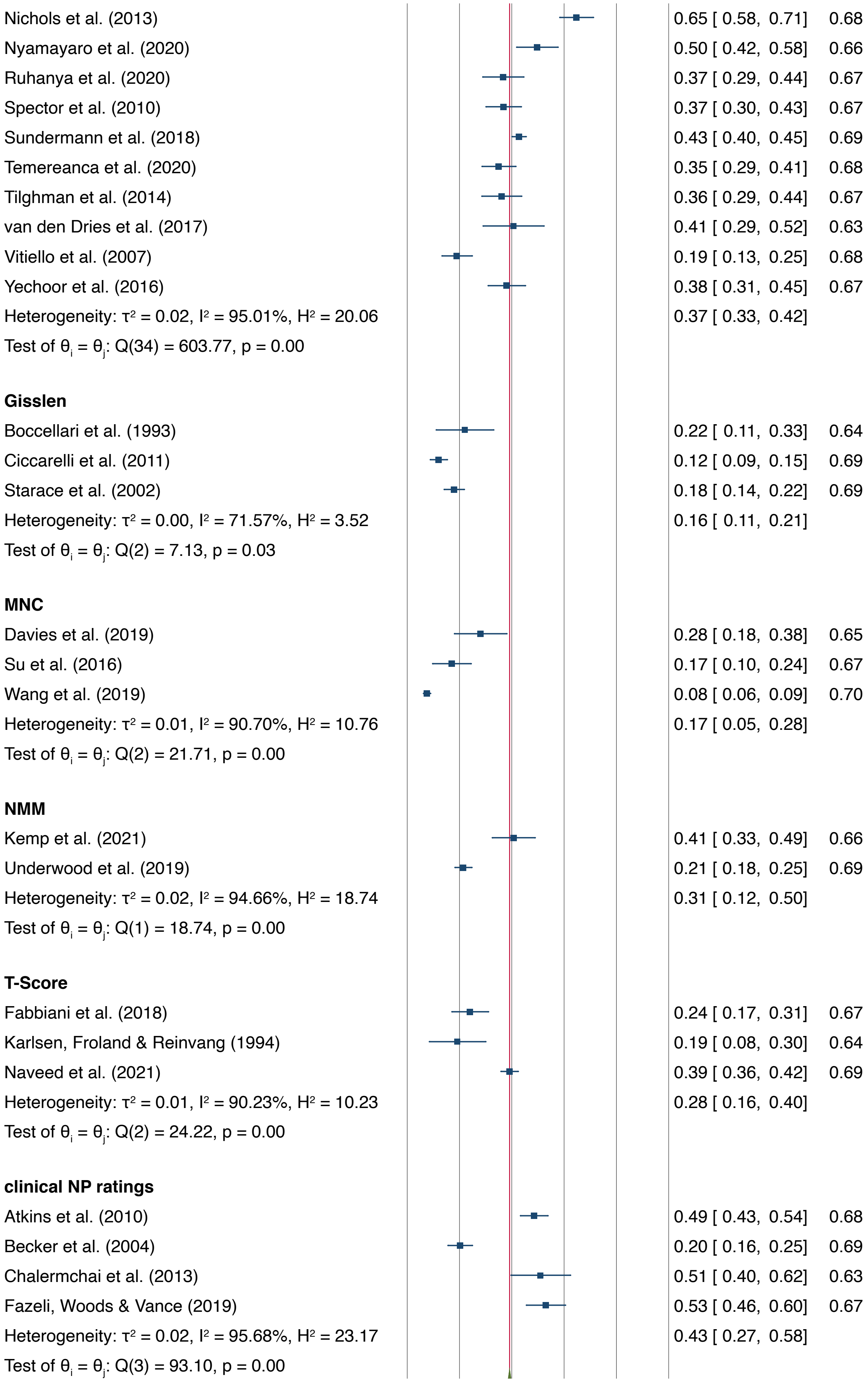
Nichols et al. (2013) 0.65 [0.58, 0.71] 0.68
Nyamayaro et al. (2020) 0.50 [0.42, 0.58] 0.66
Ruhanya et al. (2020) 0.37 [0.29, 0.44] 0.67
Spector et al. (2010) 0.37 [0.30, 0.43] 0.67
Sundermann et al. (2018) 0.43 [0.40, 0.45] 0.69
Temereanca et al. (2020) 0.35 [0.29, 0.41] 0.68
Tilghman et al. (2014) 0.36 [0.29, 0.44] 0.67
van den Dries et al. (2017) 0.41 [0.29, 0.52] 0.63
Vitiello et al. (2007) 0.19 [0.13, 0.25] 0.68
Yechoor et al. (2016) 0.38 [0.31, 0.45] 0.67
Heterogeneity: τ^2 = 0.02, I^2 = 95.01%, H^2 = 20.06 0.37 [0.33, 0.42]
Test of $\theta_i = \theta_j$: Q(34) = 603.77, p = 0.00
Gisslen
Boccellari et al. (1993) 0.22 [0.11, 0.33] 0.64
Ciccarelli et al. (2011) 0.12 [0.09, 0.15] 0.69
Starace et al. (2002) 0.18 [0.14, 0.22] 0.69
Heterogeneity: τ^2 = 0.00, I^2 = 71.57%, H^2 = 3.52 0.16 [0.11, 0.21]
Test of $\theta_i = \theta_j$: Q(2) = 7.13, p = 0.03
MNC
Davies et al. (2019) 0.28 [0.18, 0.38] 0.65
Su et al. (2016) 0.17 [0.10, 0.24] 0.67
Wang et al. (2019) 0.08 [0.06, 0.09] 0.70
Heterogeneity: τ^2 = 0.01, I^2 = 90.70%, H^2 = 10.76 0.17 [0.05, 0.28]
Test of $\theta_i = \theta_j$: Q(2) = 21.71, p = 0.00
NMM
Kemp et al. (2021) 0.41 [0.33, 0.49] 0.66
Underwood et al. (2019) 0.21 [0.18, 0.25] 0.69
Heterogeneity: τ^2 = 0.02, I^2 = 94.66%, H^2 = 18.74 0.31 [0.12, 0.50]
Test of $\theta_i = \theta_j$: Q(1) = 18.74, p = 0.00
T-Score
Fabbiani et al. (2018) 0.24 [0.17, 0.31] 0.67
Karlsen, Froland & Reinvang (1994) 0.19 [0.08, 0.30] 0.64
Naveed et al. (2021) 0.39 [0.36, 0.42] 0.69
Heterogeneity: τ^2 = 0.01, I^2 = 90.23%, H^2 = 10.23 0.28 [0.16, 0.40]
Test of $\theta_i = \theta_j$: Q(2) = 24.22, p = 0.00
clinical NP ratings
Atkins et al. (2010) 0.49 [0.43, 0.54] 0.68
Becker et al. (2004) 0.20 [0.16, 0.25] 0.69
Chalermchai et al. (2013) 0.51 [0.40, 0.62] 0.63
Fazeli, Woods & Vance (2019) 0.53 [0.46, 0.60] 0.67
Heterogeneity: τ^2 = 0.02, I^2 = 95.68%, H^2 = 23.17 0.43 [0.27, 0.58]
Test of $\theta_i = \theta_j$: Q(3) = 93.10, p = 0.00

**Overall**	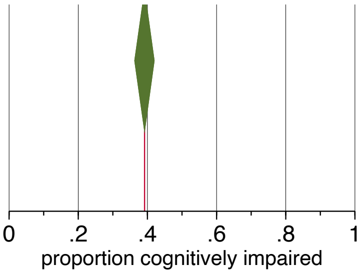	0.39 [0.36, 0.42]
Heterogeneity: τ^2 = 0.03, I^2 = 97.82%, H^2 = 45.86		
Test of $\theta_i = \theta_j$: Q(149) = 7311.83, p = 0.00		
Test of group differences: $Q_b(8)$ = 80.91, p = 0.00		

Random-effects REML model

Supplementary Figure 11: Forest plot including studies categorised as a screening method.

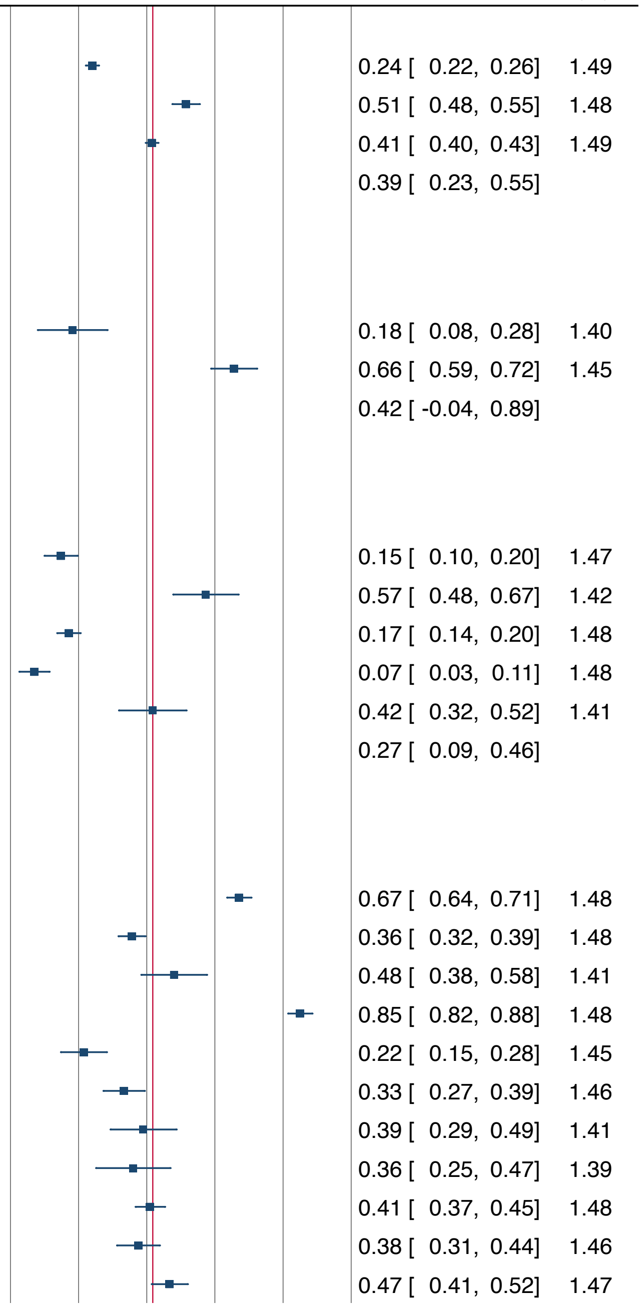

Study	Effect Size with 95% CI	Weight (%)
**BNCS**		
Kalayjian et al. (2019)	0.24 [0.22, 0.26]	1.49
McNamara et al. (2017)	0.51 [0.48, 0.55]	1.48
Robertson et al. (2019)	0.41 [0.40, 0.43]	1.49
Heterogeneity: τ^2 = 0.02, I^2 = 99.29%, H^2 = 140.28	0.39 [0.23, 0.55]	
Test of $\theta_i = \theta_j$: Q(2) = 246.50, p = 0.00		
**CSID**		
Joska et al. (2016)	0.18 [0.08, 0.28]	1.40
Odiase, Ogunrin & Ogunniyi (2006)	0.66 [0.59, 0.72]	1.45
Heterogeneity: τ^2 = 0.11, I^2 = 98.27%, H^2 = 57.91	0.42 [-0.04, 0.89]	
Test of $\theta_i = \theta_j$: Q(1) = 57.91, p = 0.00		
**HDS**		
Banerjee, McIntosh & Ironson (2019)	0.15 [0.10, 0.20]	1.47
Fialho et al. (2013)	0.57 [0.48, 0.67]	1.42
Ganasen et al. (2008)	0.17 [0.14, 0.20]	1.48
Gibbie et al. (2006)	0.07 [0.03, 0.11]	1.48
Jevtovic et al. (2009)	0.42 [0.32, 0.52]	1.41
Heterogeneity: τ^2 = 0.04, I^2 = 98.31%, H^2 = 59.00	0.27 [0.09, 0.46]	
Test of $\theta_i = \theta_j$: Q(4) = 112.98, p = 0.00		
**IHDS**		
Animut et al. (2019)	0.67 [0.64, 0.71]	1.48
Araya et al. (2020)	0.36 [0.32, 0.39]	1.48
Asiedu, Kretchy & Asampong (2020)	0.48 [0.38, 0.58]	1.41
Atashili et al. (2013)	0.85 [0.82, 0.88]	1.48
Barber et al. (2017)	0.22 [0.15, 0.28]	1.45
Belete, Medfu & Yemiyamrew (2017)	0.33 [0.27, 0.39]	1.46
Chan et al. (2012)	0.39 [0.29, 0.49]	1.41
Cook et al. (2014)	0.36 [0.25, 0.47]	1.39
Cross et al. (2013)	0.41 [0.37, 0.45]	1.48
Dang et al. (2015)	0.38 [0.31, 0.44]	1.46
Ding et al. (2017)	0.47 [0.41, 0.52]	1.47

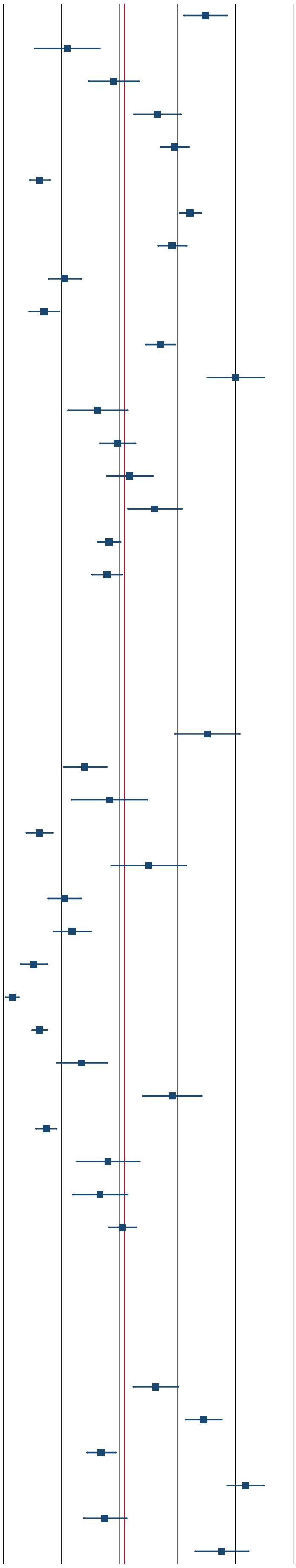

Study	Effect size [95% CI]	Weight (%)
Duarte et al. (2020)	0.70 [0.62, 0.77]	1.45
Holguin et al. (2011)	0.22 [0.11, 0.33]	1.39
Lawler et al. (2010)	0.38 [0.29, 0.47]	1.43
Mogambery et al. (2017)	0.53 [0.45, 0.61]	1.44
Molinaro et al. (2020)	0.59 [0.54, 0.64]	1.47
Mugendi et al. (2019)	0.12 [0.09, 0.16]	1.48
Nakku, Kinyada & Hoskins (2013)	0.64 [0.61, 0.68]	1.48
Namagga et al. (2019)	0.58 [0.53, 0.63]	1.47
Njamnshi et al. (2008)	0.21 [0.16, 0.27]	1.47
Patel et al. (2010)	0.14 [0.09, 0.19]	1.47
Pinheiro et al. (2016)	0.54 [0.49, 0.59]	1.47
Robbins et al. (2011)	0.80 [0.70, 0.90]	1.41
Saini & Barar (2016)	0.32 [0.22, 0.43]	1.40
Salahuddin et al. (2020)	0.39 [0.33, 0.45]	1.46
Tomita et al. (2019)	0.44 [0.36, 0.51]	1.44
Troncoso & de Oliveira Contero (2015)	0.52 [0.43, 0.61]	1.42
Tsegaw et al. (2017)	0.36 [0.33, 0.40]	1.48
Yideg et al. (2019)	0.36 [0.31, 0.41]	1.47
Heterogeneity: $\tau^2 = 0.03$, $I^2 = 97.70\%$, $H^2 = 43.56$	0.44 [0.37, 0.51]	
Test of $\theta_i = \theta_j$: $Q(28) = 1627.59$, $p = 0.00$		
**MMSE**		
Akena et al. (2010)	0.70 [0.59, 0.81]	1.39
Cruz & Ramos (2015)	0.28 [0.21, 0.35]	1.45
Filho & de Melo (2012)	0.37 [0.23, 0.50]	1.35
Imam (2007)	0.12 [0.08, 0.17]	1.48
Kalungwana et al. (2014)	0.50 [0.37, 0.63]	1.36
Kumar et al. (2019)	0.21 [0.15, 0.27]	1.47
Kupprat et al. (2017)	0.24 [0.17, 0.30]	1.46
Madan, Singh & Golechha (1997)	0.10 [0.06, 0.15]	1.47
Oshinaike et al. (2012)	0.03 [0.01, 0.05]	1.49
Qiao et al. (2019)	0.12 [0.10, 0.15]	1.49
Sereia et al. (2012)	0.27 [0.18, 0.36]	1.43
Suarez et al. (2001)	0.58 [0.48, 0.68]	1.41
Tamargo et al. (2021)	0.15 [0.11, 0.18]	1.48
Tremont-Lukats, Teixeira & Hernandez (1999)	0.36 [0.25, 0.47]	1.39
Widyadharma et al. (2017)	0.33 [0.24, 0.43]	1.42
Wubetu, Asefa & Gebregiorgis (2021)	0.41 [0.36, 0.46]	1.47
Heterogeneity: $\tau^2 = 0.03$, $I^2 = 98.01\%$, $H^2 = 50.35$	0.29 [0.20, 0.38]	
Test of $\theta_i = \theta_j$: $Q(15) = 496.86$, $p = 0.00$		
**MoCA**		
Agarwal et al. (2020)	0.52 [0.45, 0.60]	1.44
Awori et al. (2018)	0.69 [0.63, 0.75]	1.46
Bourgeois et al. (2020)	0.34 [0.29, 0.39]	1.47
Brito-Marques et al. (2020)	0.83 [0.77, 0.90]	1.46
Derry et al. (2020)	0.35 [0.28, 0.42]	1.45
Fitri, Rambe & Fitri (2018)	0.75 [0.66, 0.84]	1.42

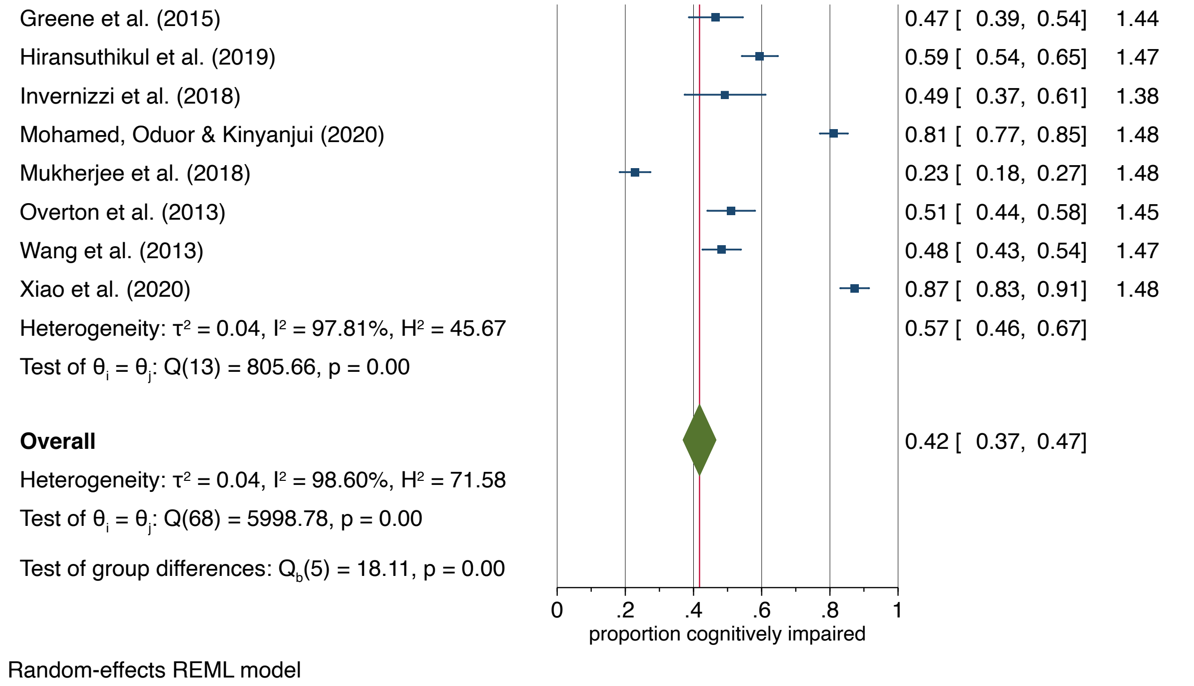

Supplementary Figure 12: Forest plot including studies categorised as other criteria.

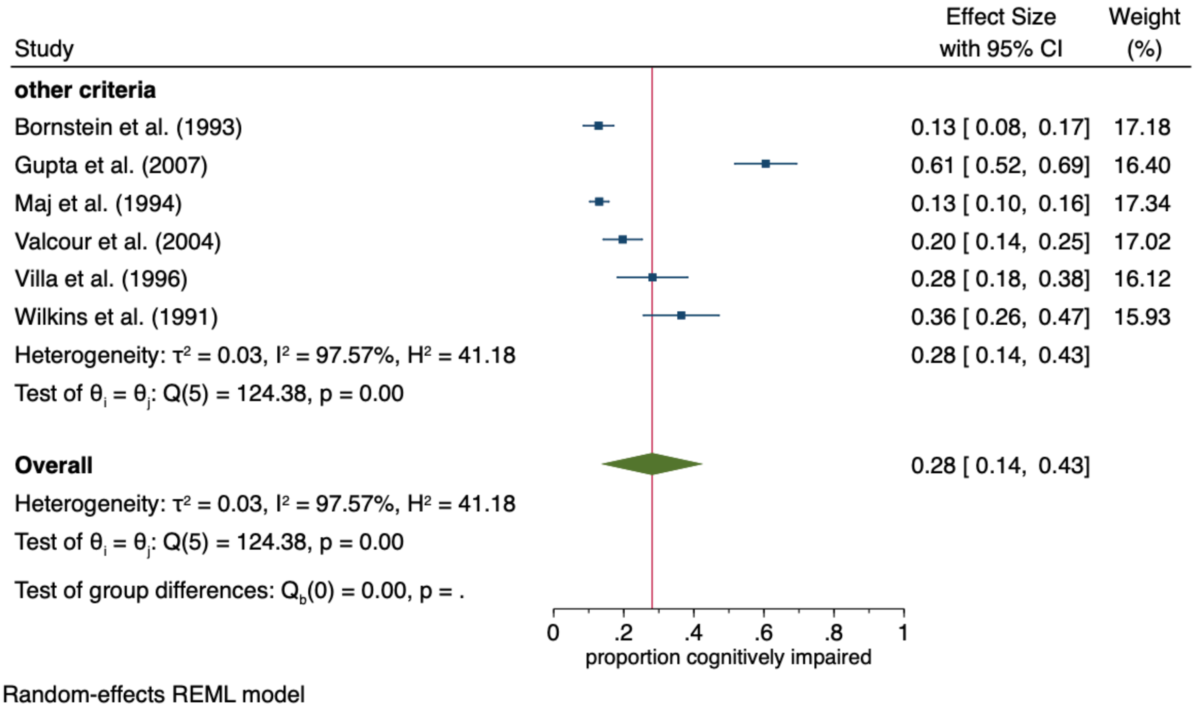

Supplementary Figure 13: Forest plot of study subgroups by income level (country). The subgroup "mixed or unclassified" refers to multi-site studies and studies from Venezuela, which is not classified by The World Bank. Figure made using STATA.

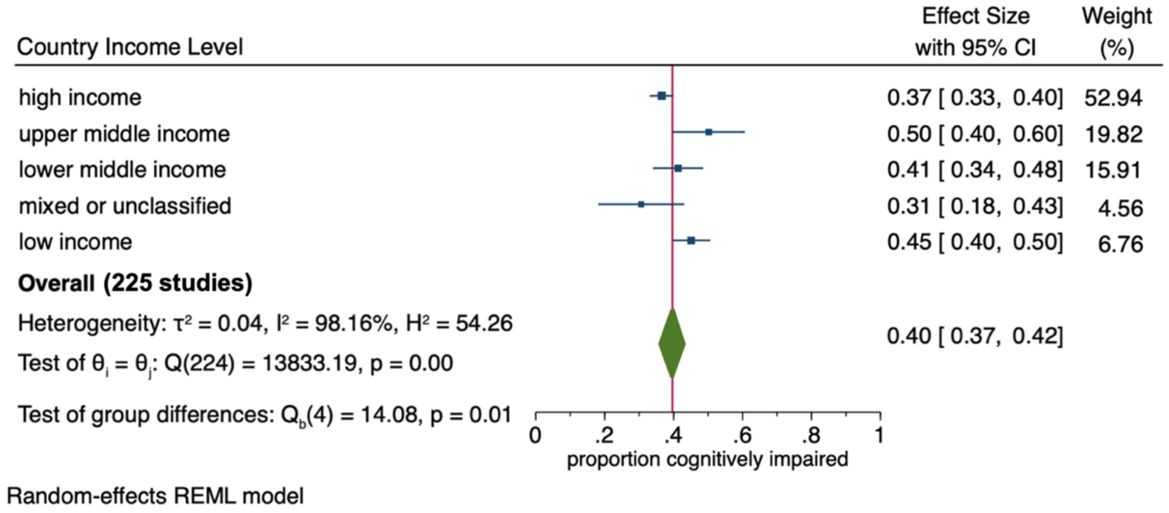

Supplementary Figure 14: Forest plot including studies from high-income countries.

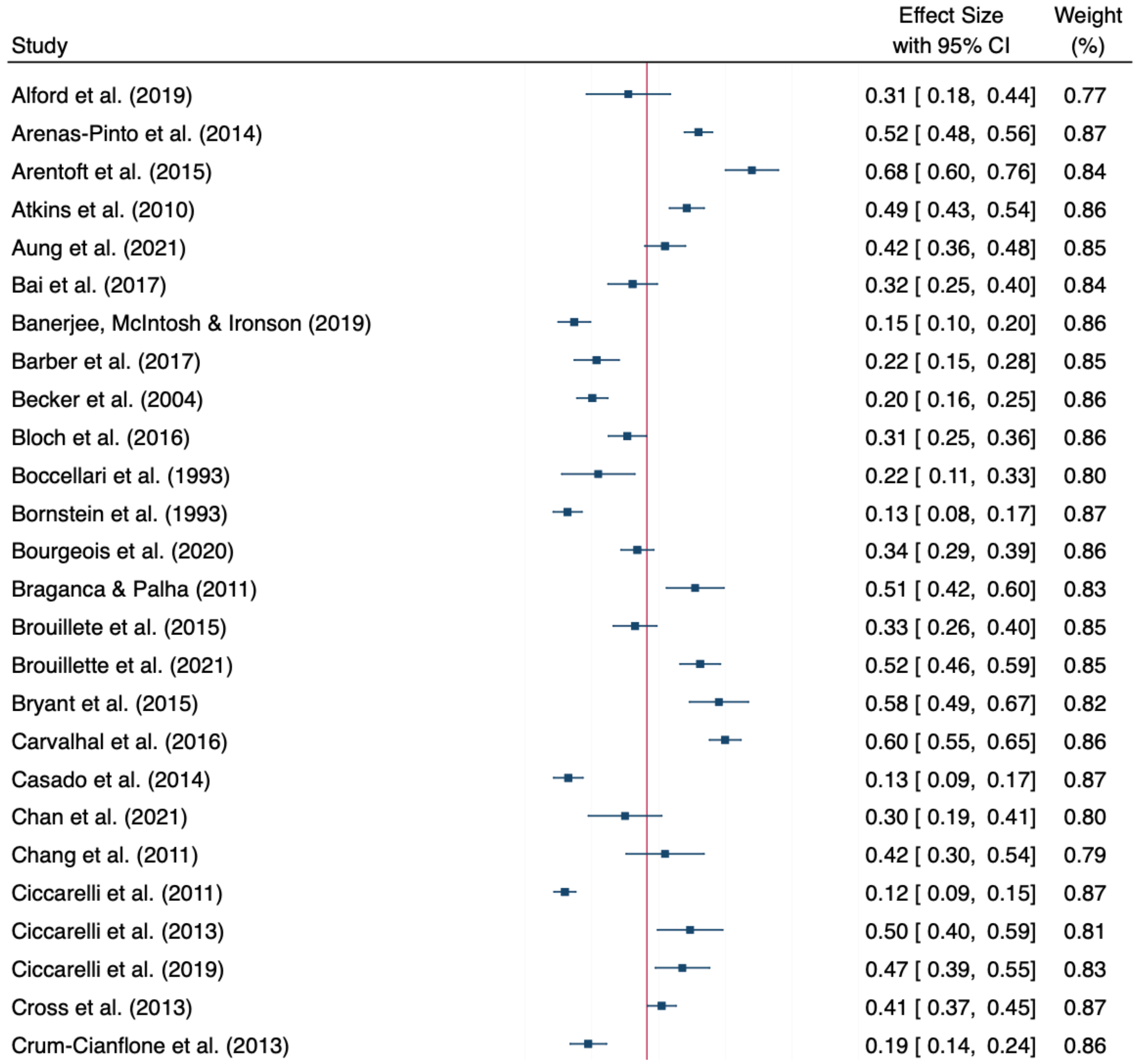

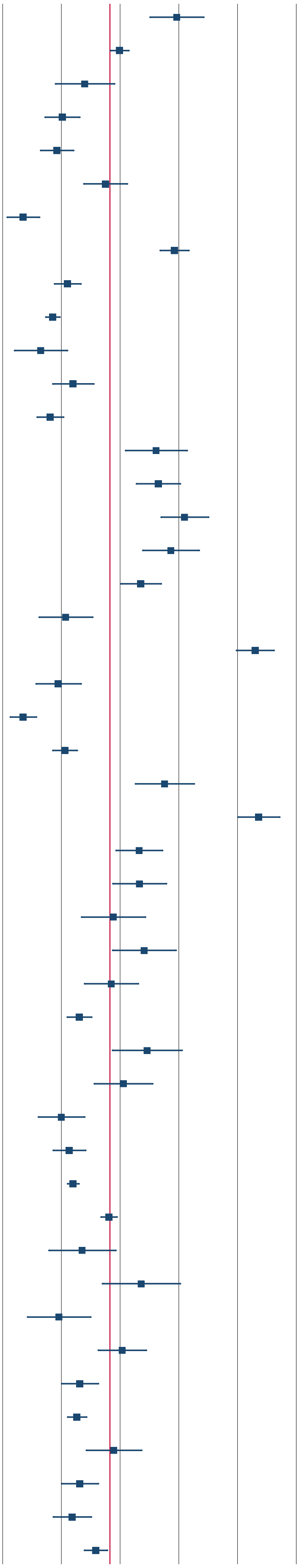

Dampier et al. (2017)	0.59 [0.50, 0.68]	0.82
Darling et al. (2021)	0.40 [0.37, 0.43]	0.87
Davies et al. (2019)	0.28 [0.18, 0.38]	0.81
de Ronchi, Faranca & Berardi (2002)	0.20 [0.14, 0.26]	0.86
Deiss et al. (2019)	0.19 [0.13, 0.24]	0.86
Derry et al. (2020)	0.35 [0.28, 0.42]	0.84
Donne et al. (2020)	0.07 [0.02, 0.12]	0.86
Dufouil et al. (2015)	0.58 [0.54, 0.63]	0.86
Dufour et al. (2013)	0.22 [0.18, 0.27]	0.87
Erlandson et al. (2019)	0.17 [0.15, 0.19]	0.88
Fabbiani et al. (2017)	0.13 [0.04, 0.22]	0.82
Fabbiani et al. (2018)	0.24 [0.17, 0.31]	0.85
Fabbiani et al. (2019)	0.16 [0.12, 0.21]	0.87
Failde-Garrido et al. (2008)	0.52 [0.42, 0.63]	0.80
Fazeli, Woods & Vance (2019)	0.53 [0.46, 0.60]	0.84
Ferrando et al. (2003)	0.62 [0.54, 0.70]	0.83
Fialho et al. (2013)	0.57 [0.48, 0.67]	0.82
Foca et al. (2016)	0.47 [0.40, 0.54]	0.85
Foley et al. (2013)	0.22 [0.12, 0.31]	0.82
Gandhi et al. (2011)	0.86 [0.80, 0.92]	0.85
Garvey, Surendrakumar & Winston (2011)	0.19 [0.11, 0.27]	0.84
Gibbie et al. (2006)	0.07 [0.03, 0.11]	0.87
Gomez et al. (2019)	0.21 [0.17, 0.25]	0.87
Gott et al. (2017)	0.55 [0.45, 0.65]	0.81
Grauer et al. (2015)	0.87 [0.80, 0.94]	0.84
Greene et al. (2015)	0.47 [0.39, 0.54]	0.84
Grima et al. (2012)	0.47 [0.38, 0.56]	0.82
Groff et al. (2020)	0.38 [0.27, 0.49]	0.80
Halman et al. (2014)	0.48 [0.37, 0.59]	0.80
Hanna et al. (2020)	0.37 [0.28, 0.46]	0.82
Imai et al. (2020)	0.26 [0.22, 0.30]	0.87
Invernizzi et al. (2018)	0.49 [0.37, 0.61]	0.79
Janssen et al. (1989)	0.41 [0.31, 0.51]	0.81
Janssen et al. (2015)	0.20 [0.12, 0.28]	0.84
Kalayjian et al. (2014)	0.23 [0.17, 0.28]	0.86
Kalayjian et al. (2019)	0.24 [0.22, 0.26]	0.88
Kallianpur et al. (2016)	0.36 [0.34, 0.39]	0.88
Kamal et al. (2017)	0.27 [0.16, 0.38]	0.79
Kamminga et al. (2017)	0.47 [0.34, 0.60]	0.76
Karlsen, Froland & Reinvang (1994)	0.19 [0.08, 0.30]	0.80
Kemp et al. (2021)	0.41 [0.33, 0.49]	0.83
Kim et al. (2016)	0.26 [0.20, 0.32]	0.85
Kinai et al. (2017)	0.25 [0.22, 0.28]	0.87
Klusman et al. (1991)	0.38 [0.29, 0.47]	0.82
Ku et al. (2014)	0.26 [0.20, 0.32]	0.85
Kupprat et al. (2017)	0.24 [0.17, 0.30]	0.85
Libertone et al. (2014)	0.32 [0.28, 0.36]	0.87

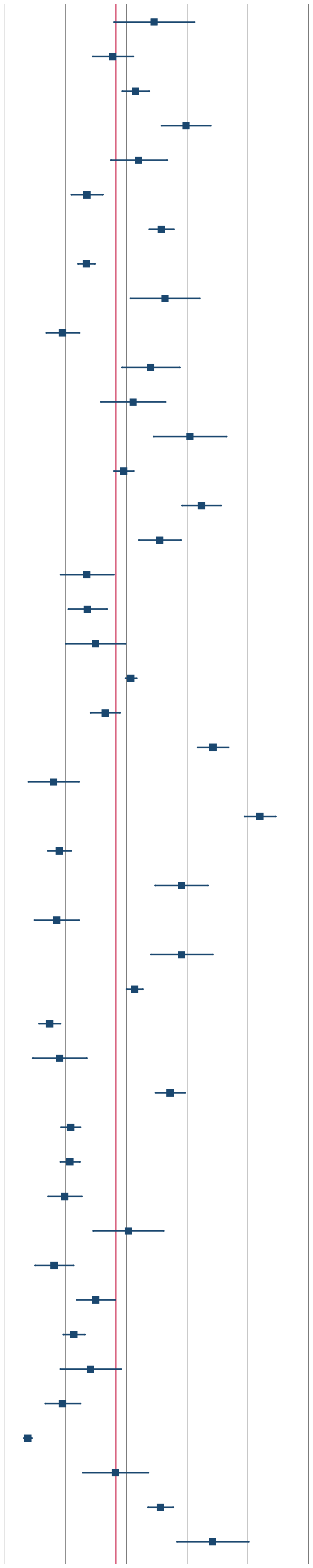

Lu et al. (2014)	0.49 [0.36, 0.62]	0.76
Makinson et al. (2020)	0.35 [0.29, 0.42]	0.85
Marin-Webb et al. (2016)	0.43 [0.39, 0.47]	0.87
Matchanova et al. (2020)	0.60 [0.52, 0.68]	0.83
Mayeux et al. (1993)	0.44 [0.35, 0.53]	0.82
McCutchan et al. (2007)	0.27 [0.22, 0.32]	0.86
McNamara et al. (2017)	0.51 [0.48, 0.55]	0.87
Metral et al. (2020)	0.27 [0.24, 0.30]	0.87
Milanini et al. (2020)	0.53 [0.41, 0.64]	0.79
Moore et al. (2012)	0.19 [0.14, 0.24]	0.86
Munoz-Moreno et al. (2008)	0.48 [0.38, 0.58]	0.82
Munoz-Moreno et al. (2010)	0.42 [0.32, 0.53]	0.80
Munoz-Moreno et al. (2013)	0.61 [0.49, 0.73]	0.78
Naveed et al. (2021)	0.39 [0.36, 0.42]	0.87
Nichols et al. (2013)	0.65 [0.58, 0.71]	0.85
Overton et al. (2013)	0.51 [0.44, 0.58]	0.85
Pereda et al. (2000)	0.27 [0.18, 0.36]	0.83
Perez-Valero et al. (2013)	0.27 [0.21, 0.34]	0.85
Portilla et al. (2019)	0.30 [0.20, 0.40]	0.81
Robertson et al. (2019)	0.41 [0.40, 0.43]	0.88
Sacktor et al. (2014)	0.33 [0.28, 0.38]	0.86
Sevigny et al. (2007)	0.69 [0.63, 0.74]	0.86
Sheppard et al. (2015)	0.16 [0.08, 0.24]	0.83
Simioni et al. (2010)	0.84 [0.79, 0.89]	0.86
Starace et al. (2002)	0.18 [0.14, 0.22]	0.87
Stern et al. (1991)	0.58 [0.49, 0.67]	0.83
Su et al. (2016)	0.17 [0.10, 0.24]	0.84
Suarez et al. (2001)	0.58 [0.48, 0.68]	0.81
Sundermann et al. (2018)	0.43 [0.40, 0.45]	0.88
Tamargo et al. (2021)	0.15 [0.11, 0.18]	0.87
Thiyagarajan et al. (2010)	0.18 [0.09, 0.27]	0.82
Tozzi et al. (2005)	0.54 [0.50, 0.59]	0.86
Trunfio et al. (2018)	0.22 [0.18, 0.25]	0.87
Underwood et al. (2019)	0.21 [0.18, 0.25]	0.87
Valcour et al. (2004)	0.20 [0.14, 0.25]	0.86
van den Dries et al. (2017)	0.41 [0.29, 0.52]	0.79
van Gorp et al. (1999)	0.16 [0.10, 0.23]	0.85
Vassallo et al. (2015)	0.30 [0.24, 0.36]	0.85
Vergori et al. (2019)	0.23 [0.19, 0.26]	0.87
Villa et al. (1996)	0.28 [0.18, 0.38]	0.81
Vitiello et al. (2007)	0.19 [0.13, 0.25]	0.86
Wang et al. (2019)	0.08 [0.06, 0.09]	0.88
Wilkins et al. (1991)	0.36 [0.26, 0.47]	0.80
Winston et al. (2013)	0.51 [0.47, 0.55]	0.87
Wojna et al. (2007)	0.68 [0.57, 0.80]	0.79

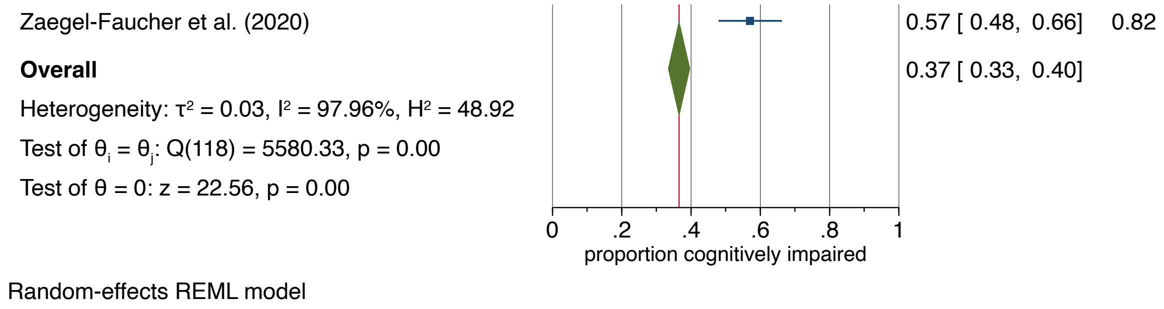

Supplementary Figure 15: Forest plot including studies from upper middle-income countries.

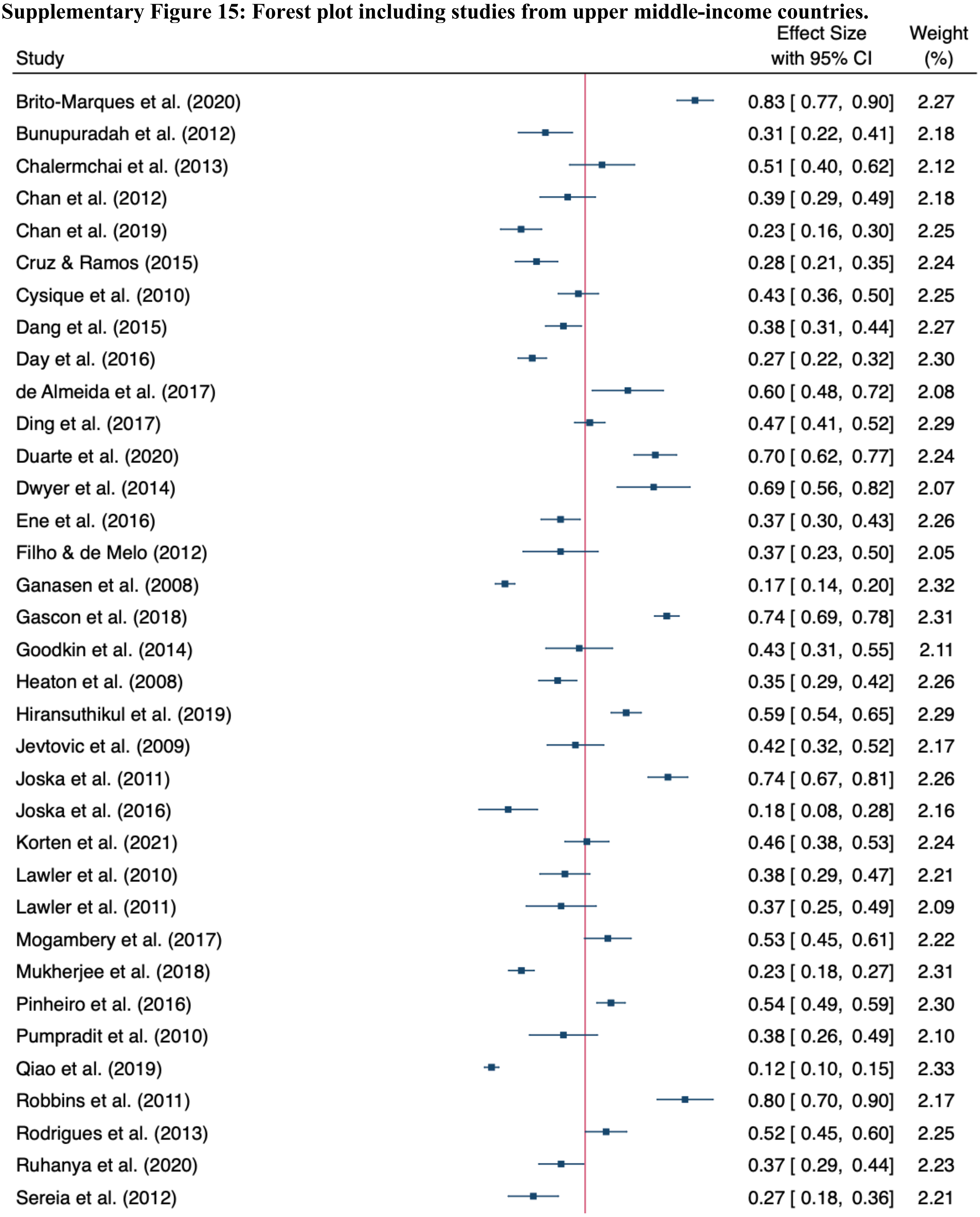

Study	Effect Size with 95% CI	Weight (%)
Spector et al. (2010)	0.37 [0.30, 0.43]	2.26
Temereanca et al. (2020)	0.35 [0.29, 0.41]	2.27
Tomita et al. (2019)	0.44 [0.36, 0.51]	2.23
Troncoso & de Oliveira Contero (2015)	0.52 [0.43, 0.61]	2.19
Valcour et al. (2013)	0.46 [0.33, 0.58]	2.08
Wang et al. (2013)	0.48 [0.43, 0.54]	2.29
Xiao et al. (2020)	0.87 [0.83, 0.91]	2.31
Zamudio-Rodiguez et al. (2018)	0.66 [0.60, 0.72]	2.27
Zhang et al. (2012)	0.37 [0.29, 0.45]	2.22
Zhao et al. (2015)	0.37 [0.31, 0.44]	2.27
**Overall**	0.45 [0.40, 0.50]	

Heterogeneity: $\tau^2 = 0.03$, $I^2 = 96.70\%$, $H^2 = 30.35$

Test of $\theta_i = \theta_j$: $Q(44) = 2161.18$, $p = 0.00$

Test of $\theta = 0$: $z = 16.67$, $p = 0.00$

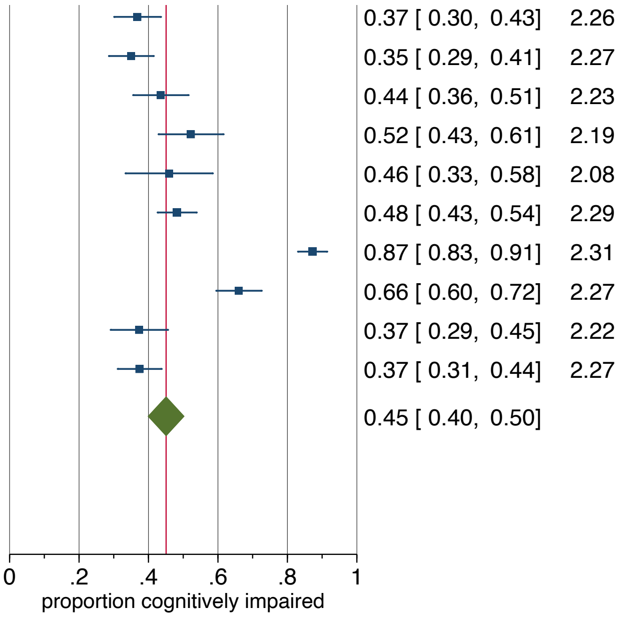

Random-effects REML model

Supplementary Figure 16: Forest plot including studies from lower middle-income countries.

Study	Effect Size with 95% CI	Weight (%)
Agarwal et al. (2020)	0.52 [0.45, 0.60]	2.78
Akolo et al. (2014)	0.31 [0.23, 0.39]	2.78
Asiedu, Kretchy & Asampong (2020)	0.48 [0.38, 0.58]	2.73
Atashili et al. (2013)	0.85 [0.82, 0.88]	2.86
Awori et al. (2018)	0.69 [0.63, 0.75]	2.82
Bharti et al. (2021)	0.28 [0.21, 0.34]	2.81
Cook et al. (2014)	0.36 [0.25, 0.47]	2.69
Cook et al. (2016)	0.57 [0.46, 0.68]	2.69
Elham et al. (2020)	0.51 [0.40, 0.61]	2.72
Estiasari et al. (2015)	0.51 [0.40, 0.62]	2.69
Fitri, Rambe & Fitri (2018)	0.75 [0.66, 0.84]	2.74
Flatt et al. (2021)	0.47 [0.41, 0.53]	2.82
Gupta & Venugopal (2020)	0.48 [0.43, 0.53]	2.84
Gupta et al. (2007)	0.61 [0.52, 0.69]	2.75
Hestad et al. (2019)	0.37 [0.31, 0.42]	2.83
Holguin et al. (2011)	0.22 [0.11, 0.33]	2.69
Imam (2007)	0.12 [0.08, 0.17]	2.84
Jumare et al. (2019)	0.24 [0.18, 0.30]	2.82
Kabuba et al. (2017)	0.35 [0.29, 0.40]	2.83
Kalungwana et al. (2014)	0.50 [0.37, 0.63]	2.62
Kamat et al. (2017)	0.33 [0.22, 0.44]	2.68
Kumar et al. (2019)	0.21 [0.15, 0.27]	2.83
Madan, Singh & Golechha (1997)	0.10 [0.06, 0.15]	2.84
Mohamed, Oduor & Kinyanjui (2020)	0.81 [0.77, 0.85]	2.85
Mugendi et al. (2019)	0.12 [0.09, 0.16]	2.86

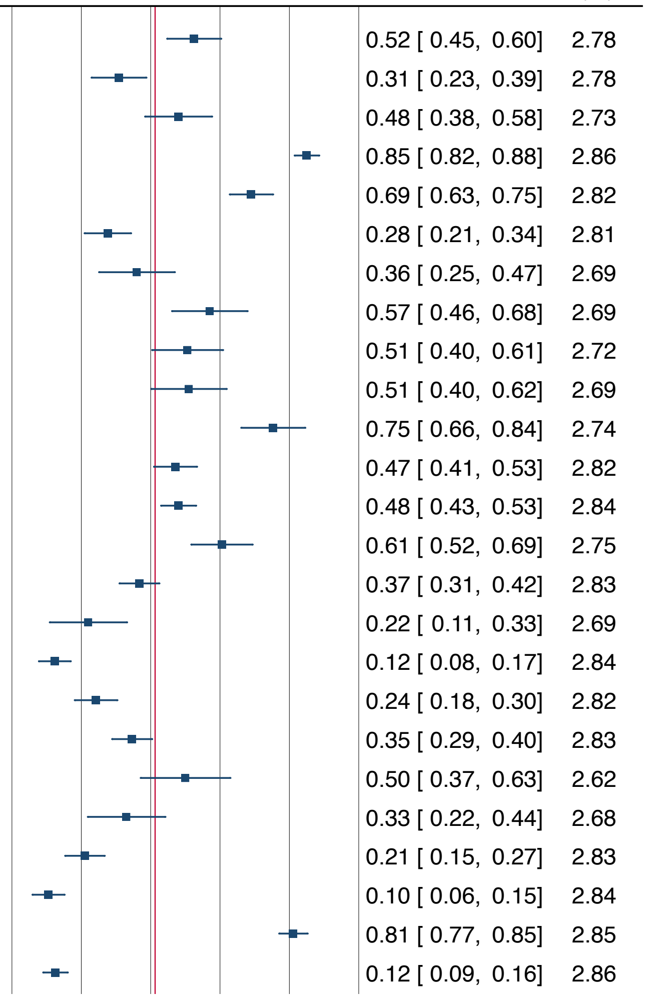

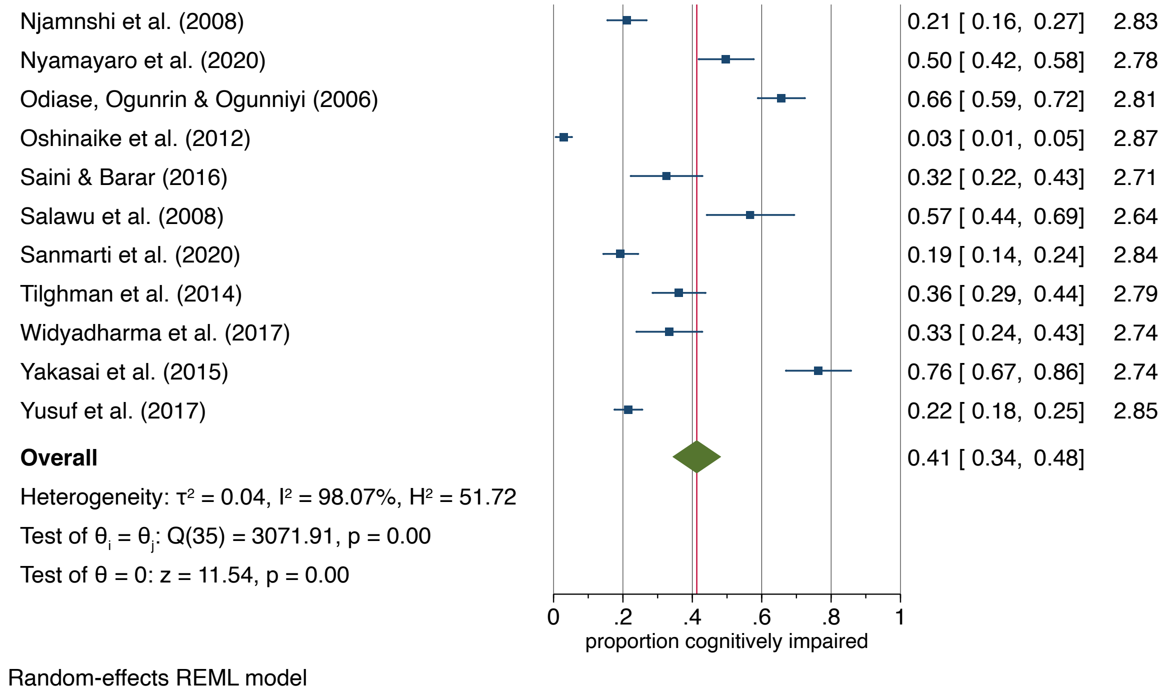

Supplementary Figure 17: Forest plot including studies from low-income countries.

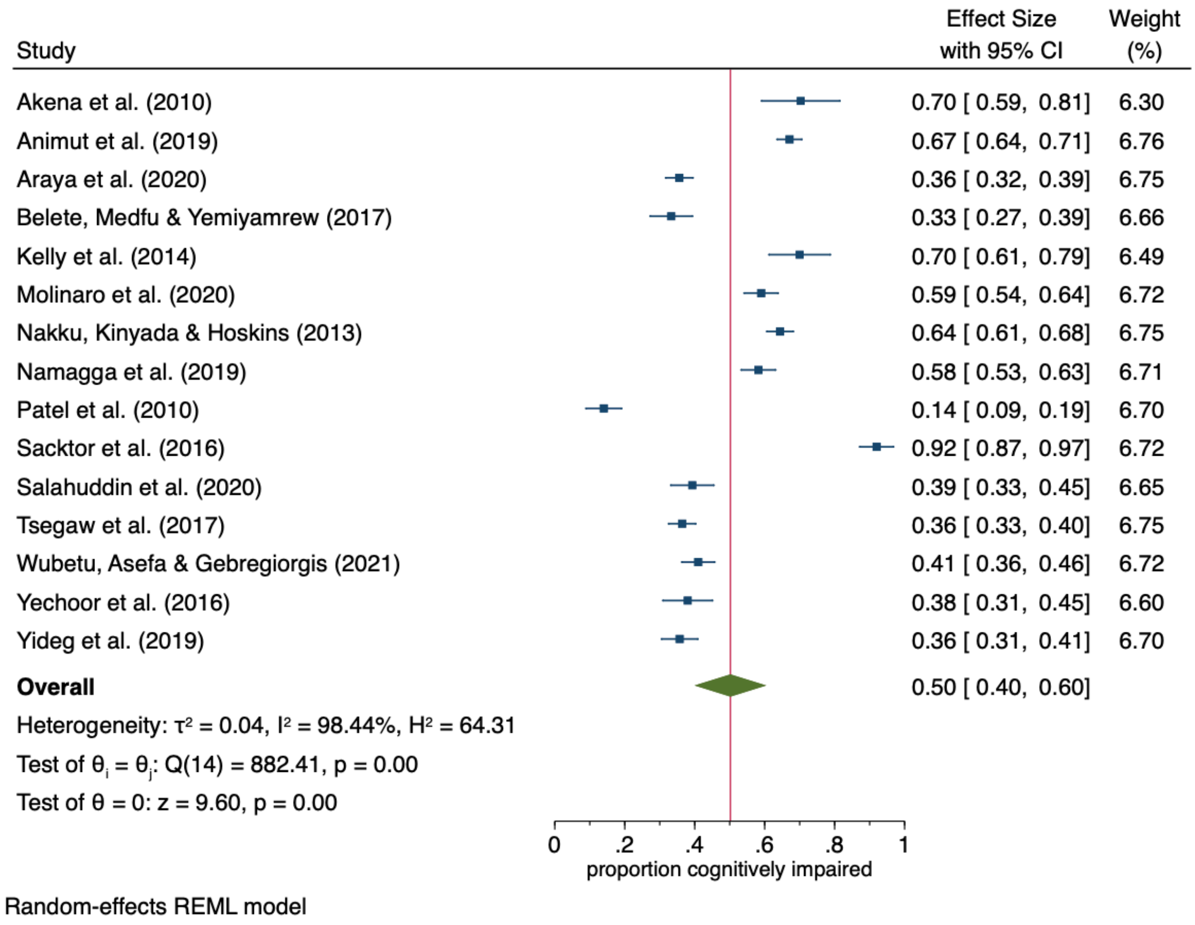

Supplementary Figure 18: Forest plot including studies from mixed or unclassified countries.

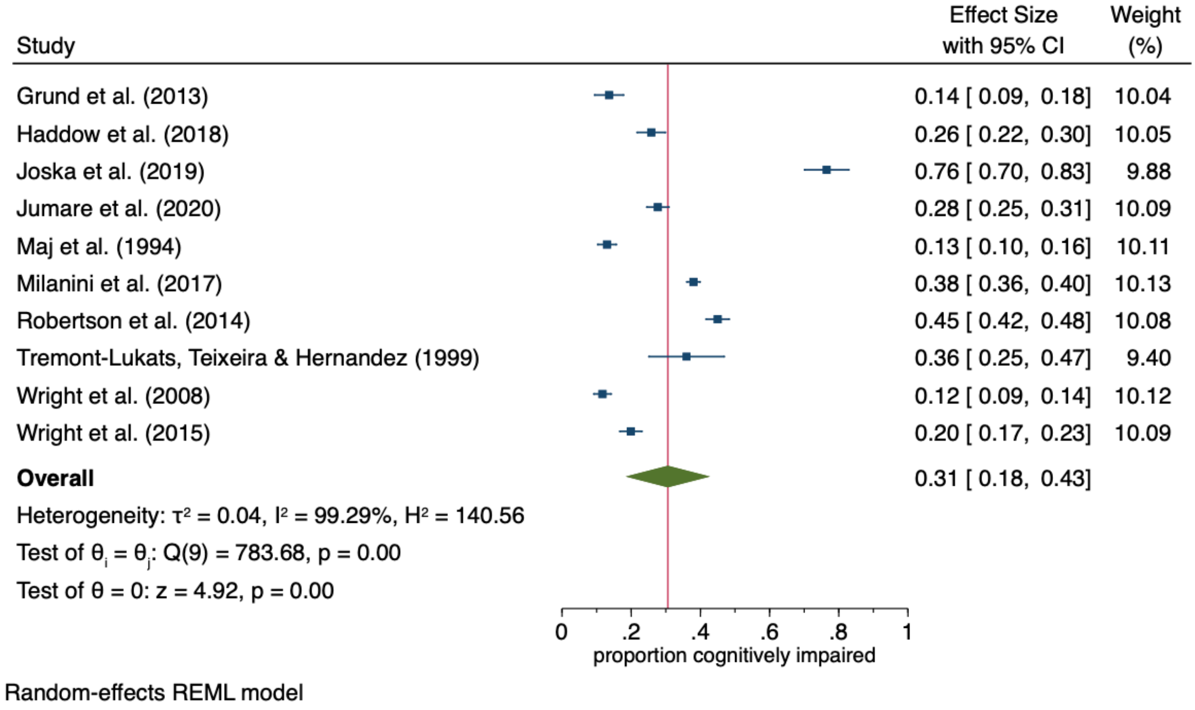

Supplementary Figure 19: Forest plot of study subgroups by study exclusion criteria. Figure made using STATA.

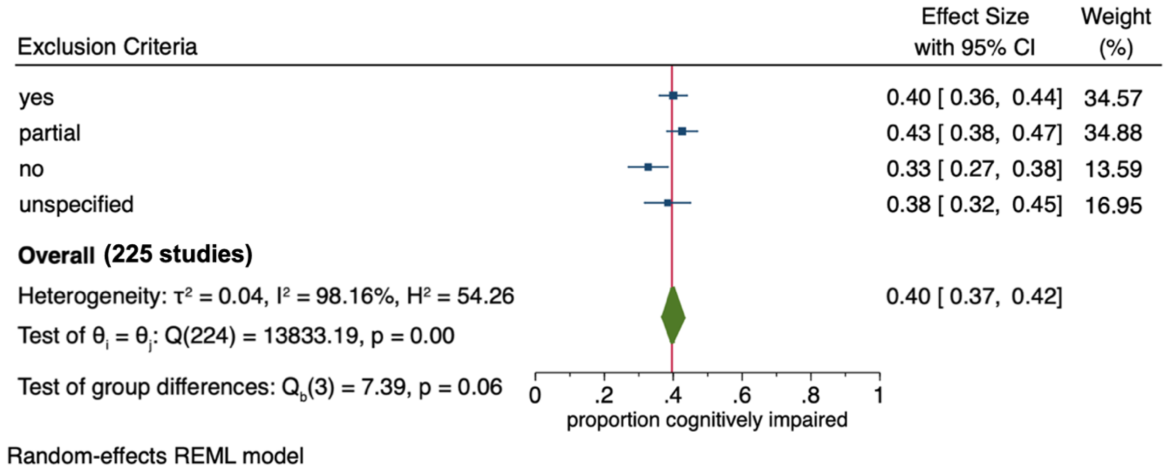

Supplementary Figure 20: Forest plot including studies with exclusion criteria.

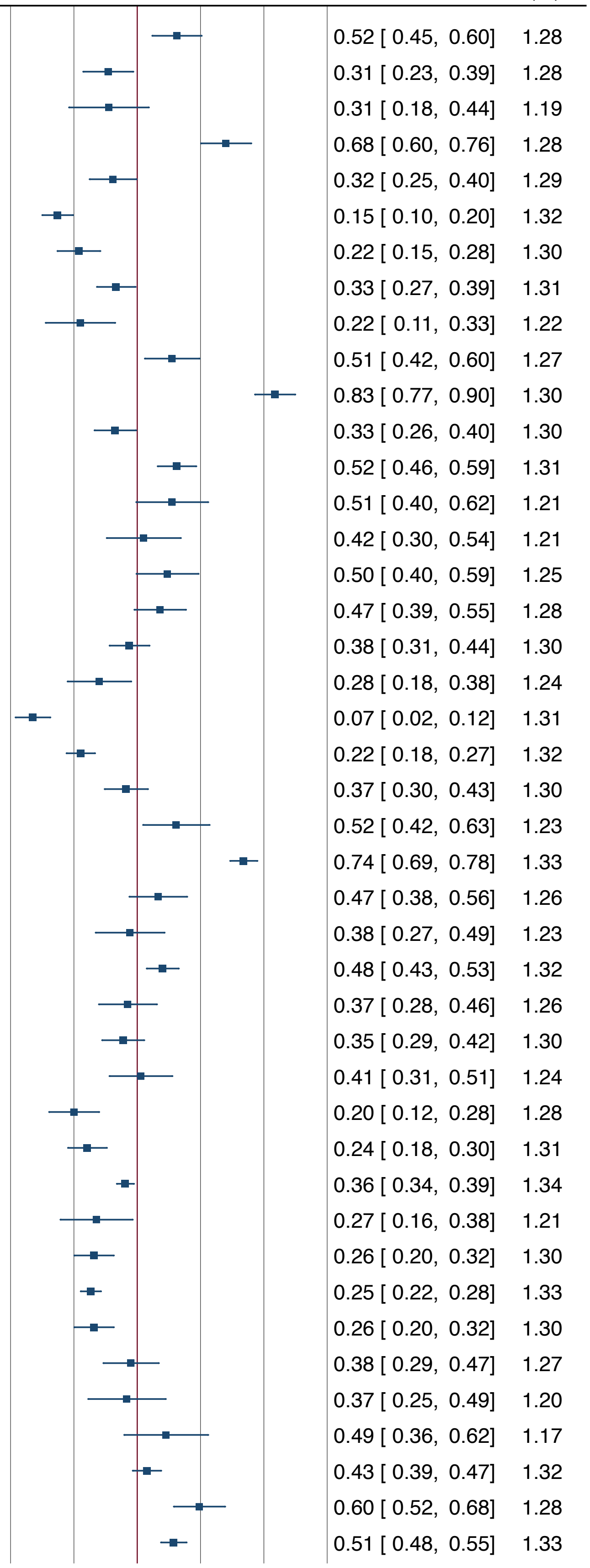

Study	Effect Size with 95% CI	Weight (%)
Agarwal et al. (2020)	0.52 [0.45, 0.60]	1.28
Akolo et al. (2014)	0.31 [0.23, 0.39]	1.28
Alford et al. (2019)	0.31 [0.18, 0.44]	1.19
Arentoft et al. (2015)	0.68 [0.60, 0.76]	1.28
Bai et al. (2017)	0.32 [0.25, 0.40]	1.29
Banerjee, McIntosh & Ironson (2019)	0.15 [0.10, 0.20]	1.32
Barber et al. (2017)	0.22 [0.15, 0.28]	1.30
Belete, Medfu & Yemiyamrew (2017)	0.33 [0.27, 0.39]	1.31
Boccellari et al. (1993)	0.22 [0.11, 0.33]	1.22
Braganca & Palha (2011)	0.51 [0.42, 0.60]	1.27
Brito-Marques et al. (2020)	0.83 [0.77, 0.90]	1.30
Brouillete et al. (2015)	0.33 [0.26, 0.40]	1.30
Brouillette et al. (2021)	0.52 [0.46, 0.59]	1.31
Chalermchai et al. (2013)	0.51 [0.40, 0.62]	1.21
Chang et al. (2011)	0.42 [0.30, 0.54]	1.21
Ciccarelli et al. (2013)	0.50 [0.40, 0.59]	1.25
Ciccarelli et al. (2019)	0.47 [0.39, 0.55]	1.28
Dang et al. (2015)	0.38 [0.31, 0.44]	1.30
Davies et al. (2019)	0.28 [0.18, 0.38]	1.24
Donne et al. (2020)	0.07 [0.02, 0.12]	1.31
Dufour et al. (2013)	0.22 [0.18, 0.27]	1.32
Ene et al. (2016)	0.37 [0.30, 0.43]	1.30
Failde-Garrido et al. (2008)	0.52 [0.42, 0.63]	1.23
Gascon et al. (2018)	0.74 [0.69, 0.78]	1.33
Grima et al. (2012)	0.47 [0.38, 0.56]	1.26
Groff et al. (2020)	0.38 [0.27, 0.49]	1.23
Gupta & Venugopal (2020)	0.48 [0.43, 0.53]	1.32
Hanna et al. (2020)	0.37 [0.28, 0.46]	1.26
Heaton et al. (2008)	0.35 [0.29, 0.42]	1.30
Janssen et al. (1989)	0.41 [0.31, 0.51]	1.24
Janssen et al. (2015)	0.20 [0.12, 0.28]	1.28
Jumare et al. (2019)	0.24 [0.18, 0.30]	1.31
Kallianpur et al. (2016)	0.36 [0.34, 0.39]	1.34
Kamal et al. (2017)	0.27 [0.16, 0.38]	1.21
Kim et al. (2016)	0.26 [0.20, 0.32]	1.30
Kinai et al. (2017)	0.25 [0.22, 0.28]	1.33
Ku et al. (2014)	0.26 [0.20, 0.32]	1.30
Lawler et al. (2010)	0.38 [0.29, 0.47]	1.27
Lawler et al. (2011)	0.37 [0.25, 0.49]	1.20
Lu et al. (2014)	0.49 [0.36, 0.62]	1.17
Marin-Webb et al. (2016)	0.43 [0.39, 0.47]	1.32
Matchanova et al. (2020)	0.60 [0.52, 0.68]	1.28
McNamara et al. (2017)	0.51 [0.48, 0.55]	1.33

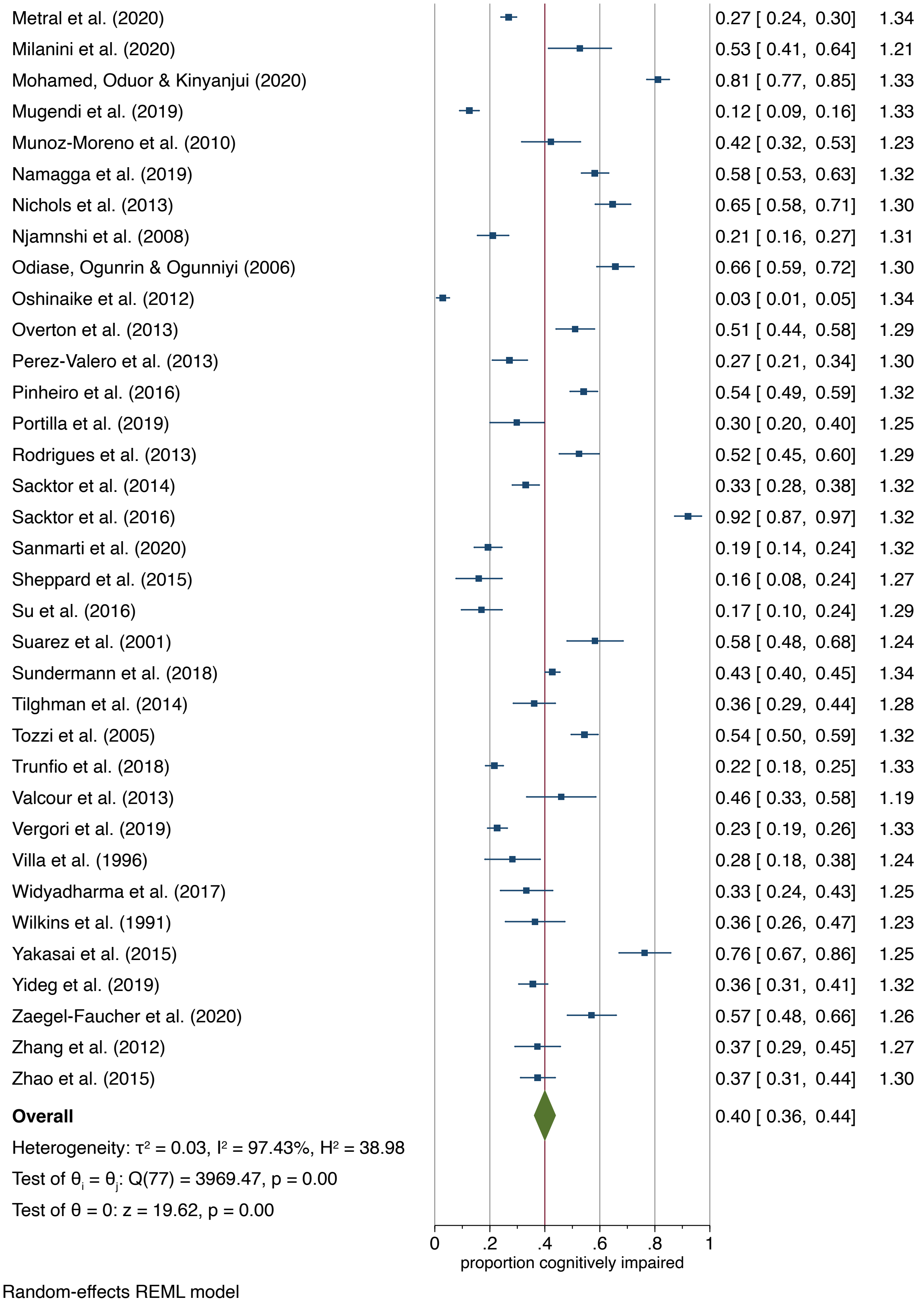
Metral et al. (2020) 0.27 [0.24, 0.30] 1.34
Milanini et al. (2020) 0.53 [0.41, 0.64] 1.21
Mohamed, Oduor & Kinyanjui (2020) 0.81 [0.77, 0.85] 1.33
Mugendi et al. (2019) 0.12 [0.09, 0.16] 1.33
Munoz-Moreno et al. (2010) 0.42 [0.32, 0.53] 1.23
Namagga et al. (2019) 0.58 [0.53, 0.63] 1.32
Nichols et al. (2013) 0.65 [0.58, 0.71] 1.30
Njamnshi et al. (2008) 0.21 [0.16, 0.27] 1.31
Odiase, Ogunrin & Ogunniyi (2006) 0.66 [0.59, 0.72] 1.30
Oshinaike et al. (2012) 0.03 [0.01, 0.05] 1.34
Overton et al. (2013) 0.51 [0.44, 0.58] 1.29
Perez-Valero et al. (2013) 0.27 [0.21, 0.34] 1.30
Pinheiro et al. (2016) 0.54 [0.49, 0.59] 1.32
Portilla et al. (2019) 0.30 [0.20, 0.40] 1.25
Rodrigues et al. (2013) 0.52 [0.45, 0.60] 1.29
Sacktor et al. (2014) 0.33 [0.28, 0.38] 1.32
Sacktor et al. (2016) 0.92 [0.87, 0.97] 1.32
Sanmarti et al. (2020) 0.19 [0.14, 0.24] 1.32
Sheppard et al. (2015) 0.16 [0.08, 0.24] 1.27
Su et al. (2016) 0.17 [0.10, 0.24] 1.29
Suarez et al. (2001) 0.58 [0.48, 0.68] 1.24
Sundermann et al. (2018) 0.43 [0.40, 0.45] 1.34
Tilghman et al. (2014) 0.36 [0.29, 0.44] 1.28
Tozzi et al. (2005) 0.54 [0.50, 0.59] 1.32
Trunfio et al. (2018) 0.22 [0.18, 0.25] 1.33
Valcour et al. (2013) 0.46 [0.33, 0.58] 1.19
Vergori et al. (2019) 0.23 [0.19, 0.26] 1.33
Villa et al. (1996) 0.28 [0.18, 0.38] 1.24
Widyadharma et al. (2017) 0.33 [0.24, 0.43] 1.25
Wilkins et al. (1991) 0.36 [0.26, 0.47] 1.23
Yakasai et al. (2015) 0.76 [0.67, 0.86] 1.25
Yideg et al. (2019) 0.36 [0.31, 0.41] 1.32
Zaegel-Faucher et al. (2020) 0.57 [0.48, 0.66] 1.26
Zhang et al. (2012) 0.37 [0.29, 0.45] 1.27
Zhao et al. (2015) 0.37 [0.31, 0.44] 1.30
Overall 0.40 [0.36, 0.44]
Heterogeneity: $\tau^2 = 0.03$, $I^2 = 97.43\%$, $H^2 = 38.98$
Test of $\theta_i = \theta_j$: Q(77) = 3969.47, p = 0.00
Test of $\theta = 0$: z = 19.62, p = 0.00
0 .2 .4 .6 .8 1
proportion cognitively impaired
Random-effects REML model

Supplementary Figure 21: Forest plot including studies with partial criteria.

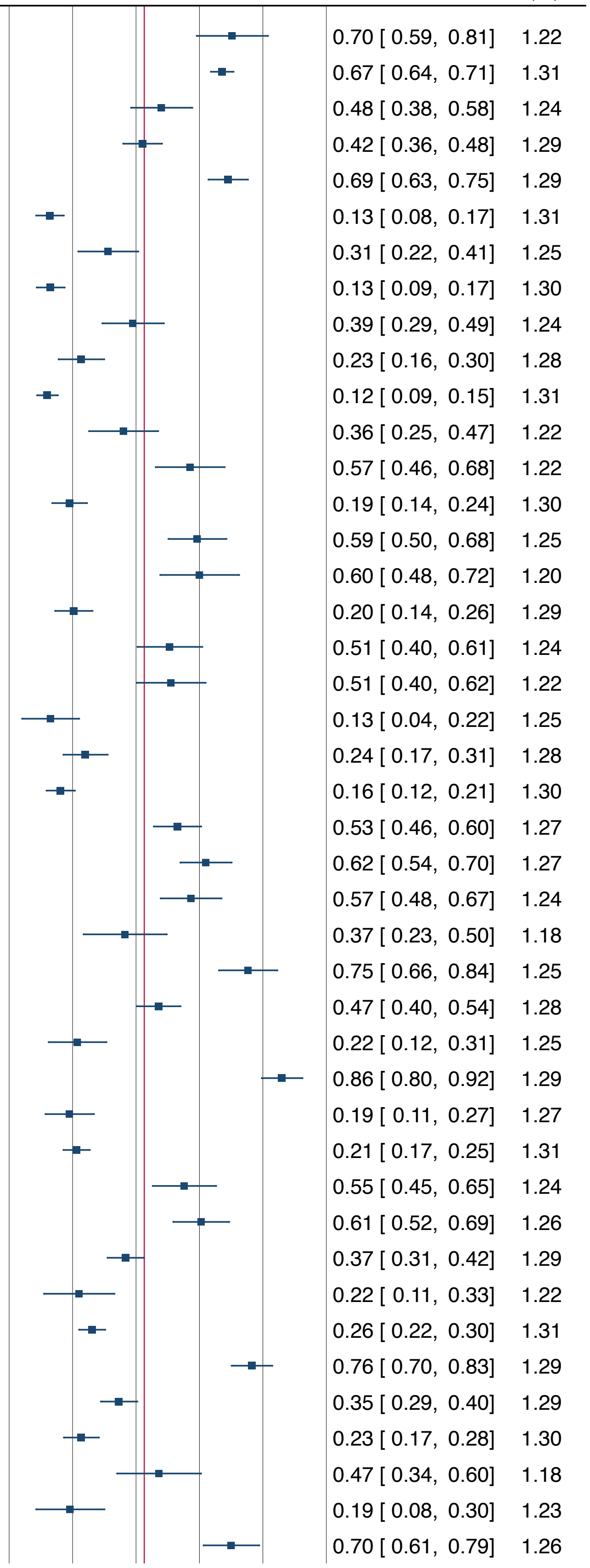

Study	Effect Size with 95% CI	Weight (%)
Akena et al. (2010)	0.70 [0.59, 0.81]	1.22
Animut et al. (2019)	0.67 [0.64, 0.71]	1.31
Asiedu, Kretchy & Asampong (2020)	0.48 [0.38, 0.58]	1.24
Aung et al. (2021)	0.42 [0.36, 0.48]	1.29
Awori et al. (2018)	0.69 [0.63, 0.75]	1.29
Bornstein et al. (1993)	0.13 [0.08, 0.17]	1.31
Bunupuradah et al. (2012)	0.31 [0.22, 0.41]	1.25
Casado et al. (2014)	0.13 [0.09, 0.17]	1.30
Chan et al. (2012)	0.39 [0.29, 0.49]	1.24
Chan et al. (2019)	0.23 [0.16, 0.30]	1.28
Ciccarelli et al. (2011)	0.12 [0.09, 0.15]	1.31
Cook et al. (2014)	0.36 [0.25, 0.47]	1.22
Cook et al. (2016)	0.57 [0.46, 0.68]	1.22
Crum-Cianflone et al. (2013)	0.19 [0.14, 0.24]	1.30
Dampier et al. (2017)	0.59 [0.50, 0.68]	1.25
de Almeida et al. (2017)	0.60 [0.48, 0.72]	1.20
de Ronchi, Faranca & Berardi (2002)	0.20 [0.14, 0.26]	1.29
Elham et al. (2020)	0.51 [0.40, 0.61]	1.24
Estiasari et al. (2015)	0.51 [0.40, 0.62]	1.22
Fabbiani et al. (2017)	0.13 [0.04, 0.22]	1.25
Fabbiani et al. (2018)	0.24 [0.17, 0.31]	1.28
Fabbiani et al. (2019)	0.16 [0.12, 0.21]	1.30
Fazeli, Woods & Vance (2019)	0.53 [0.46, 0.60]	1.27
Ferrando et al. (2003)	0.62 [0.54, 0.70]	1.27
Fialho et al. (2013)	0.57 [0.48, 0.67]	1.24
Filho & de Melo (2012)	0.37 [0.23, 0.50]	1.18
Fitri, Rambe & Fitri (2018)	0.75 [0.66, 0.84]	1.25
Foca et al. (2016)	0.47 [0.40, 0.54]	1.28
Foley et al. (2013)	0.22 [0.12, 0.31]	1.25
Gandhi et al. (2011)	0.86 [0.80, 0.92]	1.29
Garvey, Surendrakumar & Winston (2011)	0.19 [0.11, 0.27]	1.27
Gomez et al. (2019)	0.21 [0.17, 0.25]	1.31
Gott et al. (2017)	0.55 [0.45, 0.65]	1.24
Gupta et al. (2007)	0.61 [0.52, 0.69]	1.26
Hestad et al. (2019)	0.37 [0.31, 0.42]	1.29
Holguin et al. (2011)	0.22 [0.11, 0.33]	1.22
Imai et al. (2020)	0.26 [0.22, 0.30]	1.31
Joska et al. (2019)	0.76 [0.70, 0.83]	1.29
Kabuba et al. (2017)	0.35 [0.29, 0.40]	1.29
Kalayjian et al. (2014)	0.23 [0.17, 0.28]	1.30
Kamminga et al. (2017)	0.47 [0.34, 0.60]	1.18
Karlsen, Froland & Reinvang (1994)	0.19 [0.08, 0.30]	1.23
Kelly et al. (2014)	0.70 [0.61, 0.79]	1.26

Kemp et al. (2021)	0.41 [0.33, 0.49]	1.27
Klusman et al. (1991)	0.38 [0.29, 0.47]	1.25
Korten et al. (2021)	0.46 [0.38, 0.53]	1.27
Kupprat et al. (2017)	0.24 [0.17, 0.30]	1.29
Makinson et al. (2020)	0.35 [0.29, 0.42]	1.28
Mogambery et al. (2017)	0.53 [0.45, 0.61]	1.27
Molinaro et al. (2020)	0.59 [0.54, 0.64]	1.30
Munoz-Moreno et al. (2013)	0.61 [0.49, 0.73]	1.21
Nyamayaro et al. (2020)	0.50 [0.42, 0.58]	1.27
Patel et al. (2010)	0.14 [0.09, 0.19]	1.30
Pereda et al. (2000)	0.27 [0.18, 0.36]	1.26
Pumpradit et al. (2010)	0.38 [0.26, 0.49]	1.21
Robbins et al. (2011)	0.80 [0.70, 0.90]	1.24
Robertson et al. (2014)	0.45 [0.42, 0.48]	1.31
Robertson et al. (2019)	0.41 [0.40, 0.43]	1.32
Saini & Barar (2016)	0.32 [0.22, 0.43]	1.23
Salahuddin et al. (2020)	0.39 [0.33, 0.45]	1.29
Salawu et al. (2008)	0.57 [0.44, 0.69]	1.19
Sevigny et al. (2007)	0.69 [0.63, 0.74]	1.30
Simioni et al. (2010)	0.84 [0.79, 0.89]	1.30
Spector et al. (2010)	0.37 [0.30, 0.43]	1.28
Starace et al. (2002)	0.18 [0.14, 0.22]	1.31
Thiyagarajan et al. (2010)	0.18 [0.09, 0.27]	1.25
Tomita et al. (2019)	0.44 [0.36, 0.51]	1.27
Tremont-Lukats, Teixeira & Hernandez (1999)	0.36 [0.25, 0.47]	1.22
Troncoso & de Oliveira Contero (2015)	0.52 [0.43, 0.61]	1.25
Tsegaw et al. (2017)	0.36 [0.33, 0.40]	1.31
Valcour et al. (2004)	0.20 [0.14, 0.25]	1.30
van den Dries et al. (2017)	0.41 [0.29, 0.52]	1.21
Vassallo et al. (2015)	0.30 [0.24, 0.36]	1.29
Vitiello et al. (2007)	0.19 [0.13, 0.25]	1.29
Wang et al. (2013)	0.48 [0.43, 0.54]	1.29
Wojna et al. (2007)	0.68 [0.57, 0.80]	1.21
Xiao et al. (2020)	0.87 [0.83, 0.91]	1.31
Yechoor et al. (2016)	0.38 [0.31, 0.45]	1.28
Zamudio-Rodiguez et al. (2018)	0.66 [0.60, 0.72]	1.29
**Overall**	0.43 [0.38, 0.47]	

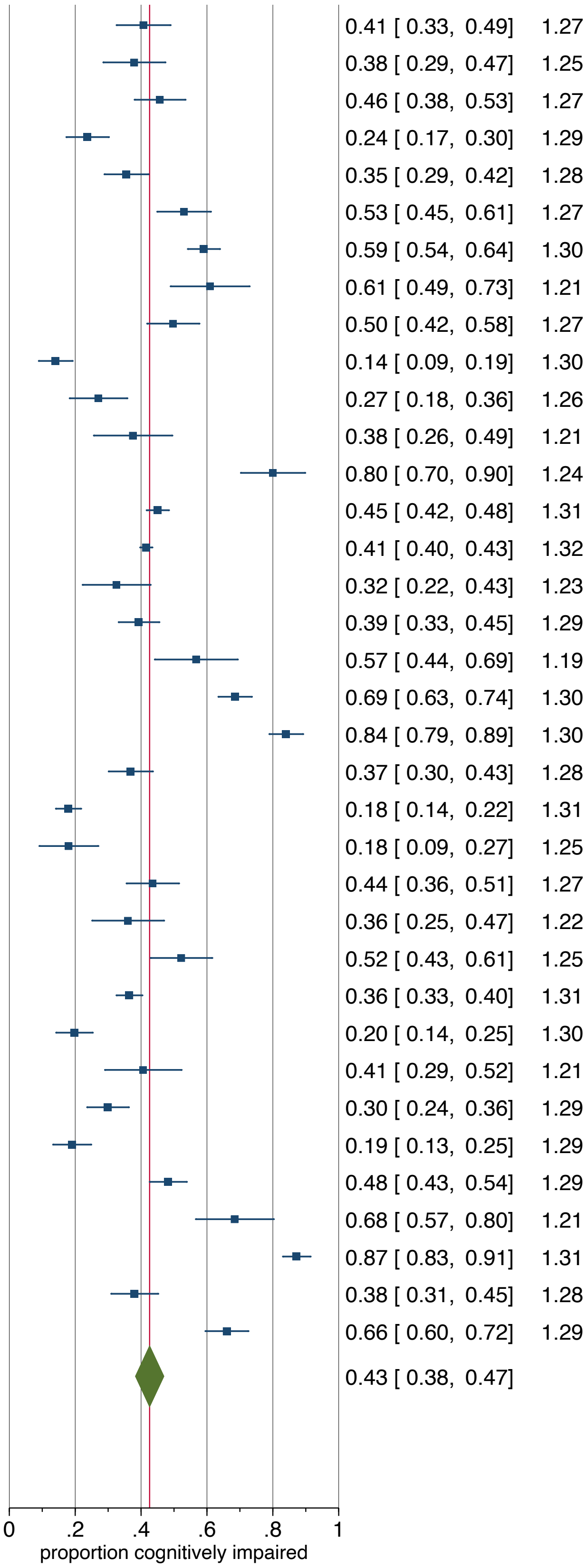

Heterogeneity: $\tau^2 = 0.04$, $I^2 = 97.62\%$, $H^2 = 42.05$

Test of $\theta_i = \theta_j$: $Q(78) = 3619.12$, $p = 0.00$

Test of $\theta = 0$: $z = 18.86$, $p = 0.00$

Random-effects REML model

Supplementary Figure 22: Forest plot including studies without exclusion criteria.

Study	Effect Size with 95% CI	Weight (%)
Araya et al. (2020)	0.36 [0.32, 0.39]	3.39
Becker et al. (2004)	0.20 [0.16, 0.25]	3.37
Bloch et al. (2016)	0.31 [0.25, 0.36]	3.33
Bryant et al. (2015)	0.58 [0.49, 0.67]	3.18
Carvalhal et al. (2016)	0.60 [0.55, 0.65]	3.37
Cross et al. (2013)	0.41 [0.37, 0.45]	3.38
Darling et al. (2021)	0.40 [0.37, 0.43]	3.41
Day et al. (2016)	0.27 [0.22, 0.32]	3.36
Deiss et al. (2019)	0.19 [0.13, 0.24]	3.34
Flatt et al. (2021)	0.47 [0.41, 0.53]	3.31
Greene et al. (2015)	0.47 [0.39, 0.54]	3.23
Haddow et al. (2018)	0.26 [0.22, 0.30]	3.39
Halman et al. (2014)	0.48 [0.37, 0.59]	3.07
Imam (2007)	0.12 [0.08, 0.17]	3.37
Invernizzi et al. (2018)	0.49 [0.37, 0.61]	3.00
McCutchan et al. (2007)	0.27 [0.22, 0.32]	3.35
Mukherjee et al. (2018)	0.23 [0.18, 0.27]	3.37
Munoz-Moreno et al. (2008)	0.48 [0.38, 0.58]	3.14
Nakku, Kinyada & Hoskins (2013)	0.64 [0.61, 0.68]	3.39
Naveed et al. (2021)	0.39 [0.36, 0.42]	3.41
Qiao et al. (2019)	0.12 [0.10, 0.15]	3.42
Sereia et al. (2012)	0.27 [0.18, 0.36]	3.19
Tamargo et al. (2021)	0.15 [0.11, 0.18]	3.40
Underwood et al. (2019)	0.21 [0.18, 0.25]	3.41
Wang et al. (2019)	0.08 [0.06, 0.09]	3.44
Winston et al. (2013)	0.51 [0.47, 0.55]	3.38
Wright et al. (2008)	0.12 [0.09, 0.14]	3.42
Wright et al. (2015)	0.20 [0.17, 0.23]	3.41
Wubetu, Asefa & Gebregiorgis (2021)	0.41 [0.36, 0.46]	3.37
Yusuf et al. (2017)	0.22 [0.18, 0.25]	3.39
**Overall**	0.33 [0.27, 0.38]	

Heterogeneity: $\tau^2 = 0.02$, $I^2 = 98.51\%$, $H^2 = 66.98$

Test of $\theta_i = \theta_j$: $Q(29) = 2221.86$, $p = 0.00$

Test of $\theta = 0$: $z = 11.23$, $p = 0.00$

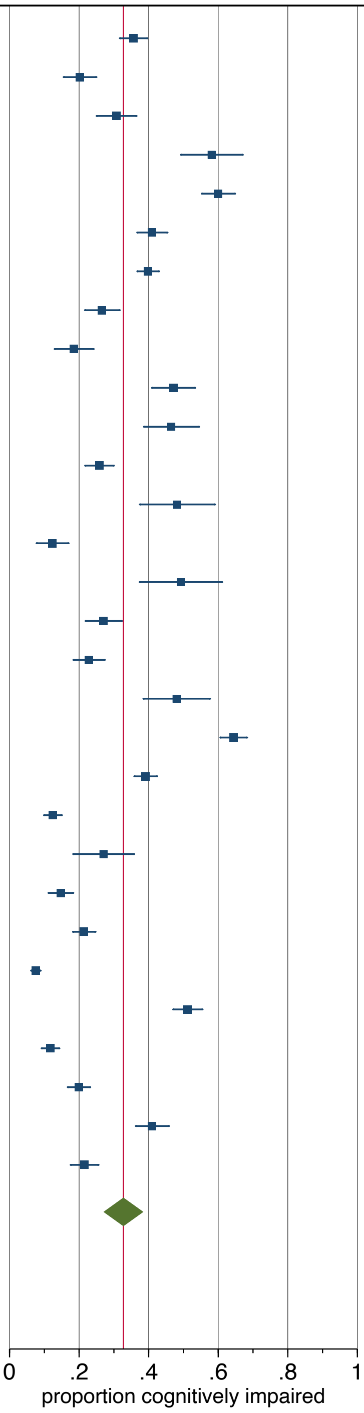

Random-effects REML model

Supplementary Figure 23: Forest plot including studies with unspecified exclusion criteria.

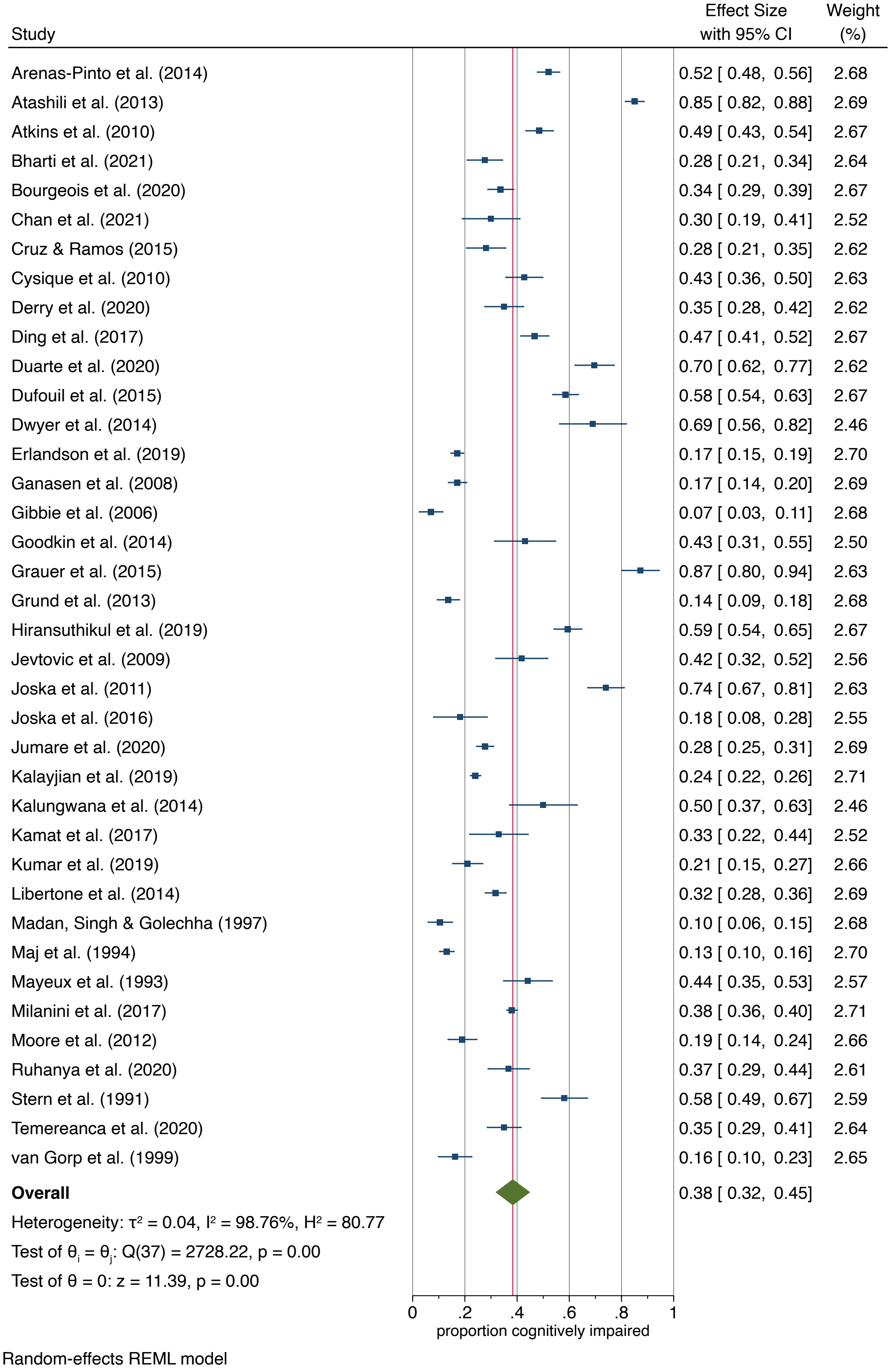

Supplementary Figure 24: Forest plot of study subgroups by NOS score (study quality). Figure made using STATA.

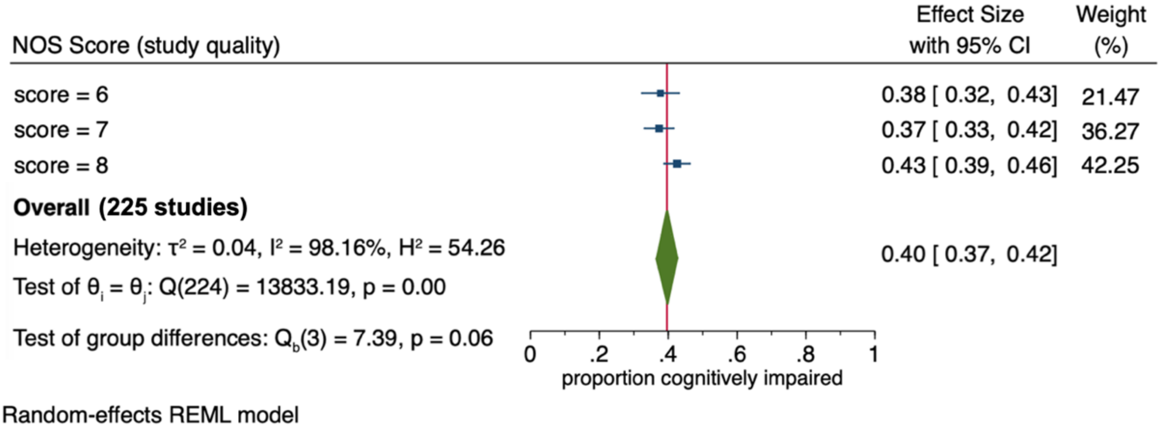

Supplementary Figure 25: Forest plot including studies with NOS scores = 6.

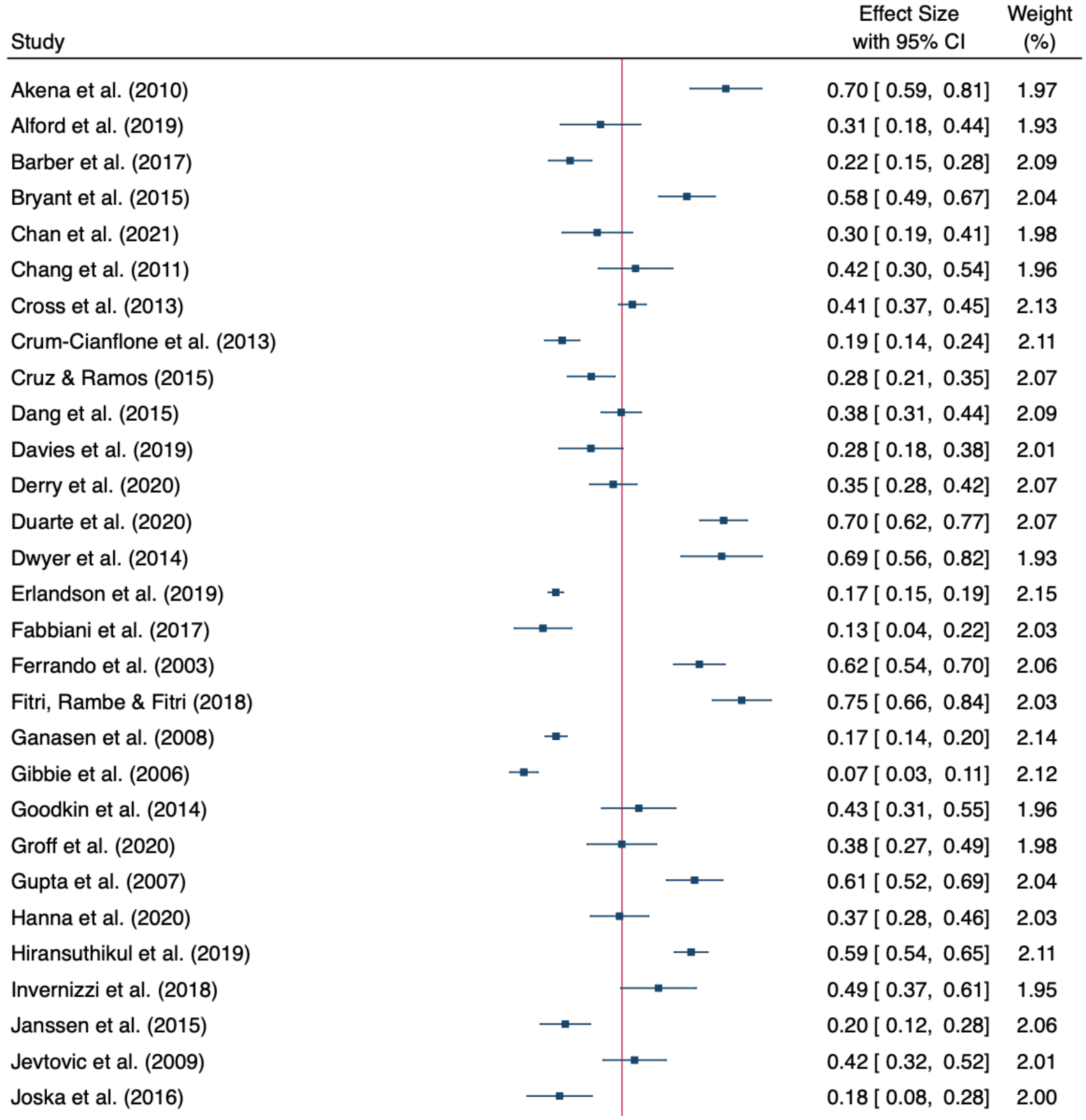

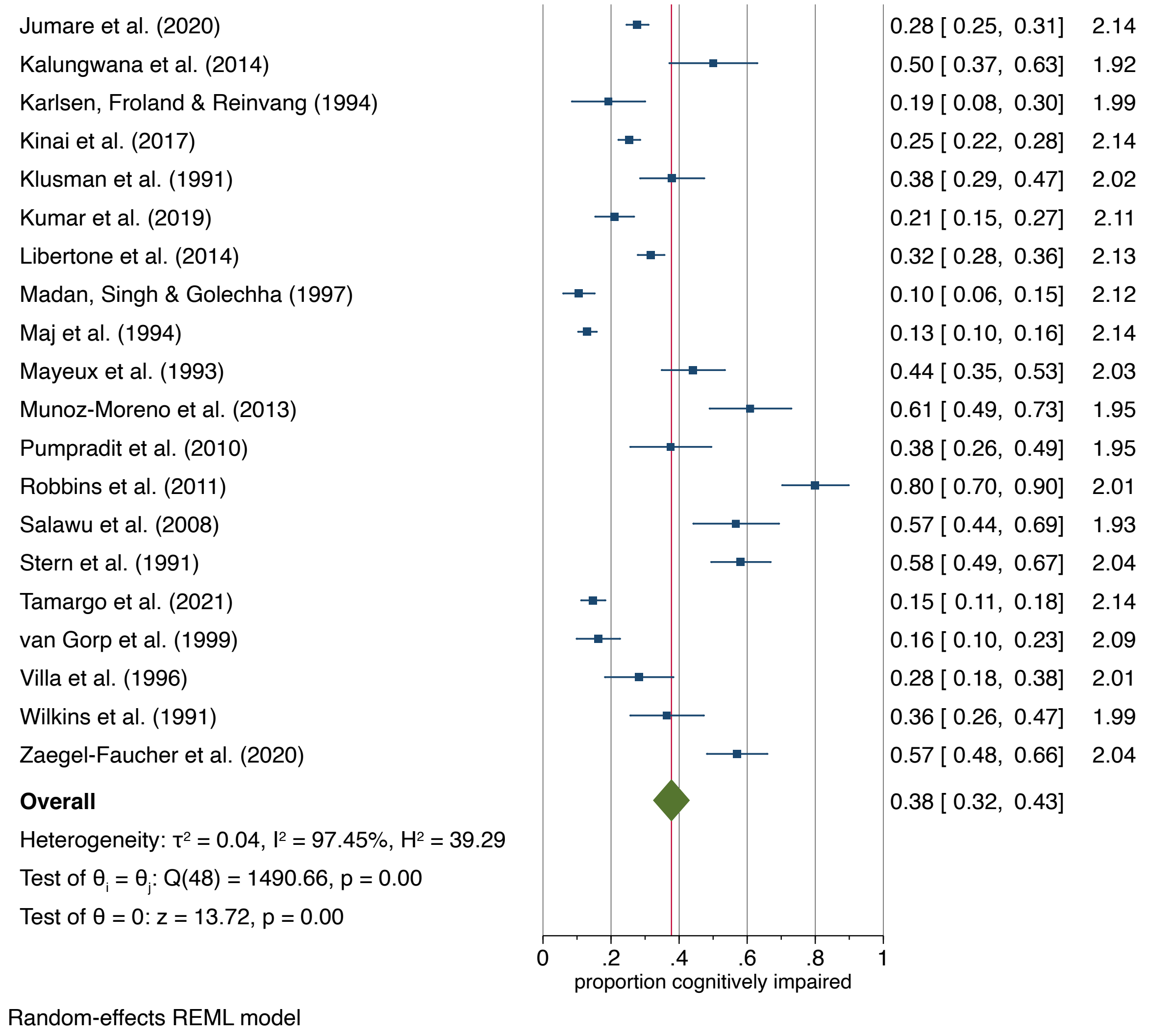

Supplementary Figure 26: Forest plot including studies with NOS scores = 7.

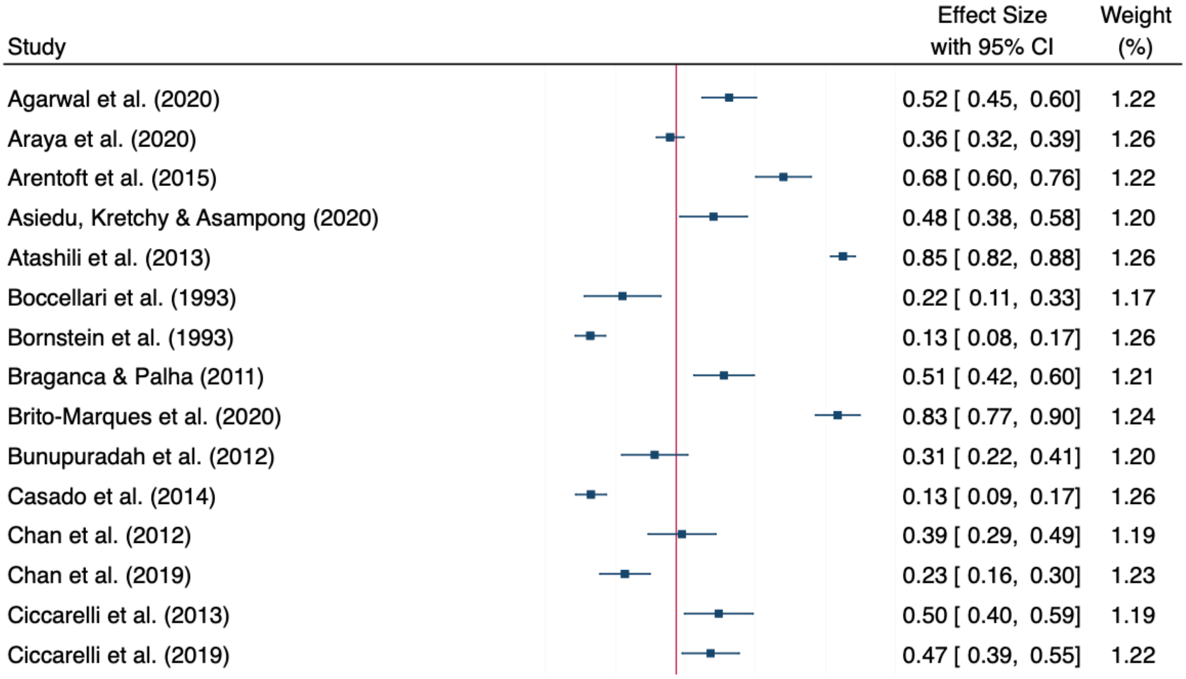

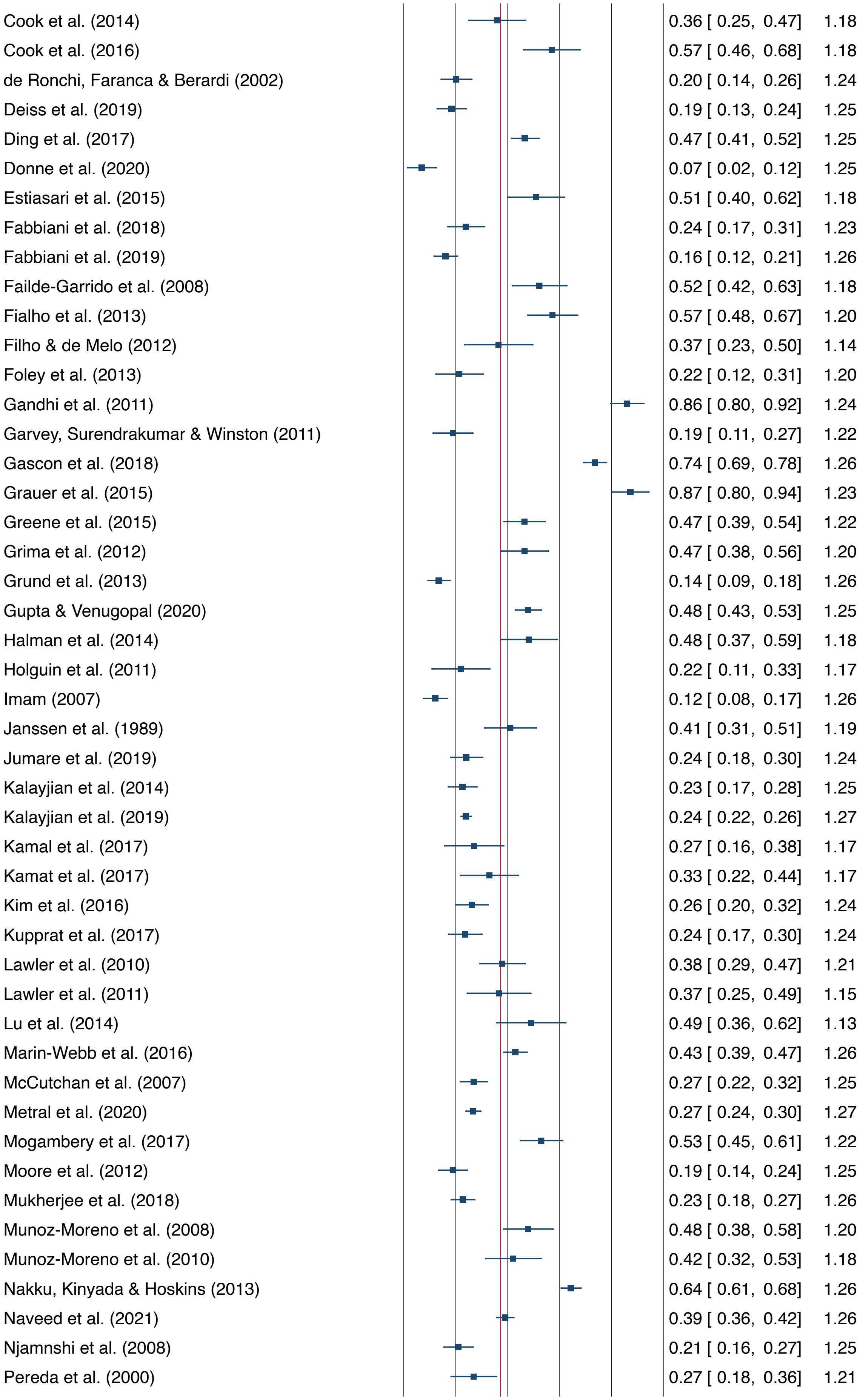
Cook et al. (2014) 0.36 [0.25, 0.47] 1.18
Cook et al. (2016) 0.57 [0.46, 0.68] 1.18
de Ronchi, Faranca & Berardi (2002) 0.20 [0.14, 0.26] 1.24
Deiss et al. (2019) 0.19 [0.13, 0.24] 1.25
Ding et al. (2017) 0.47 [0.41, 0.52] 1.25
Donne et al. (2020) 0.07 [0.02, 0.12] 1.25
Estiasari et al. (2015) 0.51 [0.40, 0.62] 1.18
Fabbiani et al. (2018) 0.24 [0.17, 0.31] 1.23
Fabbiani et al. (2019) 0.16 [0.12, 0.21] 1.26
Failde-Garrido et al. (2008) 0.52 [0.42, 0.63] 1.18
Fialho et al. (2013) 0.57 [0.48, 0.67] 1.20
Filho & de Melo (2012) 0.37 [0.23, 0.50] 1.14
Foley et al. (2013) 0.22 [0.12, 0.31] 1.20
Gandhi et al. (2011) 0.86 [0.80, 0.92] 1.24
Garvey, Surendrakumar & Winston (2011) 0.19 [0.11, 0.27] 1.22
Gascon et al. (2018) 0.74 [0.69, 0.78] 1.26
Grauer et al. (2015) 0.87 [0.80, 0.94] 1.23
Greene et al. (2015) 0.47 [0.39, 0.54] 1.22
Grima et al. (2012) 0.47 [0.38, 0.56] 1.20
Grund et al. (2013) 0.14 [0.09, 0.18] 1.26
Gupta & Venugopal (2020) 0.48 [0.43, 0.53] 1.25
Halman et al. (2014) 0.48 [0.37, 0.59] 1.18
Holguin et al. (2011) 0.22 [0.11, 0.33] 1.17
Imam (2007) 0.12 [0.08, 0.17] 1.26
Janssen et al. (1989) 0.41 [0.31, 0.51] 1.19
Jumare et al. (2019) 0.24 [0.18, 0.30] 1.24
Kalayjian et al. (2014) 0.23 [0.17, 0.28] 1.25
Kalayjian et al. (2019) 0.24 [0.22, 0.26] 1.27
Kamal et al. (2017) 0.27 [0.16, 0.38] 1.17
Kamat et al. (2017) 0.33 [0.22, 0.44] 1.17
Kim et al. (2016) 0.26 [0.20, 0.32] 1.24
Kupprat et al. (2017) 0.24 [0.17, 0.30] 1.24
Lawler et al. (2010) 0.38 [0.29, 0.47] 1.21
Lawler et al. (2011) 0.37 [0.25, 0.49] 1.15
Lu et al. (2014) 0.49 [0.36, 0.62] 1.13
Marin-Webb et al. (2016) 0.43 [0.39, 0.47] 1.26
McCutchan et al. (2007) 0.27 [0.22, 0.32] 1.25
Metral et al. (2020) 0.27 [0.24, 0.30] 1.27
Mogambery et al. (2017) 0.53 [0.45, 0.61] 1.22
Moore et al. (2012) 0.19 [0.14, 0.24] 1.25
Mukherjee et al. (2018) 0.23 [0.18, 0.27] 1.26
Munoz-Moreno et al. (2008) 0.48 [0.38, 0.58] 1.20
Munoz-Moreno et al. (2010) 0.42 [0.32, 0.53] 1.18
Nakku, Kinyada & Hoskins (2013) 0.64 [0.61, 0.68] 1.26
Naveed et al. (2021) 0.39 [0.36, 0.42] 1.26
Njamnshi et al. (2008) 0.21 [0.16, 0.27] 1.25
Pereda et al. (2000) 0.27 [0.18, 0.36] 1.21

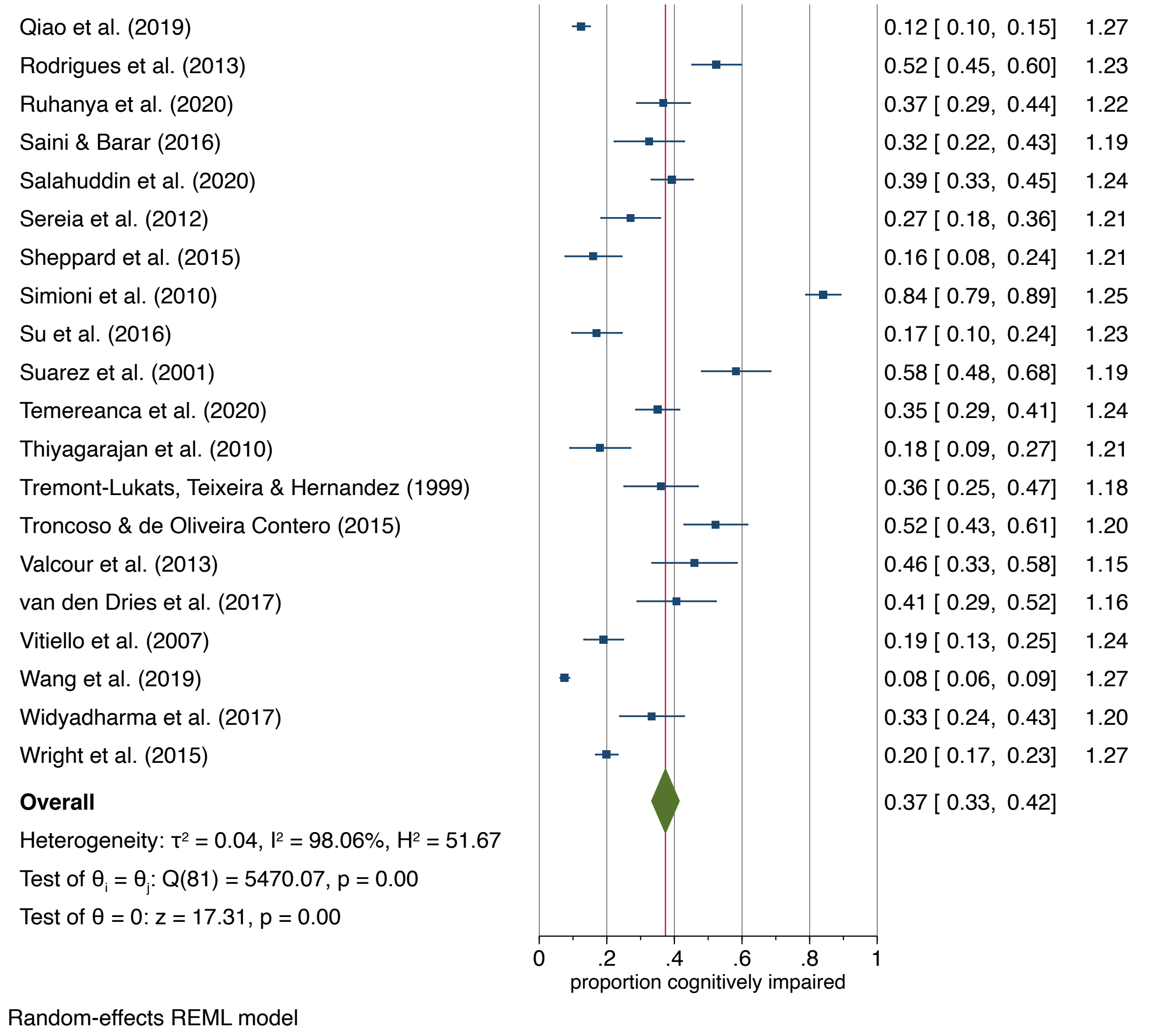

Supplementary Figure 27: Forest plot including studies with NOS scores = 8.

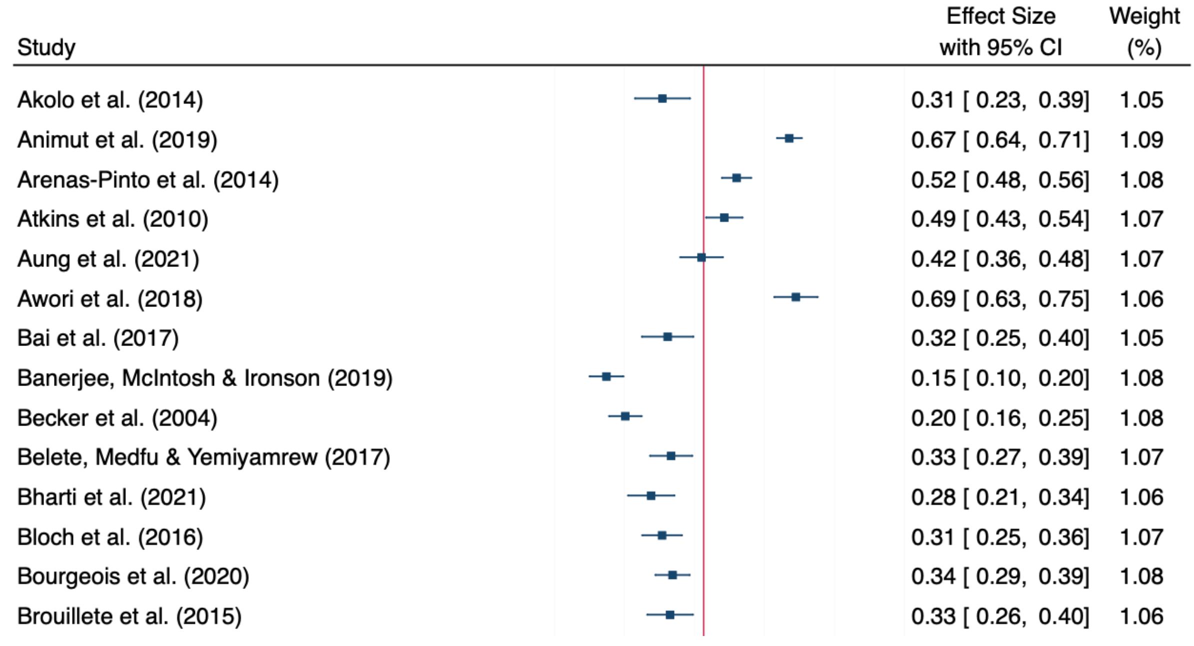

Brouillette et al. (2021)	0.52 [0.46, 0.59]	1.07
Carvalhal et al. (2016)	0.60 [0.55, 0.65]	1.08
Chalermchai et al. (2013)	0.51 [0.40, 0.62]	1.00
Ciccarelli et al. (2011)	0.12 [0.09, 0.15]	1.09
Cysique et al. (2010)	0.43 [0.36, 0.50]	1.06
Dampier et al. (2017)	0.59 [0.50, 0.68]	1.03
Darling et al. (2021)	0.40 [0.37, 0.43]	1.09
Day et al. (2016)	0.27 [0.22, 0.32]	1.08
de Almeida et al. (2017)	0.60 [0.48, 0.72]	0.98
Dufouil et al. (2015)	0.58 [0.54, 0.63]	1.08
Dufour et al. (2013)	0.22 [0.18, 0.27]	1.08
Elham et al. (2020)	0.51 [0.40, 0.61]	1.01
Ene et al. (2016)	0.37 [0.30, 0.43]	1.06
Fazeli, Woods & Vance (2019)	0.53 [0.46, 0.60]	1.05
Flatt et al. (2021)	0.47 [0.41, 0.53]	1.07
Foca et al. (2016)	0.47 [0.40, 0.54]	1.06
Gomez et al. (2019)	0.21 [0.17, 0.25]	1.08
Gott et al. (2017)	0.55 [0.45, 0.65]	1.02
Haddow et al. (2018)	0.26 [0.22, 0.30]	1.08
Heaton et al. (2008)	0.35 [0.29, 0.42]	1.06
Hestad et al. (2019)	0.37 [0.31, 0.42]	1.07
Imai et al. (2020)	0.26 [0.22, 0.30]	1.08
Joska et al. (2011)	0.74 [0.67, 0.81]	1.06
Joska et al. (2019)	0.76 [0.70, 0.83]	1.06
Kabuba et al. (2017)	0.35 [0.29, 0.40]	1.07
Kallianpur et al. (2016)	0.36 [0.34, 0.39]	1.09
Kamminga et al. (2017)	0.47 [0.34, 0.60]	0.96
Kelly et al. (2014)	0.70 [0.61, 0.79]	1.03
Kemp et al. (2021)	0.41 [0.33, 0.49]	1.04
Korten et al. (2021)	0.46 [0.38, 0.53]	1.05
Ku et al. (2014)	0.26 [0.20, 0.32]	1.06
Makinson et al. (2020)	0.35 [0.29, 0.42]	1.06
Matchanova et al. (2020)	0.60 [0.52, 0.68]	1.04
McNamara et al. (2017)	0.51 [0.48, 0.55]	1.08
Milanini et al. (2017)	0.38 [0.36, 0.40]	1.09
Milanini et al. (2020)	0.53 [0.41, 0.64]	1.00
Mohamed, Oduor & Kinyanjui (2020)	0.81 [0.77, 0.85]	1.08
Molinaro et al. (2020)	0.59 [0.54, 0.64]	1.08
Mugendi et al. (2019)	0.12 [0.09, 0.16]	1.09
Namagga et al. (2019)	0.58 [0.53, 0.63]	1.08
Nichols et al. (2013)	0.65 [0.58, 0.71]	1.06
Nyamayaro et al. (2020)	0.50 [0.42, 0.58]	1.05
Odiase, Ogunrin & Ogunniyi (2006)	0.66 [0.59, 0.72]	1.06
Oshinaike et al. (2012)	0.03 [0.01, 0.05]	1.09
Overton et al. (2013)	0.51 [0.44, 0.58]	1.06
Patel et al. (2010)	0.14 [0.09, 0.19]	1.08
Perez-Valero et al. (2013)	0.27 [0.21, 0.34]	1.06

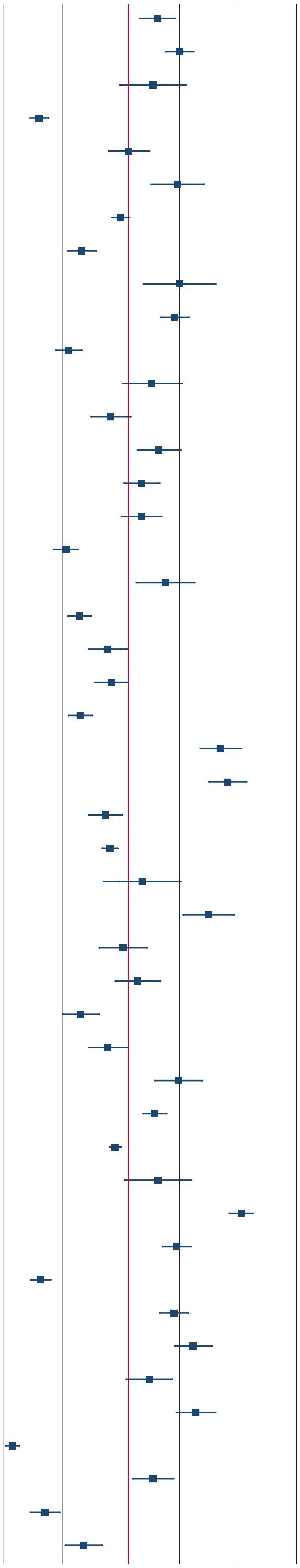

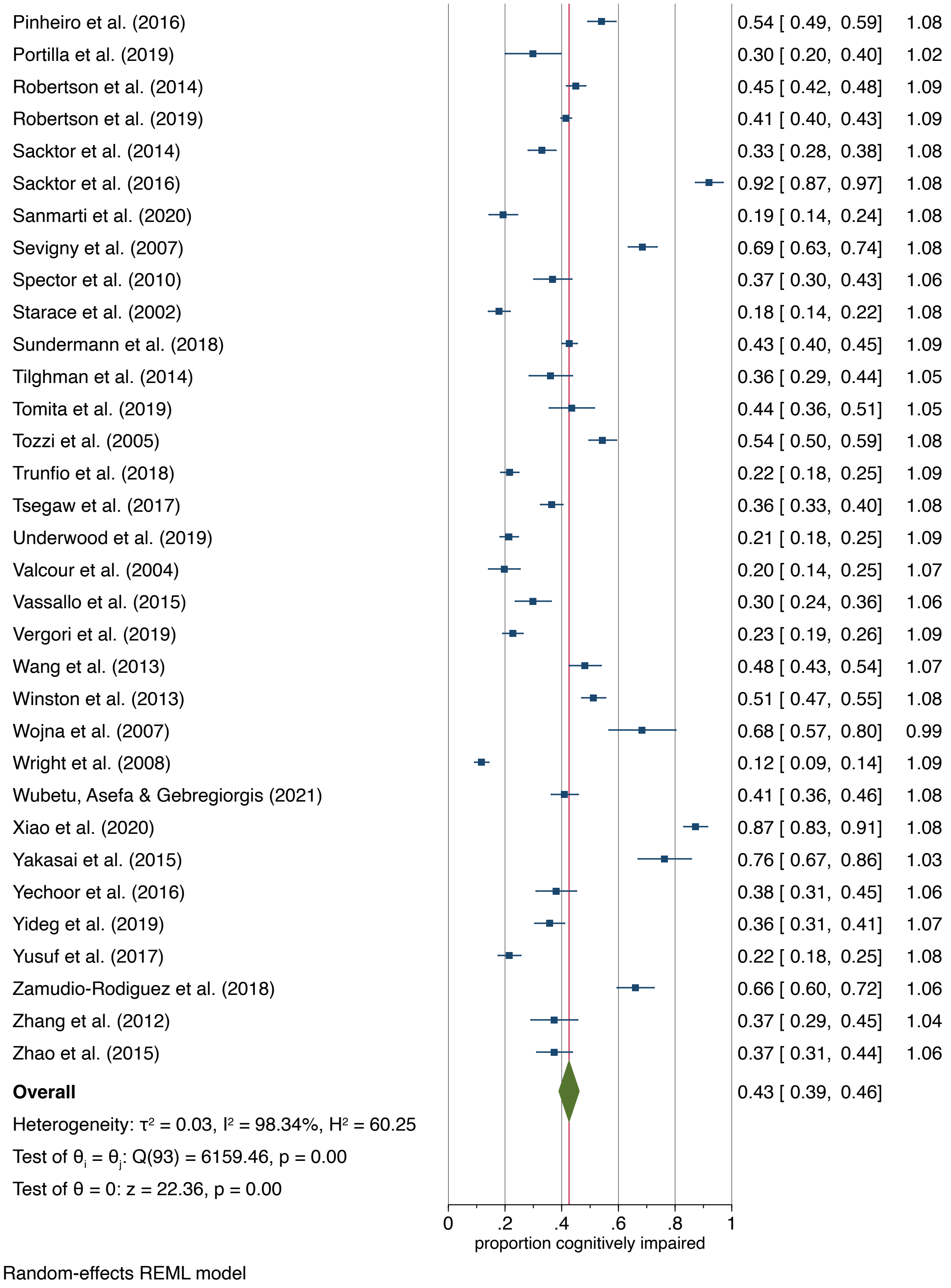
Pinheiro et al. (2016) 0.54 [0.49, 0.59] 1.08
Portilla et al. (2019) 0.30 [0.20, 0.40] 1.02
Robertson et al. (2014) 0.45 [0.42, 0.48] 1.09
Robertson et al. (2019) 0.41 [0.40, 0.43] 1.09
Sacktor et al. (2014) 0.33 [0.28, 0.38] 1.08
Sacktor et al. (2016) 0.92 [0.87, 0.97] 1.08
Sanmarti et al. (2020) 0.19 [0.14, 0.24] 1.08
Sevigny et al. (2007) 0.69 [0.63, 0.74] 1.08
Spector et al. (2010) 0.37 [0.30, 0.43] 1.06
Starace et al. (2002) 0.18 [0.14, 0.22] 1.08
Sundermann et al. (2018) 0.43 [0.40, 0.45] 1.09
Tilghman et al. (2014) 0.36 [0.29, 0.44] 1.05
Tomita et al. (2019) 0.44 [0.36, 0.51] 1.05
Tozzi et al. (2005) 0.54 [0.50, 0.59] 1.08
Trunfio et al. (2018) 0.22 [0.18, 0.25] 1.09
Tsegaw et al. (2017) 0.36 [0.33, 0.40] 1.08
Underwood et al. (2019) 0.21 [0.18, 0.25] 1.09
Valcour et al. (2004) 0.20 [0.14, 0.25] 1.07
Vassallo et al. (2015) 0.30 [0.24, 0.36] 1.06
Vergori et al. (2019) 0.23 [0.19, 0.26] 1.09
Wang et al. (2013) 0.48 [0.43, 0.54] 1.07
Winston et al. (2013) 0.51 [0.47, 0.55] 1.08
Wojna et al. (2007) 0.68 [0.57, 0.80] 0.99
Wright et al. (2008) 0.12 [0.09, 0.14] 1.09
Wubetu, Asefa & Gebregiorgis (2021) 0.41 [0.36, 0.46] 1.08
Xiao et al. (2020) 0.87 [0.83, 0.91] 1.08
Yakasai et al. (2015) 0.76 [0.67, 0.86] 1.03
Yechoor et al. (2016) 0.38 [0.31, 0.45] 1.06
Yideg et al. (2019) 0.36 [0.31, 0.41] 1.07
Yusuf et al. (2017) 0.22 [0.18, 0.25] 1.08
Zamudio-Rodiguez et al. (2018) 0.66 [0.60, 0.72] 1.06
Zhang et al. (2012) 0.37 [0.29, 0.45] 1.04
Zhao et al. (2015) 0.37 [0.31, 0.44] 1.06
Overall 0.43 [0.39, 0.46]
Heterogeneity: $\tau^2 = 0.03$, $I^2 = 98.34\%$, $H^2 = 60.25$
Test of $\theta_i = \theta_j$: Q(93) = 6159.46, p = 0.00
Test of $\theta = 0$: z = 22.36, p = 0.00
0
.2
.4
.6
.8
1
proportion cognitively impaired
Random-effects REML model

Supplementary Figure 28: Bubble plot of cognitive impairment by gender. 221 studies reported the gender of their participants. The size of the data points reflects the study's sample size. Figure made using STATA.

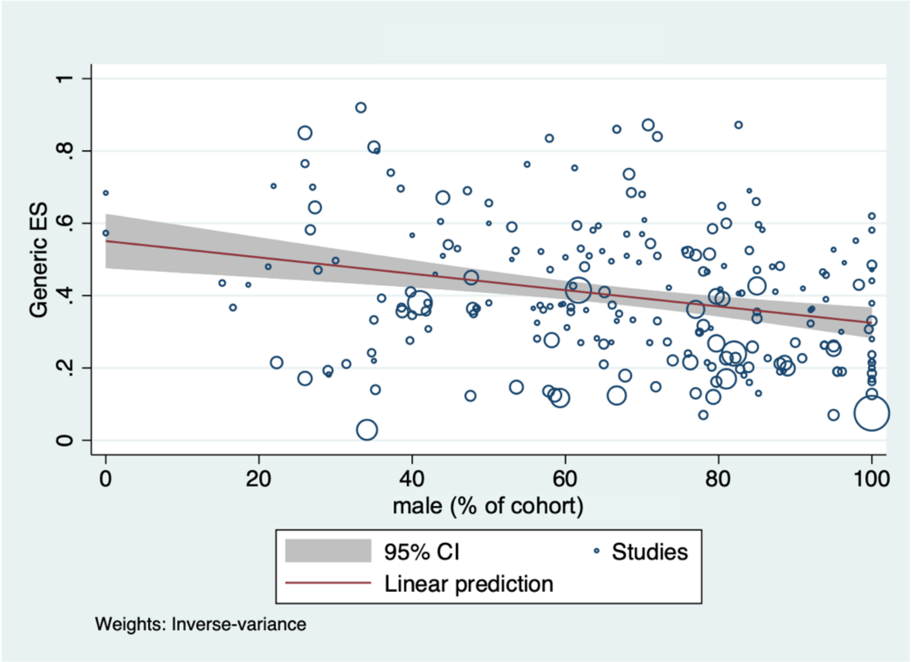

Supplementary Figure 29: Bubble plot of cognitive impairment by ART coverage. 181 studies reported ART coverage among their participants. The size of the data points reflects the study's sample size. Figure made using STATA.

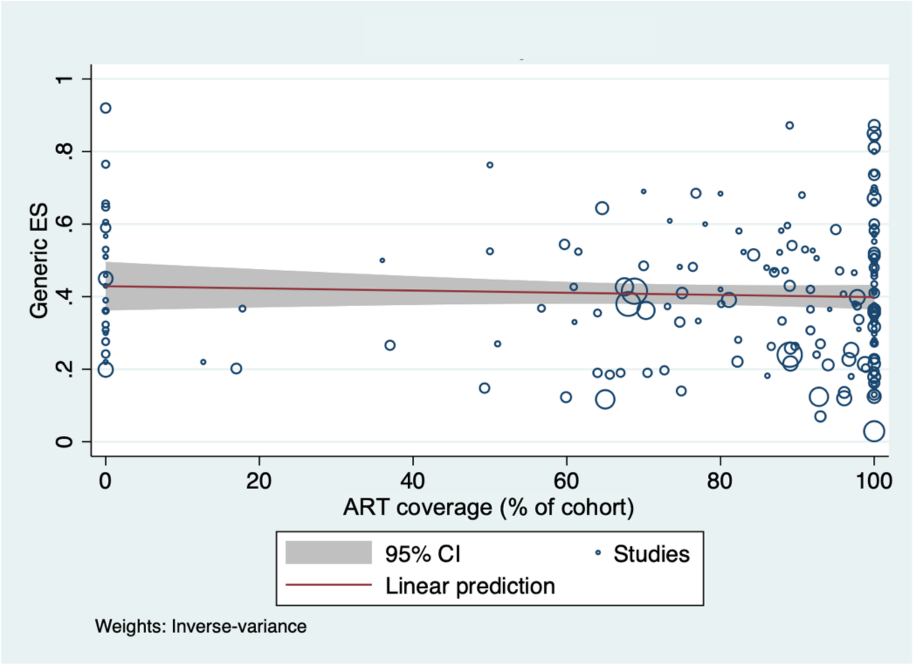

Supplementary Figure 30: Bubble plot of cognitive impairment by age. A) 64 studies reported the age as a median. B) 141 studies reported the age as a mean. The size of the data points reflects the study's sample size. Figure made using STATA.

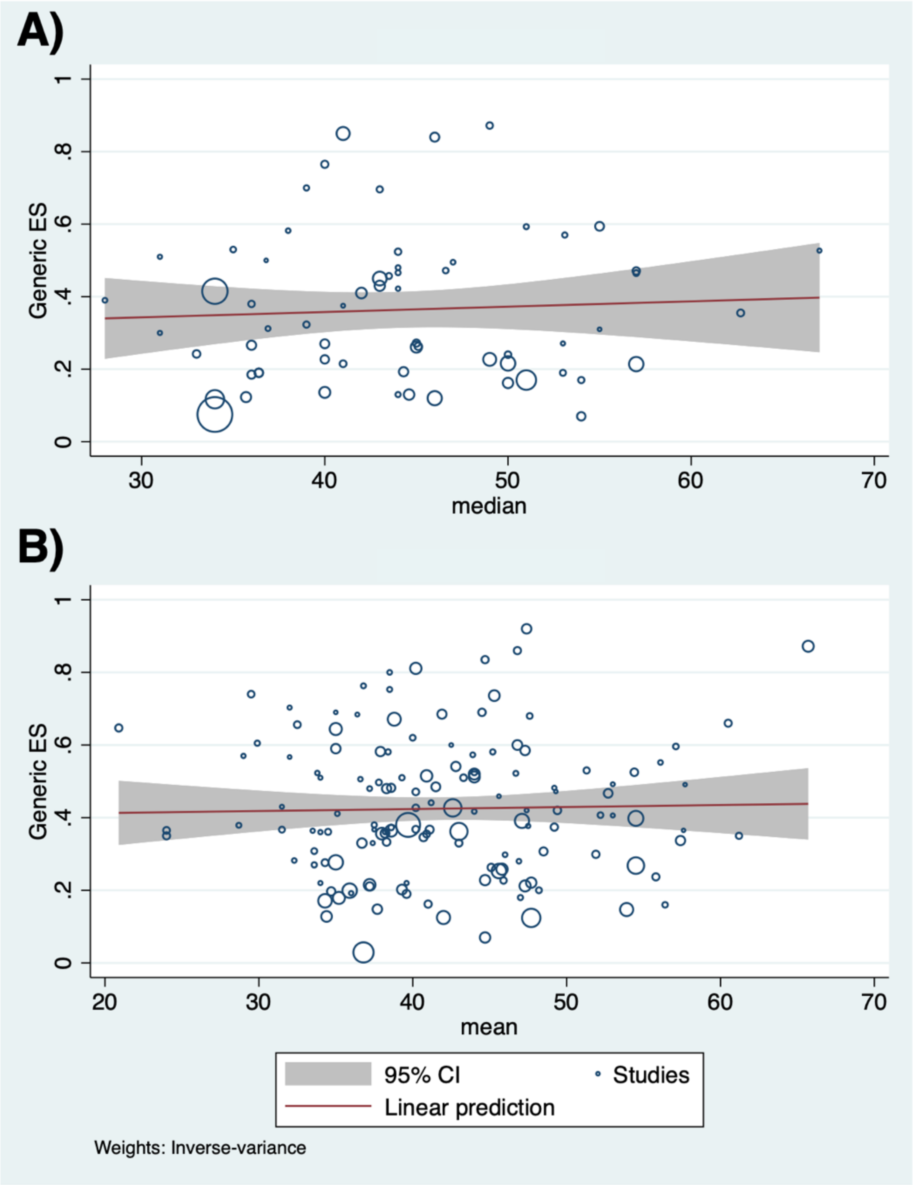

Supplementary Figure 31: Bubble plot of cognitive impairment by publishing year. All studies are included in this plot. The size of the data points reflects the study's sample size. Figure made using STATA.

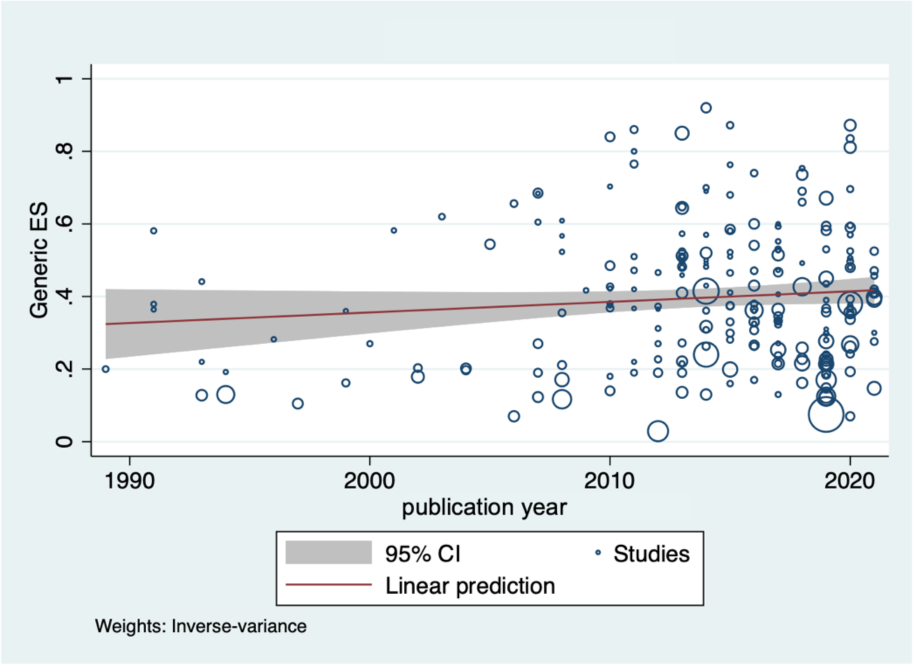

Supplementary Figure 32: Bubble plot of cognitive impairment by sample size. All studies are included in this plot. The size of the data points reflects the study's sample size. Figure made using STATA.

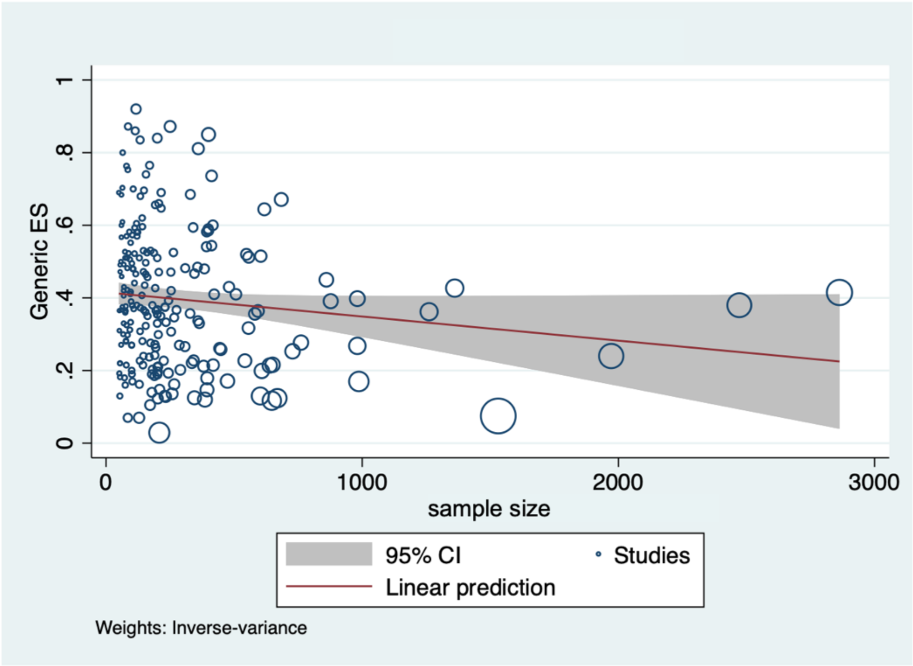

Supplementary Figure 33: Funnel plot to assess publication bias. Each data point represents a study. Figure made using STATA.

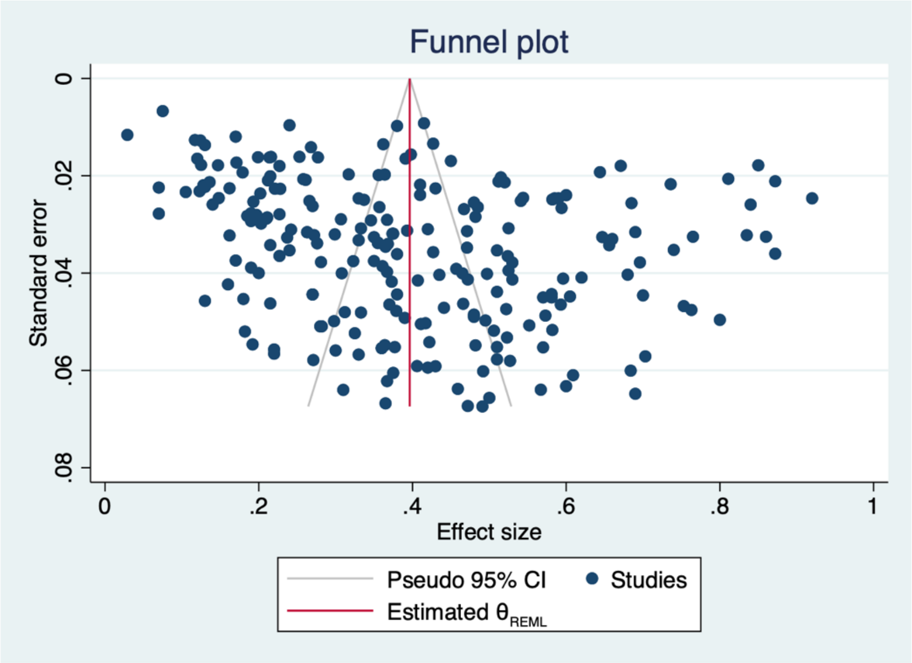

Supplement: Supplemental Digital Content [file aids-37-061-s001.pdf]
